# Growth and neuro-developmental outcomes of probiotic supplemented preterm infants—a systematic review and meta-analysis

**DOI:** 10.1038/s41430-023-01270-2

**Published:** 2023-02-14

**Authors:** Harshad Panchal, Gayatri Athalye-Jape, Shripada Rao, Sanjay Patole

**Affiliations:** 1 Neonatal Directorate, King Edward Memorial Hospital for Women, Perth, WA Australia; 2 School of Medicine, University of Western Australia, Perth, WA Australia; 3 Neonatal Directorate, Perth Children’s Hospital, Perth, WA Australia

**Keywords:** Paediatrics, Nutrition

## Abstract

Gut dysbiosis is associated with sepsis and necrotizing enterocolitis in preterm infants, which can adversely affect long-term growth and neurodevelopment. We aimed to synthesise evidence for the effect of probiotic supplementation on growth and neurodevelopmental outcomes in preterm infants. MEDLINE, EMBASE, EMCARE, Cochrane CENTRAL, and grey literature were searched in February 2022. Only randomized controlled trials (RCTs) were included. Meta-analysis was performed using random effects model. Effect sizes were expressed as standardized mean difference (SMD), mean difference (MD) or risk ratio (RR) and their corresponding 95% confidence intervals (CI). Risk of Bias (ROB) was assessed using the ROB-2 tool. Certainty of Evidence (CoE) was summarized using GRADE guidelines. Thirty RCTs (*n* = 4817) were included. Meta-analysis showed that probiotic supplementation was associated with better short-term weight gain [SMD 0.24 (95%CI 0.04, 0.44); 22 RCTs (*n* = 3721); *p* = 0.02; *I*^2^ = 88%; CoE: low]. However, length [SMD 0.12 (95%CI −0.13, 0.36); 7 RCTs, (*n* = 899); *p* = 0.35; *I*^2^ = 69%; CoE: low] and head circumference [SMD 0.09 (95%CI −0.15, 0.34); 8 RCTs (*n* = 1132); *p* = 0.46; *I*^2^ = 76%; CoE: low] were similar between the probiotic and placebo groups. Probiotic supplementation had no effect on neurodevelopmental impairment [RR 0.91 (95%CI 0.76, 1.08); 5 RCTs (*n* = 1556); *p* = 0.27; *I*^2^ = 0%; CoE: low]. Probiotic supplementation was associated with better short-term weight gain, but did not affect length, head circumference, long-term growth, and neurodevelopmental outcomes of preterm infants. Adequately powered RCTs are needed in this area. Prospero Registration: CRD42020064992.

## Introduction

Survival of preterm infants has significantly improved due to advances in perinatal and neonatal care [1–3]. While there is a trend towards decreased disability rates, a significant proportion of survivors suffer from disabilities. Cheong et al. (Australia) compared the outcomes of extreme premature infants over four longitudinal cohorts: 1991–1992 (*n* = 422), 1997 (*n* = 215), 2005 (*n* = 263), and 2016-2017 (*n* = 252). Survival to 2 years was 53% vs 70% vs 63% vs 73% indicating that the most recent cohort had highest survival rates. The rates of major neurodevelopmental disability were similar across the study epochs (20% vs 26% vs 15% vs 15%). However, survival free of major disability increased steadily over time: 42% vs 51% vs 53% vs 62% (*P* < 0.001) [4]. Pierrat et al. (France) reported the neurodevelopmental outcomes at 2 years corrected age for children born at < 34 weeks in 2011 and evaluated changes since 1997. Among 5170 neonates, survival at 2 years corrected age was 51.7% at 22–26 weeks’ gestation, 93.1% at 27–31 weeks’ gestation, and 98.6% at 32–34 weeks’ gestation. Survival without severe or moderate disabilities increased between 1997 and 2011, from 45.5% to 62.3% at 25–26 weeks’ gestation, but no change was observed at 22–24 weeks’ gestation. At 32–34 weeks’ gestation, the proportion of survivors with cerebral palsy (CP) declined [5]. Bell et al. (US-NICHD) reviewed the outcomes at 22–26 months’ corrected age for extremely preterm infants. The study included 10877 extremely preterm infants born between 2013 and 2018. Outcomes were compared with a similar cohort of infants born in 2008–2012. Survival to discharge increased from 76% in 2008–2012 to 78.3% in 2013–2018. Among 2458 fully evaluated infants, 48.7% (1198/2458) had no or mild, 29.3% (709/2419) had moderate, and 21.2% (512/2419) had severe neurodevelopmental impairment [6].

Hence, the focus has shifted to optimising neurodevelopmental outcomes in this population.

Gut dysbiosis, with decreased abundance of Bifidobacteria and Lactobacilli, and increased abundance of pathogenic bacteria (E. coli, Pseudomonas, Staphylococci, Enterobacter, Clostridia) is known to precede necrotizing enterocolitis (NEC) and late onset sepsis (LOS) in very preterm infants [7–10]. In turn, NEC and LOS are known to be associated with increased mortality and adverse long-term effects on growth and neurodevelopment [11–16]. Evidence from randomised as well as non-randomised studies indicates that probiotic supplementation attenuates dysbiosis and thereby reduces the risk of NEC ≥ Stage II, LOS, and mortality and improves feeding tolerance in preterm infants [17–21]. Probiotic supplementation can improve nutrition through its trophic effects on intestinal villi, peristalsis, and by reducing the risk of NEC, sepsis, and feeding intolerance [19–21]. Through these beneficial effects, probiotics have the potential to improve growth and neurodevelopmental outcomes of preterm infants. In addition, a healthy gut microbiome is known to play an important role in brain development and a healthy gut brain axis [22, 23]. Overall, probiotics have the potential to be neuroprotective considering their direct (e.g.: regulation of gene expression, synthesis of neurotransmitters, and expression of neurotrophic growth factors in the brain, and reduced neuroinflammation due to anti-inflammatory properties) and indirect effects (e.g., reduced risk of NEC, LOS, improved nutrition, and nutrient absorption) [16, 22, 24].

In their prospective observational study in 17 preterm very low birth weight (VLBW) infants, Beghetti et al. reported that bifidobacterial abundance on day 30 was positively correlated with neurodevelopment at 24-months (*p* = 0.001). They also reported that B. *longum* and B. *breve* were absent in the gut microbiota of infants with neurodevelopmental impairment [23].

Given these data, the importance of assessing long-term growth and neurodevelopmental outcomes in probiotic supplemented preterm infants cannot be overemphasised. Upadhyay et al. reported a meta-analysis of data from randomised controlled trials (RCTs) evaluating the effect of prebiotic and probiotics on neurodevelopment in preterm VLBW ( < 1500 g) infants [25]. A total of 7 RCTs were included, of which six involved preterm infants < 33 weeks of gestation. Long-term outcomes were assessed at ≥18–22 months of corrected age in five RCTs. They reported that probiotic supplementation had no effect on the risk of cognitive and motor impairment, CP, and visual, and hearing impairment. The quality of evidence was deemed to be “low” to “very low.” Subsequently, more RCTs have been published evaluating growth and neurodevelopment and hence, we aimed to update the current evidence in this field.

## Methods

Guidelines from the Cochrane handbook (https://training.cochrane.org/handbook/current), and the PRISMA statement were followed for conducting and reporting this systematic review [26]. Ethics approval was not required. Databases MEDLINE via PubMed (www.ncbi.nlm.nih.gov, 1966-February 2022), EMBASE via Ovid (http://ovidsp.tx. ovid.com, 1980 to February 2022), Cochrane Central Register of Controlled Trials (www.thecochranelibrary.com, through February 2022) and EMCARE via Ovid (http://ovidsp.tx.ovid.com, 1980 to February 2022) were searched by two authors. Grey literature was searched using the national technical information services (http://www.ntis.gov/), Open Grey (http://www.opengrey.eu/) and Trove (http://trove.nla.gov.au/). The reference lists of eligible studies and review articles were searched to identify additional studies. No language restriction was applied.

The following terms were used for searching on PubMed: (((“Probiotics”[Mesh]) OR (“Growth and Development”[Mesh] OR “Growth”[Mesh] OR “growth and development” [Subheading])) AND (“Infant, Extremely Premature”[Mesh] OR “Premature Birth”[Mesh] OR “Infant, Premature”[Mesh])) OR “Infant, Small for Gestational Age” OR “Infant, extremely low birth weight”[Mesh] OR Infant, low birth weight”[Mesh] [Majr]. Search was repeated using relevant keywords. Other databases were searched using similar terminologies. Additional studies were identified from the cross references of relevant studies.

RCTs comparing probiotics against placebo/control in preterm infants (<37wk) were included. RCTs assessing prebiotics/synbiotics only were excluded. RCT including probiotic along with prebiotic/synbiotic groups, was included and data from the probiotic group was used for review.

Abstracts of citations from the initial search were read to identify potentially eligible studies. Full-text articles of such identified studies were independently assessed for eligibility by all reviewers. Reference lists from included studies were reviewed to identify additional studies. Following full text review, data were extracted by HP and verified by GAJ. If the included studies had summarised continuous outcomes using median, IQR or range, the formula by Wan et al. [27] was used to convert them into mean and SD.

### Outcomes of interest

1) Short-term growth (weight, length, and head circumference) during hospital stay and/or at discharge. 2) Long-term growth and neurodevelopmental outcomes including cognition, language, CP, deafness, blindness, and autism spectrum disorder (ASD). Pre-planned subgroup analyses included: 1) Gestation < 28 weeks or birth weight < 1000 grams; 2) Single *vs*. multi-strain probiotics.

### Assessment of risk of bias

Risk of bias for each outcome within a study was assessed using the Cochrane ‘Risk of Bias Assessment Tool-2 (ROB-2) [28]. The ROB-2 assesses the RCTs on the following five domains: Bias arising from the randomisation process, Bias due to deviations from intended interventions, Bias due to missing outcome data, Bias in measurement of the outcome and Bias in selection of the reported result. Under each domain, 3–7 signalling questions are asked, based on which judgement is made if the ROB was low/high/some concerns. Finally, a judgement is made on the overall bias rating as low/high/some concerns.

### Statistical analysis

Meta-analysis was performed using Review Manager (version 5.4.1, Cochrane Collaboration, Nordic Cochrane Centre, Copenhagen, Denmark). Random effects model (REM) was used. For data not suitable for meta-analysis, results have been given in a tabular format. Standardized Mean Difference (SMD), Mean Difference (MD) and 95% confidence interval (CI) were calculated for continuous variables. Relative risk (RR) and 95% CI were used for dichotomous variables.

Statistical heterogeneity across studies was quantified using the *I*^2^ statistic. An *I*^2^ value of > 50% was considered to indicate substantial heterogeneity [29]. Publication bias was assessed using Stata 16.0 (StataCorp. 2019. Stata Statistical Software: Release 16. College Station, TX: StataCorp LLC) by Egger’s test, Begg’s test and by inspecting funnel plots wherever there were more than 10 RCTs in the meta-analysis [30–32].

The certainty of evidence (CoE) was rated using the Grading of Recommendations, Assessment, Development, and Evaluation (GRADE) framework [33].

## Results

### Selection, characteristics, and quality of studies

**Fig. 1:**
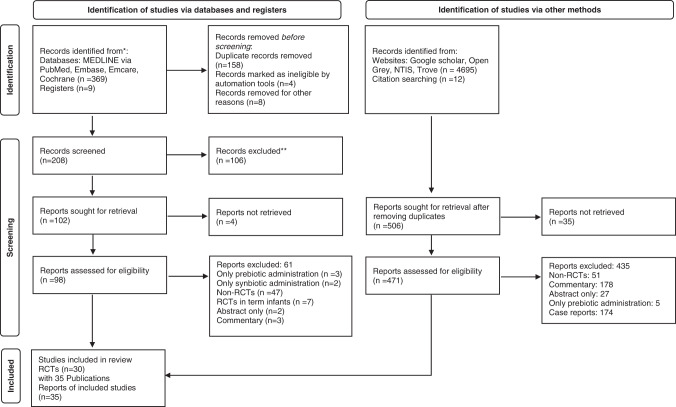
Flowchart showing study selection process. Flowchart summarizing study selection and inclusion processes in this systematic review and metaanalysis, including the reasons for exclusion of all articles that were reviewed.

Literature search retrieved 5085 potentially relevant citations. Reviewers HP and GAJ independently completed initial screening of the titles and abstracts, full-text publications of potential studies and published review articles on probiotics. Of the 378 records identified via databases and registers, 208 records were screened of which 98 reports were assessed for eligibility after screening title/abstract. Of the 4707 records identified from other sources such as websites (Google scholar, Open Grey, NTIS, Trove), 471 reports were assessed for eligibility after title/abstract screen. Discrepancies about inclusion or exclusion of studies and interpretation of data were resolved by discussion among all authors. Included study manuscripts were manually reviewed for references to identify key studies to add to final list of eligible studies. After screening the title/abstract, 569 studies were assessed for eligibility, of which 534 were excluded (Fig. 1). Finally, 30 RCTs (*n* = 4817) with 35 publications were included. Their characteristics are summarized in (Table 1, Supplementary Tables 1 and 2).

**Table 1:** Growth/Neurodevelopmental outcomes in Probiotic supplemented vs. Placebo/control infants.

Study ID	Participants	Sample size, Intervention and Comparison	Dose and duration	Growth/neurodevelopmental outcomes
Studies reporting on neurodevelopment only				
Agrawal et al. [62] 2020 Single centre Australia Follow up of original study by Patole et al. [46]	GA^a^: 28.6 (25.7–30.7; 23.4–32.1) *vs*. 27.7 (26.1–29.1; 23.6–31.7), BW^a^: 1055 (775–1315; 466–1535) *vs*. 960 (810–1180; 540–1735)	*n* = 36/79 vs. 31/80; *Bifidobacterium (B.) breve M-16 V vs*. Placebo (dextrin)	3 billion CFU per day till 37 weeks CGA	Primary: MSEL composite score showed no difference between groups univariately or after adjustment for GA, IUGR, Apgar <7 at 5 min and age at assessment: (adjusted mean effect in probiotic group: −2.7, 95% CI: −8.5 to −3.0, *p* = 0.349). Probiotic group had lower T scores in expressive language domain (adjusted mean effect: −4.5, 95% CI: −9.6 to −0.4, *p* = 0.032), Secondary: No significant differences in the 3Di scores between groups, Tertiary: No significant difference in outcome measures on NEPSY-II, SRS and VABS-II between groups.
Akar et al. [63] 2016 Single centre Turkey	GA^b^: 28.9 ± 2.1 *vs*. 28.6 ± 2.5; *p* = 0.28. BW^b^: 1138 ± 257 *vs*. 1142 ± 267; *p* = 0.89	*n* = 124/200 vs. 125/200; *Lactobacillus (L.) reuteri* vs Control	100 million organisms once daily from starting enteral feeds till discharge	Primary neurodevelopmental outcome: *N* = 124 *vs*. 125 (mean age of 21.7 ± 2.4 months CGA), Moderate to severe CP: 8% *vs*. 8.8%; *p* = 0.83, MDI^a^: 81(49–124) *vs*. 82(53–128); *p* = 0.48, PDI^a^: 80(49–112) *vs*. 79(49–107); *p* = 0.67, NDI: 29% *vs*. 29%; *p* = 0.96, MDI < 70: 20.9 % *vs*. 18.4%; *p* = 0.61, PDI < 70: 19.3% *vs*. 20.8%; *p* = 0.77, Bilateral Blindness: 0% *vs*. 0%, Bilateral Deafness: 0.8% *vs*. 0%; *p* = 0.31
Romeo et al. [64] 2011 Single centre Italy	GA^b^: 33.8 ± 1.8 *vs*. 33.3 ± 1.6 *vs*. 33.3 ± 2.1; *p* = NS. BW^b^: 1998.7 ± 439 *vs*. 1940.7 ± 590 *vs*. 1945.7 ± 465; *p* = NS	*n* = 83 vs. 83 vs 83; *L. reuteri ATCC 55730* vs. *L. rhamnosus ATCC* 53103 vs Control	72 h. of age till 6 weeks/discharge	Neurological outcome at 12 months CGA: using the HINE: 202/ 249 had normal optimality scores (>73), 47 had suboptimal scores (<73) 10/ 83 vs. 13/83 vs. 24/83; *p* < 0.05 for probiotic vs. controls
Studies reporting on both *neurodevelopment and long-term growth*				
Totsu et al. [65] 2018 Multi-centre Japan Follow up of original study by Totsu et al. [47]	GA^b^: 28.7 ± 3.1 *vs*. 28.4 ± 3.0 *p* = 0.568. BW^b^: 1036 ± 289 *vs*. 994 ± 283 *p* = 0.297	*n* = 102/153 vs. 105/130; *B. bifidum OLB 6378 vs*. Placebo (Dextrin)	5 × 10^9^ CFU commenced within 48 h after birth and administered twice daily until the infant’s weight reached 2000g	Primary outcome: Weight at discharge^b^: 2381.8 ± 581.0 *vs*. 2876.8 ± 499.2 g; *p* = NS, HC at discharge^c^: 34.5(33.8–35.5) vs. 34.8(33.7–36.0) cm; *p* = NS Neurodevelopmental outcomes: CP(in those who followed up): 4/100 (4%) *vs*. 10/100 (10%); OR 0.375(95% CI: 0.114:1.238); *p* = 0.108; Developmental DQ18 score^b^: 90.6 ± 12.5 (*n* = 54) *vs*. 91.1 ± 14.4 (*n* = 65), partial correlation coefficient (PCC): −0.443(95% CI: −5.384 to 4.499); *p* = 0.859; DQ 18 < 85: 24/89 (27%) vs. 32/79 (41%), OR 0.542(95% CI: 0.283–1.038); *p* = −0.065, Subgroup analysis: more favourable development was noted in probiotic vs placebo group, among the infants with a birth weight ≥1000 g, gestational age ≥28 weeks, caesarean delivery, antenatal steroid use, female sex or ≥13 days until full enteral feeding Other outcomes: physical development at 18 months of age: Weight^b^: 9.3 ± 1.7 (*n* = 98) vs. 9.2 ± 1.2 kg (*n* = 103); PCC: 0.177 (−0.277 to 0.581); *p* = 0.39, HC^b^: 46.3 ± 2.2 (*n* = 80) vs. 46.5 ± 1.8 cm (*n* = 93): PCC: −0.259(95% CI: −0.864 to 0.347); *p* = 0.401, BL^b^: 77.1 ± 4.3 (*n* = 97) vs. 77.2 ± 4.2 cm (*n* = 103), PCC: −0.148 (95%ci: −1.333 TO 1.038); *p* = 0.806
Jacobs et al. [66] 2017 PRO-PREMS Study Multi-centre Australia, New Zealand Follow up of original study by Jacobs et al. [50]	GA^b^: 27.6 ± 2.0 vs. 27.6 ± 1.9, BW^b^: 1042 ± 267 vs. 1027 ± 261	*n* = 373/548 vs. 362/551; *B*. *infantis, Streptococcus thermophilus and B. lactis vs*. Placebo (maltodextrin)	1 × 10^9^ CFU administered from birth until discharge home or term corrected age, whichever was sooner	Primary neurodevelopmental outcome: Survival without neurosensory impairment at 2 years corrected age: 281/373 (75.3%) *vs*. 271/362 (74.9%); relative risk (RR) 1.01 (95% CI 0.93 to 1.09); *p* = 0.88, Major neurosensory impairment:56/337 (16.6%) *vs*. 56/327 (17.1%), RR: 0.97 (95% CI: 0.69–1.36); *p* = 0.86, Moderate/severe cerebral palsy (Gross Motor Function Classification System score 2–5): moderate CP: 8% *vs*. 9.2%, severe CP: 0.3% *vs*. 1.5%, motor impairment (Bayley-III Motor Composite Scale < –2 SD or Movement Assessment Battery for Children <15th centile if > 42 months’ CA): 9.3% *vs*. 7.4%, RR 1.25 (95% CI: 0.75–2.07); *p* = 0.4, cognitive impairment (Bayley-III Composite Cognitive or Language Scales < –2 SD or WPPSI FSIQ < –2 SD if >42 months’ CA): Cognitive impairment: 11.6% *vs*. 12.4%, RR 0.93 (95% CI: 0.62 to 1.41); *p* = 0.74, WPPSI scores: FSIQ^b^: 106.0 ± 21.6, *n* = 37 (probiotic group); MD: 1.3 (−8.3 to 14.1; *p* = 0.79), FSIQ < 70: 5.4% (*n* = 37) *vs*. 4% (*n* = 25); MD: 1.35 (0.1 to 14.1; *p* = 0.8), BSID-III cognitive scores^b^: 100.4 ± 17.1, *n* = 299 *vs*. 99.2 ± 15.1, *n* = 298; Mean difference (MD): 1.2 (95% CI −1.4 to 3.8; *p* = 0.36), BSID-III motor scores^b^: 102.3 ± 11.6, *n* = 299 *vs*. 100.7 ± 16.8, *n* = 296; MD: 1.6 (−1.1 to 4.3; *p* = 0.24), BSID-III language scores^b^: 98.3 ± 16.8, *n* = 289 *vs*. 98.5 ± 18.1, *n* = 281; MD: −0.3 (−3.1 to 2.6; *p* = 0.86), blindness: 0.3% *vs*. 0% or deafness: 0.6% *vs*. 3.4% RR: 0.18 (95%CI: 0.04–0.8; *p* = 0.01) Secondary outcomes: Growth at mean age of 30 months^b^: Z scores (*n* = 329 vs. 321): Weight: −0.6 ± 1.3 *vs*. −0.6 ± 1.3, Height: −0.2 ± 1.3 *vs*. −0.2 ± 1.2, HC: −1.2 ± 1.3 *vs*. −1.1 ± 1.4
Sari et al. [67] 2012 Single centre Turkey Follow up of original study by Sari et al. [54]	GA^b^: 29.7 ± 2.5 vs. 29.8 ± 2.3; *p* = 0.648 BW^b^: 1241 ± 264 vs. 1278 ± 273; *p* = 0.380	*n* = 86/110 vs. 88/111; *L*. *sporogenes vs*. Control	0.35 × 10^9^ CFU/ day starting with the first feed continued until discharge	Primary outcome: Growth^b^: Wt: 10.5 ± 1.7 *vs*. 10.5 ± 1.7 kg; *p* = 0.92, BL: 79.4 ± 7.8 *vs*. 81.0 ± 5.3 cm; *p* = 0.326, HC: 47.5 ± 6.5 *vs*. 46.7 ± 1.8 cm; *p* = 0.53 Neurodevelopmental outcomes: CP (4.7% vs. 2.3%; *P* = 0.441), Visual impairment (1.2% vs. 2.3%; *p* = 1), Hearing impairment (1.2% vs. 1.1%; *p* = 1), Mental development index (MDI): 90.7 ± 15.5 vs. 90.4 ± 14.5; *p* = 0.887), MDI < 70 (14% vs. 11.4%; *p* = 0.607), Psychomotor development index (PDI)^b^: 95.4 ± 17.2 *vs*. 93.2 ± 16.4; *p* = 0.394, PDI < 70 (10.5% vs. 10.2%; *p* = 0.959), Overall NDI (18.6% vs. 17%; *p* = 0.788)
Chou et al. [68] 2010 Single centre Taiwan	GA^b^: 28.5 ± 2.3 vs. 28.5 ± 2.3; *p* = .90 BW^b^: 1103.6 ± 232.4 vs. 1097.2 ± 231.4; *p* = 0.80	*n* = 153/180 vs. 148/187; *Infloran* with *L. acidophilus* + *B. infantis vs*. Control	*Infloran* 125 mg/kg/dose (*L. acidophilus* 1 billion CFU + *B. infantis* 1 billion CFU) administered with starting enteral feeds and continued till discharge	Primary outcome: *n* = 153 *vs*. 148: Death/ NDI: 29.4% *vs*. 33.1%; *p* = 0.1, Death: 5.2% *vs*. 16.2 %; *p* = 0.0002, CP: 5.2% *vs*. 2%; *p* = 0.5; Visual impairment: 0.6% *vs*. 2.7%; *p* = 0.2; Deafness: 1.3% *vs*. 0.6%; *p* = 1; BSID-II MDI (mean ± 2 SD): 87.9 ± 18.1 *vs*. 88.±18.4; *p* = 0.8, MDI < 70: 14.3% *vs*. 18.2%; *p* = 0.3, BSID-II PDI^b^: 86.4 ± 18.6 *vs*. 87.9 ± 17.1; *p* = 0.3, PDI < 70: 12.4% *vs*. 12.25; *p* = 0.1 Other outcomes: Growth at 3 years age^b^: Weight: 11.2 ± 1.9 *vs*. 11.9 ± 1.7 kg; *p* = 0.9, height: 84.4 ± 5.2 *vs*. 84.4 ± 5.2 cm; *p* = 1, HC: 46.2 ± 1.7 *vs*. 46.3 ± 3.7; *p* = 1
Studies reporting on *growth only*				
Spreckels et al. [34] PROPEL trial 2021 Multi centre Sweden	GA^b^: 25.5 ± 1.2 *vs*. 25.6 ± 1.2; *p* = 0.75. BW^b^: 724 ± 131 *vs*. 754 ± 143; *p* = 0.25	*n* = 68/72 vs. 66/69; *L. reuteri DSM 17938 vs*. Placebo: (Maltodextrin)	1.25 ×10^8^ CFU/day starting from day1–3 upto 36 weeks PMA	Growth: Head growth: median (95% CI): −1.11(−0.86 to −1.35) *vs*. −1.78(−1.5 to −2.06); females had improved length growth until 4 weeks (*p* = 0.007) and improved head growth until 2 (*p* = 0.045) and 4 weeks of age (*p* = 0.013).
Cui et al. [35] 2019 Single centre China	GA^b^: 32.85 ± 1.39 *vs*. 32.56 ± 1.41; *p* = 0.3206. BW^b^: 1682 ± 109.03 *vs*. 1714 ± 127.11; *p* = 0.1984	*n* = 45/57 vs. 48/57; *L. reuteri DSM 17938 vs*. Control	1 × 10^8^ CFU (5 drops) once daily, start with first feed until hospital discharge. Minimum duration: 7 days	Primary outcome: Growth^b^: Wt gain: 14.55 ± 3.07 *vs*. 10.12 ± 2.80 g/day; *p* = 0.000, HC gain: 0.0760 ± 0.0157 *vs*. 0.0681 ± 0.0108 cm/day; *p* = 0.007, BL gain: 0.1878 ± 0.0151 *vs*. 0.1756 ± 0.0166 cm/day; *p* = 0.000
Oshiro et al. [36] 2019 Single centre Japan	GA^b^: 28.1 ± 3.1 *vs*. 28.2 ± 3.3, BW^b^: 1049 ± 302 *vs*. 1002 ± 289	*n* = 17 vs. 18; *B. breve vs*. Placebo: NS	2.5 × 10^8^ CFU once a day till discharge	Primary outcome: Wt gain: The probiotic group showed significantly larger cumulative body weight gain by 8 weeks (*p* < 0.05) (graphical data only)
Wejryd et al. [37] PROPEL trial 2019 Multi Centre Sweden	GA^b^: 25.5 ± 1.2 *vs*. 25.5 ± 1.3; *p* = 0.95. BW^b^: 731 ± 129 *vs*. 740 ± 148; *p* = 0.71	*n* = 68 vs. 66; *L. reuteri DSM 17938 vs*. Placebo (maltodextrin)	Daily *L. reuteri*; 1.25 ×10^8^ bacteria (0.2 mL drops) started within three days after birth until 36 weeks	Other outcomes: Growth at 28 days: Wt gain: 340.5 ± 216 vs. 323.8 ± 167 g; BL gain: 3.26 ± 1.5 vs. 3.23 ± 1.5 cm; HC gain: 2.22 ± 1.0 vs. 1.75 ± 1.2 cm. Growth at 36 weeks: Wt gain: 1565 ± 361 vs. 1603 ± 369 g; BL gain: 10.5 ± 2.4 vs. 10.7 ± 2.3 cm; HC gain: 8.2 ± 1.4 vs. 7.9 ± 1.9 cm Growth at 2 weeks: Wt: 868 ± 165 vs. 896 ± 168 g; BL: 34.1 ± 2.2 vs. 34.5 ± 2.2 cm; HC: 23.6 ± 1.2 vs. 23.6 ± 1.3 cm. At 4 weeks: Wt: 1075 ± 244 vs. 1055 ± 243 g; BL: 35.8 ± 1.8 vs. 35.9 ± 2.3 cm; HC: 25.1 ± 1.5 vs. 24.9 ± 1.4 cm HC: Z-score decreased in both groups from birth to day 28 of life, lesser rate of decrease in the *L. reuteri* vs. placebo group: 1.2 SD (95%CI: 1.4: 1.0) *vs*. 1.7 SD (95%CI: 2.0:1.5); *p* = 0.001. From birth-day 28: HC increased by 2.3 cm (95% CI: 2.0–2.5) vs. increase by 1.8 cm (95% CI: 1.5–2.1) in the L. *reuteri vs*. control group (*p* = 0.01). Girls showed better increase in HC: [1.2 SD (95% CI 1.4: 1.0] *vs*. boys [1.7 SD (95%CI: 1.9: 1.5); *p* < 0.001].
Indrio et al. [38] 2017 Multi centre Italy	GA^b^: 30.2 ± 1.2 *vs*. 30.1 ± 1.2, BW^b^: 1471.5 ± 455.1 *vs*. 1406.6 ± 536.4	*n* = 30 vs. 30; *L. reuteri DSM 17938 vs*. Placebo: mixture of sunflower oil and MCT oil	once a day at a dose of 1×10^8^ CFU until 30 days of life	Primary outcome: Weight at end of the study period: 1955.3 ± 653.4 vs. 1737.6 ± 512 g; *p* < 0.05
Shashidhar et al. [39] 2017 Single centre India	GA^b^: 31.2 ± 2.1 *vs*. 31 ± 2.1, BW^b^: 1256 ± 185 *vs*. 1190 ± 208	*n* = 48/52 vs. 48/52; *L. acidophilus, L. rhamnosus, B. longum and Saccharomyces (S.) boulardii*; *vs*. Control	once a day at a dose of 1.25×10^9^ CFU until discharge	Other outcomes: Weight gain/week^b^: 31.1 ± 27 vs. 39.5 ± 32.3 g; *p* = 0.2
Sukanyaa S et al. [40] 2017 Single centre India	VLBW infants with BW ≤ 1,500, GA < 34	*n* = 45(total); *L. acidophilus, B. infantis, S. boulardi vs*. Control	Half sachet (>1 million CFU) twice daily diluted with EBM, duration: NS	Primary outcomes: Average weight gain was significantly better in probiotic group, monitored over period of 1 month of age (details NS): (Mean Difference/MD: 0.230 ± 0.11 g; 95% CI: −0.796 to −0.251; *p* < 0.000)
Hays et al. [41] 2016 Multi-centre France	GA^c^: 29.0 (28.1; 30.1) *vs*. 29.4 (27.9; 30.6), BW^c^: 1170 (1000; 1320) *vs*. 1170 (1055; 1370)	*n* = 145/147 vs. 52; Group 1, *n* = 50 (*B. lactis*); Group 2, *n* = 49 (*B. longum*); Group 3, *n* = 48 (*B. lactis* + *B. longum*) *vs*. Placebo (maltodextrin).	10^9^ CFU/day started before end of first week of life, and continued for four (if birth GA < 29 weeks) or six (if birth GA < 28 weeks) weeks	Primary outcome: Postnatal growth: no significant differences in mean body weight at end of supplementation^b^: 1875 ± 14 *vs*.1906 ± 23 g, *p* = 0.25. Average daily weight gain: 15.9 ± 4.1 *vs*. 16.6 ± 3.1 g/kg/day; *p* = 0.17. No statistically significant differences in anthropometric measures (weight for age, length for age and HC for age at 41 weeks corrected z-score; *p* = NS) or body composition analysis at 41 weeks between the intervention groups.
Xu et al. [42] 2016 Single centre China	GA^b^: 33 ± 0.72 *vs*. 33 ± 1.04, BW^b^: 1947 ± 54 *vs*. 1957 ± 51.	*n* = 51/63 vs. 49/62; S. *boulardii CNCM I-745 vs*. Control	10^9^ CFU/kg of *S. boulardii CNCM I-745*, administered twice daily *vs*. Control. Supplement ceased at day 28 or at hospital discharge Minimal duration: 7 days	Primary outcome: Weight gain^b^: 16.14 ± 1.96 *vs*. 10.73 ± 1.77 g/day; *p* = 0.02, HC gain^b^: 0.74 ± 0.03 *vs*. 0.72 ± 0.04 cm/week; *p* = 0.67, Linear growth^b^: 0.89 ± 0.04 *vs*. 0.87 ± 0.04 cm/week; *p* = 0.17
Choudhury et al. [43] 2015 Single centre Bangladesh	GA^b^: 31.9 ± 1.32 *vs*. 32.04 ± 1.26; *p* = 0.76, BW: 1000 - <1800. *p* = NS	*n* = 28/30 vs. 29/35; TS6 Probiotic (Eight viable strains mixture of *Lactobacillus* and *Bifidobacterium* (20 billion/2 gram) *vs*. Control	Starting dose 1.65 billion CFU and increased to 3.3 billion CFU when feed volume reached 2 ml/feed, continued till attainment of full enteral feed	Primary outcomes: Weight at discharge^b^: 1458.83 ± 209.70 *vs*. 1363.86 ± 216.23 g; *p* = 0.07
Dilli et al. [44] 2015 Multi centre Turkey	GA^b^:28.8 ± 1.9 vs. 28.2 ± 2.2; BW^b^: 1236 ± 212 *vs*. 1147 ± 271	*n* = 100 vs. 100; *B. lactis vs*. Placebo (maltodextrin)	Daily *B. lactis*, 5 ×10^9^ CFU until discharge or death (maximum of 8 weeks, whichever came first)	Other outcomes: Growth velocity^b^: Wt gain: 230 ± 74 *vs*. 227 ± 100 g/kg/week; *p* = 0.09, BL gain: 1.3 ± 0.7 *vs*. 1.2 ± 0.6 cm/week; *p* = 0.04, HC gain: 1.1 ± 0.5 *vs*.1.3 ± 0.7 cm/week; *p* = 0 .06. Weight at discharge^b^: 1979 ± 309 *vs*. 2081 ± 400 g, *p* = 0.07
Shadkam et al. [45] 2015 Single centre Iran	GA^b^: 30.87 ± 1.90 *vs*. 30.97 ± 1.94; *p* = 0.841. BW^b^: 1396.33 ± 234.55 *vs*. 1418.67 ± 328.47; *p* = 0.712	*n* = 29/30 vs. 28/30; *L. reuteri DSM 17938 vs*. Placebo (distilled water)	20 million live bacilli/kg starting on 4th day of feeding when, volume of feeds reached 40 ml/kg/day twice a day and continued until the volume of milk intake by the infant reached 120 ml/kg/day	Other outcomes: Weight at discharge^b^: (*n* = 29 *vs*. 28): 1756.55 ± 146.39 *vs*. 1747.32 ± 159.51 g; *p* = 0.821
Patole et al. [46] 2014 Single centre Australia	GA^c^: 29 (26–30; 23–32) *vs*. 28 (26–29; 23–33), BW^c^:1090 (755–1280; 466–1830) *vs*. 1025 (810–1260; 480–1770)	*n* = 79 vs. 80; *B. breve M-16V* vs Placebo (maltodextrin)	3 ×10^9^ CFU/day in two divided doses, started with first enteral feed and continued till 37 weeks CGA	Other outcomes: Discharge Weight^a^: (*n* = 77 *vs*. 76): 2590 (2184–2990; 1565–4290) *vs*. 2565 (2303–3080; 1605–5074) g; *p* = 0.539
Totsu et al. [47] 2014 Multi centre Japan	GA^b^: 28.6 ± 2.9 *vs*. 28.5 ± 3.3; *p* = NS. BW^b^: 1016 ± 289 *vs*. 998 ± 281; *p* = NS	*n* = 119/153 vs. 114/130; *B. bifidum*; *vs*. Placebo (dextrin)	2.5 × 10^9^ viable cells of *B. bifidum* per day, in 2 divided doses until weight ≥2 kg	Other outcomes: Weight at discharge^b^: 2831.8 ± 581.0 *vs*. 2876.8 ± 499.2 g; *p* = NS. Wt gain/hospital days^b^: 20.1 ± 3.7 *vs*. 20.8 ± 4.0 g/day; *p* = NS. HC at discharge^c^: 34.5 (33.8–35.5) *vs*. 34.8 (33.7–36.0) cm; *p* = NS. Increased HC/hospital days^c^: 0.10 (0.09–0.11) *vs*. 0.10 (0.09–0.12) cm; *p* = NS.
Van Niekerk et al. [48] 2014 Single centre South Africa	GA: HIV non exposed (24–28: 43% *vs*. 56%, 29–32: 53% *vs*. 40%, 33–36: 4% *vs*. 4%), BW^b^: HIV non exposed (972 ± 164); *p* = 0.12	*n* = 54 vs. 56 for HIV non- exposed group; *L. rhamnosus GG* and *B. infantis* vs Placebo: Medium Chain Triglyceride (MCT) oil	Daily *L. rhamnosus* GG (0.35×10^9^ CFU) and *B. infantis* (0.35 ×10^9^ CFU) *vs*. MCT oil (5 drops), continued till 28 days postconceptional age	Average Daily weight gain in HIV-unexposed group^d^: 13.37; ±5.99 (8.27–17.39) *vs*. 14.06; ±6.79 (9.32–18.05) g/kg, *P* = 0.61 Growth in HIV-unexposed group: Weight: At D7: 994.934 ± 164.7681 vs. 937.481 ± 154.028 g; At D14: 1021.240 ± 180.678 vs. 1004.63 ± 180.678 g; At D21: 1144.962 ± 184.580 vs. 1153.635 ± 204.550 g; At D28: 1284.67 ± 212.16 vs. 1318.958 ± 252.662 g Length: At D7: 36.673 ± 2.468 vs. 37.023 ± 2.396 cm; At D14: 37.667 ± 2.196 vs. 37.660 ± 2,124 cm; At D21: 38.36 ± 2.163 vs. 38.390 ± 2.347 cm; At D28: 39.308 ± 2.237 vs. 39.596 ± 2.351 cm Head circumference: At D7: 26.147 ± 1.393 vs. 26.365 ± 1.409 cm; At D14: 26.842 ± 1.429 vs. 27.023 ± 1.339 cm; At D21: 27.66 ± 1.503 vs. 27.853 ± 1.579 cm; At D28: 28.620 ± 1.429 vs. 28.789 ± 1.642 cm
Demirel et al. [49] 2013 Single centre Turkey	GA^b^: 29.4 ± 2.3 *vs*. 29.2 ± 2.5. BW^b^: 1164 ± 261 *vs*. 1131 ± 284	*n* = 135/138 vs. 136/140; S. *boulardii vs*. Control	5 billion CFU once daily till discharge	Other outcomes: Wt gain did not differ between the probiotic and control groups. Mean Weight At 14 days: mean [95%CI]: 1202 [1154.5–1249.5] *vs*. 1186 [1137.1–1234.9] g. At 28 days: 1369 [1314.6–1423.7] *vs*. 1378 [1323.5–1433.9] g. At 42 days: 1571 [1503.4–1639.8] *vs*. 1555 [1493.0–1617.6] g. At 56 days: 1685 [1608.9–1761.7] *vs*. 1654 [1599.3–1709.7] g
Jacobs et al. [50] PRO-PREMS Study 2013 Multi centre Australia	GA^b^: 27.9 ± 2.0 *vs*. 27.8 ± 2.0, BW^b^: 1063 ± 259 *vs*. 1048 ± 260	*n* = 548 vs. 551; *B. infantis*, (BB02300), *S. thermophilus* (TH4350) and *B. lactis* (BB12350) *vs*. Placebo (maltodextrin)	1 ×10^9^ CFU twice daily until discharge from hospital or term corrected age	Other outcomes: Weight at 28 days^b^: 1495.0 ± 401.2 *vs*. 1446.0 ± 379.2 g; *p* = 0.04. Wt at discharge^b^: 2870.5 ± 748.8 *vs*. 2864.0 ± 738.9 g; *p* = 0.89
Serce et al. [51] 2013 Single centreTurkey	GA^b^: 28.8 ± 2.2 *vs*. 28.7 ± 2.1, BW^b^: 1126 ± 232 *vs*. 1162 ± 216	*n* = 104 vs. 104; *S. boulardii vs*. Placebo (distilled water)	*S. boulardii (*10^9^ organisms) twice daily *vs*. distilled water (1 ml twice daily). Commenced with first feed and continued till discharge	Other outcomes: Wt gain (g/week)^b^: 113 ± 61 *vs*. 129 ± 65; *p* = 0.31
Al-Hosni et al. [52] 2012 Multi centre USA	GA^b^: 25.7 ± 1.4 *vs*. 25.7 ± 1.4; *p* = 0.97. BW^b^:778 ± 138 *vs*. 779 ± 126; *p* = 0.96	*n* = 50 vs. 51; *L. rhamnosus GG* and *B. infantis vs*. Control	500 million CFU each of *L. rhamnosu*s and *B. infantis* daily, started from first feed and continued until discharge or until 34 weeks PMA	Primary outcome: No difference in the percentage of infants with weight <10th percentile at 34 weeks PMA [27/47 (58%) *vs*. 28/47 (60%); *p* = 0.83]. Average daily volume of feeding (ml/kg) was lower compared to controls in first four weeks. Average daily Wt gain^b^: 14.3 ± 7.4 *vs*.11.8 ± 4.8 g; *p* = 0.06. Overall growth velocity for cases with 28 days of data^b^: 14.9 ± 6.5 *vs*. 12.6 ± 4.5 g/day; *p* = 0.05. In infants (BW 501–750 g): average daily weight gain^b^: 13.9 ± 4.7 *vs*. 10.4 ± 4.0 g; *p* = 0.02, Growth velocity^b^: 16.8 ± 4.7 *vs*. 13.1 ± 4.1 g/day; *p* = 0.01.
Chrzanowska-Liszewska et al. [53] 2012 Single centre Poland	GA(mean): 29.62 *vs*. 29.46; BW(mean): 1227.3 *vs*. 1282.5	*n* = 21 vs. 26; *L. rhamnosus* GG ATCC 53103 *vs*. Placebo (maltodextrin)	6×10^9^ CFU daily for 42 days	Other outcomes: Wt gain on discharge: No difference (*p* = 0.567, 95% CI ( − 168,305))
Sari et al. [54] 2011 Single centre Turkey	GA^b^: 29.5 ± 2.4 *vs*. 29.7 ± 2.4, BW^b^: 1231 ± 262 *vs*. 1278 ± 282	*n* = 110 vs. 111; *L. sporogenes vs*. Control	3.5 ×10^8^ CFU daily until discharged	Other outcomes: Weight gain at 14 days^b^: 3.7 ± 7.1 *vs*. 3.7 ± 6.0 g/kg/day; *p* = 0.977. Weight gain at 28 days^b^: 10.0 ± 5.1 *vs*. 10.5 ± 5.2 g/kg/day; *p* = 0.555. Weight gain at 42 days^b^: 12.6 ± 4.3 *vs*. 12.3 ± 5.0 g/kg/day; *p* = 0.769
Indrio et al. [55] 2008 Single centre Italy	GA^b^: probiotic group: 34.0 ± 1.1 *vs*. placebo: 34.0 ± 1.1. BW^b^: probiotic group:1890 ± 432 *vs*. placebo:1850 ± 342	*n* = 10 vs. 10; *L. reuteri ATCC 55730 vs*. Placebo	1×10^8^ CFU daily commenced in between day3–5 of life, continued for 30 days	Other outcomes: Weight gain per day^b^: (probiotic) 28 ± 7.0 *vs*. (placebo) 25 ± 8.1 g
Mohan et al. [56] 2008 Single centre India	GA^b^: 31.05 ± 2.31 31.27 ± 2.56, BW^b^: 1449 ± 343 *vs*. 1398 ± 331	*n* = 37 vs. 32; *B. lactis* Bb12 vs, Placebo (human milk fortifier)	*B. lactis* (2 ×10^9^CFU) *vs*. placebo. Probiotic group: day 1–3 (1.6 ×10^9^ CFU daily) and day 4 onwards (4.8 ×10^9^ CFU daily), Commenced within 24 h and continued till day 21	Primary outcomes: Weight gain in infants receiving antibiotics^b^: (1574 ± 65 *vs*. 1375 ± 74; *p* = 0.001 on day 21), No effect on weight gain in all infants^b^:(1882 ± 53 *vs*. 1836 ± 71; *p* = 0.062; on day 21), weight gain in infants not on antibiotics: (1900 ± 78 *vs*. 1941 ± 79; *p* = NS)
Stratiki et al. [57] 2007 Single centre Greece	GA^e^: 31 (27–37) *vs*. 30.5 (26–37); *p* = 0.086, BW^e^: 1500 (900–1780) *vs*.1500 (700–1900); *p* = 0.915	*n* = 41 vs. 34; *B. lactis* fortified PTF (2 × 10^7^ CFU/g formula) vs Placebo: PTF	Dose: NS; supplement started within first two days of life, continued until discharge	Other outcomes: Weight gain^e^: 28.3 (12–38) *vs*. 30(10–40) g/day; *p* = 0.144. Length gain^e^: 1.4 (0–3) *vs*. 1.5(0–3.5) cm/week; *p* = 0.271. Head growth^e^: 1.1(0.45–1.9) *vs*. 0.9(0–2) cm/week; *p* = 0.001
Bin-Nun et al. [58] 2005 Single centre Israel	GA^b^: 30 ± 3 *vs*. 29 ± 4, BW^b^: 1152 ± 262 *vs*. 1111 ± 278	*n* = 72 vs. 73; *B. infantis*, *Streptococcus (S.) thermophilus*, and *B. bifidus) vs*. Control	Daily 1.05 ×10^9^ CFU (0.35 ×10^9^ CFU B. *infantis*, 0.35 ×10^9^ CFU *S. thermophilus*, and 0.35 ×10^9^ CFU *B. bifidus* continued till 36 weeks age	Other outcomes: Weight gain: Trend toward improved total weight gain in probiotic group. Cumulative weight gain (by 6 weeks)^b^: 691 ± 208 *vs*. 594 ± 239 g; *p* = NS
Costalos et al. [59] 2003 multicenter Greece	GA^c^: 31.1 (2.5) *vs*. 31.8 (2.7), BW^c^: 1651 (470) *vs*. 1644 (348.7)	*n* = 51 vs. 36; *S. boulardii* vs. Placebo (maltodextrin)	*S. boulardii:* 10^9^ organisms twice daily; started with enteral feeds, median duration: 30 days	Primary outcome: Weight gain^c^: 163.5(17.7) *vs*. 155.8 (16.5) g/week; *p* > 0.05
Kitajima et al. [60] 1997 Single centre Japan	GA^b^: 28.3 ± 2.3 *vs*. 28.2 ± 2.1, BW^b^: 1026 ± 241 *vs*. 1026 ± 205	*n* = 45 vs. 46; *B. breve YIT4010 vs*. Placebo (distilled water)	*B. breve YIT4010* (0.5 ×10^9^CFU) started within the first 24 h of life till 28 days	Weight gain significantly greater in colonised infants between 4 and 8 weeks of life (week 4 and 8: *p* < 0.05, week 5: *p* < 0.02, week 6 and 7: *p* < 0.001), Better growth pattern till 18 months in BBG group; *p* = NS
Reuman et al. [61] 1986 Single centre USA	GA^b^: 30.6 ± 2.7 *vs*. 30.5 ± 2.8, BW^b^: 1366 ± 302 *vs*. 1377 ± 344	n = 15 vs. 15; *L. acidophilus* fortified formula *vs*. Placebo (formula)	1 ml of formula (6.8 ×10^8^ to 11 ×10^8^ organisms/ml) twice daily containing lactobacilli or placebo; duration: NS	Other outcomes: Average weight gain^b^: *n* = 7 *vs*. 7: 16 ± 5 *vs*. 15 ± 7 g/day

(For all data: results presented as probiotics vs control/ placebo groups; GA provided in weeks and BW in grams).

None of the included studies reported any adverse events.

*3Di* developmental, dimensional and diagnostic interview, *BBG* Bifidobacterium breve YIT4010, *BL* body length, *BSID* bayley’s scale of infant development, *BW* birth weight, *CFU* colony forming units, *CGA* corrected gestational age, *CI* confidence interval, *CP* cerebral palsy, *DQ* developmental quotient, *EBM* expressed breast milk, *FSIQ* full scale intelligent quotient, *GA* gestational age, *HC* head circumference, *HINE* hammersmith infant neurological examination, *HIV* human immunodeficiency virus, *IUGR* intrauterine growth restriction, *MCT* medium chain triglycerides, *MD* mean difference, *MDI* mental development index, *MSEL* mullen’s scale of early learning, *NDA* neurodevelopmental assessment, *NDI* neurodevelopmental impairment, *NEPSY-II* Developmental Neuropsychological assessment, *NS* not specified, *PCC* partial correlation coefficient, *PDI* psychomotor development index, *PMA* postmenstrual age, *PTF* preterm formula, *SD* standard deviation, *SRS* social responsiveness scale, *VABS-II* vineland adaptive behavioral scale, *VLBW* very low birth weight, *WPPSI* Wechsler preschool and primary scale of intelligence, *Wt* body weight.

^a^Median, interquartile range, range.

^b^Mean (SD).

^c^Median, interquartile range.

^d^Median, ±SD, interquartile range.

^e^Median, range.

### Characteristics of included studies

A total of twenty-seven RCTs [34–61] (*n* = 4018) reported effects of probiotics on short-term growth. Seven RCTs [62–68] (*n* = 1982) reported on neurodevelopmental outcomes, of which four [65–68] (*n* = 1417) reported on long-term growth. The results of the PROPEL trial [34, 37], Patole et al. [46, 62], Totsu et al. [47, 65], Sari et al. [54, 67] and PROPREMS trial [50, 66] were reported as two separate publications each for different outcomes.

Twenty-two RCTs were single centre [35, 36, 39, 40, 42, 43, 45, 46, 48, 49, 51, 53–58, 60–64, 67, 68], while eight were multicentre RCTs [34, 37, 38, 41, 44, 47, 50, 52, 59, 65, 66]. Primary and secondary outcomes in the included studies varied (Supplementary Table 2).

Single-strain probiotic was used in 21 RCTs: Bifidobacteria [36, 44, 46, 47, 56, 57, 60, 62, 65], Lactobacillus [34, 35, 37, 38, 45, 53–55, 61, 63, 64, 67] and Saccharomyces [42, 49, 51, 59]. Multi-strain probiotic was used in nine RCTs including one that used two Bifidobacterium strains from same genus [41], whereas eight used a combination of probiotic strains from different genera [39, 40, 43, 48, 50, 52, 58, 66, 68].

Placebo was used for comparison in 17 RCTs: maltodextrin [34, 37, 41, 44, 46, 47, 50, 53, 59, 62, 65, 66], medium chain triglyceride (MCT) oil [48], combination of MCT oil and sunflower oil [38], distilled water [45, 51, 60], human milk fortifier [56], milk formula [57, 61] and unspecified formulation [36, 55]. Remaining 13 RCTs used control/no treatment.

Four RCTs used only expressed breast milk (EBM) [43, 45, 63, 68], while 4 used pasteurized donor human milk (PDHM) along with EBM [34, 37, 39, 48, 56]. Fourteen RCTs used a combination of EBM or PDHM with infant formula [36, 40, 41, 44, 46, 47, 49–51, 54, 58, 60–62, 64–67]. Seven RCTs used formula only for feeds [35, 38, 42, 53, 55, 57, 59], while one did not specify the type of milk [52].

Thirteen RCTs assessed growth [35, 36, 38, 40–43, 48, 52, 56, 59, 65, 67] and 7 assessed long-term neurodevelopment as a primary outcome [62–68].

The 30 included RCTs had significant variation in the dosage of probiotics ranging from 2 million [40] to 10 billion colony forming units per day [47]. The duration of supplementation ranged from 21 days [56] to 6 weeks [41]. Some studies used corrected gestational age (CGA) as an endpoint [37, 46] whereas few continued the supplementation until discharge [51] or achieving weight > 2000g [47].

Probiotic and placebo group data was included from the RCT by Dilli et al. [44] and data from prebiotic/synbiotics group was excluded in the review. Similarly, Data only from the HIV-unexposed group was used from RCT by Van Niekerk et al. [48].

Growth was assessed at different time points (28 RCTs, *n* = 4311), ranging from day 14 [49, 54] to 6 weeks of age [58], to discharge [46]. The timing of assessment of long-term growth varied from 18 months [65] to 3 years of age [68].

Five RCTs reported on neurodevelopmental outcomes (motor, sensory or cognitive) [63, 65–68]. Out of them, three assessed children using Bayley Scales for Infant Development-second edition at 18–24 months [63, 67] or at three years [68] post-term. Jacobs et al. [66] assessed children at 2–5 years using Bayley-III, Movement Assessment Battery for Children and Wechsler Preschool and Primary Scale of Intelligence full scale Intelligence Quotient (IQ). Totsu et al. [65] used the Kyoto Scale of Psychological Development 2001 (equivalent to Bayley-III) at 18 months. Follow up rates varied from 48% [66] to 90% [68].

### Outcomes

The results of the systematic review including 30 RCTs (35 publications) are summarised in Table 2 and Supplementary Table 3.

**Table 2:** Overview of growth and neurodevelopment data from included studies.

Study ID	Short term growth			Long term growth			Neurodevelopmental impairment (overall)	Cognitive impairment	Cerebral palsy	Visual impairment	Hearing impairment	Behavioural issues including autism
	Wt. gain	Lt. gain	Increase in HC	Wt.	Ht.	HC						
Agrawal 2020	.	.	.	.	.	.	↔	↔	.	↔	↔	↔
Akar 2016	.	.	.	.	.	.	↔	↔	↔	↔	↔	.
Al-Hosni 2012	↑	.	.	.	.	.	.	.	.	.	.	.
Bin Nun 2005	↔	.	.	.	.	.	.	.	.	.	.	.
Chrzanowska-Liszewska 2012	↔	.	.	.	.	.	.	.	.	.	.	.
Chou 2010	.	.	.	↔	↔	↔	↔	↔	↔	↔	↔	.
Choudhury 2015	↔	.	.	.	.	.	.	.	.	.	.	.
Costalos 2003	↔	.	.	.	.	.	.	.	.	.	.	.
Cui 2019	↑	↑	↑	.	.	.	.	.	.	.	.	.
Demirel 2013	↔	.	.	.	.	.	.	.	.	.	.	.
Dilli 2015	↔	↑	↔	.	.	.	.	.	.	.	.	.
Hays 2016	↔	↔	↔	.	.	.	.	.	.	.	.	.
Indrio 2008	↔	.	.	.	.	.	.	.	.	.	.	.
Indrio 2017	↑	.	.	.	.	.	.	.	.	.	.	.
Jacobs 2013^a^	↑/ ↔	.	.	.	.	.	.	.	.	.	.	.
Jacobs 2017	.	.	.	↔	↔	↔	↔	↔	↔	↔	↓	↔
Kitajima 1997	↑	.	.	.	.	.	.	.	.	.	.	.
Mohan 2008^b^	↑/↔	.	.	.	.	.	.	.	.	.	.	.
Oshiro 2019	↑	.	.	.	.	.	.	.	.	.	.	.
Patole 2014	↔	.	.	.	.	.	.	.	.	.	.	.
Reuman 1986	↔	.	.	.	.	.	.	.	.	.	.	.
Romeo 2011	.	.	.	.	.	.	↓	.	.	.	.	.
Sari 2011	↔	.	.	.	.	.	.	.	.	.	.	.
Sari 2012	.	.	.	↔	↔	↔	↔	↔	↔	↔	↔	.
Serce 2013	↔	.	.	.	.	.	.	.	.	.	.	.
Shadkam 2015	↔	.	.	.	.	.	.	.	.	.	.	.
Shashidhar 2017	↔	.	.	.	.	.	.	.	.	.	.	.
Spreckels 2021^c^	↔	↔	↑	.	.	.	.	.	.	.	.	.
Stratiki 2007	↔	↔	↑	.	.	.	.	.	.	.	.	.
Sukanyaa 2017	↑	.	.	.	.	.	.	.	.	.	.	.
Totsu 2014	↔	↔	↔	.	.	.	.	.	.	.	.	.
Totsu 2018	.	.	.	↔	↔	↔	↔	↔	↔	.	.	.
Van Niekerk 2014	↔	↔	↔	.	.	.	.	.	.	.	.	.
Wejryd 2019^c^	↔	↔	↑	.	.	.	.	.	.	.	.	.
Xu 2016	↑	↔	↔	.	.	.	.	.	.	.	.	.

*PS* probiotic supplemented infants.

↑: significant increase in PS, ↓ : significant decrease in PS; ↔ : no significant difference between groups, ∙: not reported.

^a^Jacobs et al. Reported significant weight gain in PS at 28 days and comparable weight gain between groups at discharge.

^b^Mohan et al. reported significant increase in weight gain in PS receiving antibiotics vs controls, no difference in weight gain in all infants.

^c^Spreckels and Wejryd are two different publications from the same RCT (PROPEL).

Results of meta-analysis are presented in Supplementary Table 4.

#### Short-term growth

**Fig. 2:**
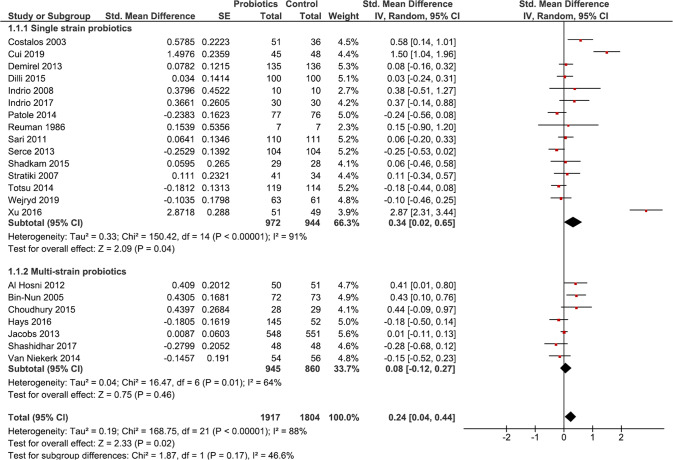
Forest plot illustrating effect of probiotics on short-term weight gain in preterm infants. Meta-analysis showed that probiotic supplementation was associated with better short-term weight gain (*p* = 0.02). Subgroup analysis showed better short-term weight gain (*p* = 0.04) in single-strain supplemented infants. This analysis was conducted using a random effects model. CI confidence interval, I^2^ heterogeneity, IV inverse variance, SE standard error.

Twenty-two RCTs [35, 37–39, 41–52, 54, 55, 57–59, 61] (*n* = 3721) reported on weight gain. Meta-analysis showed that probiotic supplemented infants had significantly better weight gain [SMD 0.24 (95%CI 0.04, 0.44); *p* = 0.02; *I*^*2*^ = 88%; CoE: low] (Fig. 2). Meta-analysis of seven studies [35, 37, 41, 42, 44, 48, 57] (*n* = 899) showed no difference in length gain between groups [SMD 0.12 (95%CI −0.13, 0.36); *p* = 0.35; *I*^*2*^ = 69%; CoE: low] (Supplementary Fig. 1). Meta-analysis of eight studies [35, 37, 41, 42, 44, 47, 48, 57] (*n* = 1132) showed no difference in head circumference gain between groups [SMD 0.09 (95%CI −0.15, 0.34); *p* = 0.46; *I*^*2*^ = 76%; CoE: low] (Supplementary Fig. 2).

#### Long-term growth

Four studies [65–68] reported on long-term growth. Meta-analysis showed no significant difference in weight [*n* = 1326; SMD −0.08 (95%CI −0.29, 0.12); p = 0.42; *I*^*2*^ = 68%; CoE: very low], length [*n* = 1325; SMD −0.03 (95%CI −0.14, 0.07); *p* = 0.53; *I*^*2*^ = 0%; CoE: very low], and head circumference between the groups [*n* = 1298; SMD −0.04 (95%CI −0.14, 0.07); *p* = 0.52; *I*^*2*^ = 0%; CoE: very low] (Supplementary Figs. 3–5).

#### Long-term neurodevelopmental outcomes

##### Cognitive outcomes

Meta-analysis of four RCTs [63, 66–68] (*n* = 1388) showed no significant difference in cognitive impairment between probiotic and control group [RR 0.98 (95%CI 0.75, 1.26); *p* = 0.85; *I*^*2*^ = 0%; CoE: very low] (Supplementary Fig. 6). Meta-analysis of five RCTs [63, 65–68] (*n* = 1507) showed no difference in mean cognitive scores between the two groups [MD 0.13 95%CI −1.41, 1.67); *p* = 0.87; *I*^*2*^ = 0%; CoE: very low] (Supplementary Fig. 7).

##### Motor outcomes

Meta-analysis of four RCTs [63, 66–68] (*n* = 1388) showed no significant difference in motor impairment between probiotic and placebo groups [RR 1.06 (95%CI 0.79, 1.41); *p* = 0.71; *I*^*2*^ = 0%; CoE: very low] (Supplementary Fig. 6). Meta-analysis of four RCTs [63, 66–68] (*n* = 1388) showed no difference in mean motor scores between the two groups [MD 1.04 (95%CI −0.43, 2.50); *p* = 0.16; *I*^*2*^ = 0%; CoE: very low] (Supplementary Fig. 7).

##### Overall neurodevelopmental, hearing, and visual impairment, CP and ASD

**Fig. 3:**
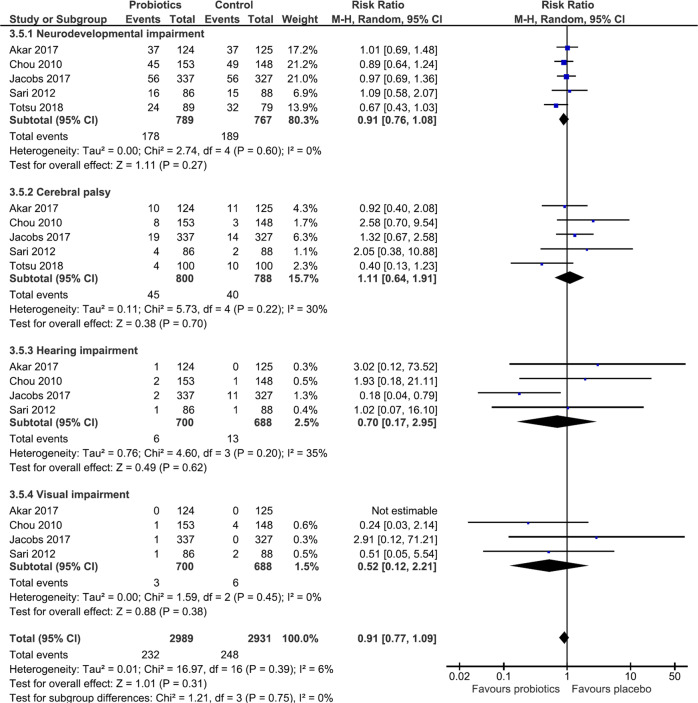
Forest plot illustrating effect of probiotics on overall neurodevelopment in preterm infants. Probiotic supplementation had no effect on overall neurodevelopmental impairment. This analysis was conducted using a random effects model. CI confidence interval, M-H Mantel-Haenszal test, I^2^ heterogeneity.

Meta-analysis of five RCTs [63, 65–68] showed no difference in neurodevelopmental impairment between groups [*n* = 1556; RR 0.91(95%CI 0.76–1.08); *p* = 0.27; *I*^*2*^ = 0%; CoE: low]. There was no difference in the incidence of CP [5 studies [63, 65–68], (*n* = 1588); RR 1.11(95%CI 0.64–1.91); *p* = 0.70; *I*^*2*^ = 30%; CoE: very low]. Meta-analysis of four RCTs [63, 66–68] (*n* = 1388) showed no difference in the incidence of hearing impairment [RR 0.7 (95%CI 0.17–2.95; *p* = 0.62; *I*^*2*^ = 35%; CoE: low] or visual impairment between groups [RR 0.52 (95%CI 0.12–2.21); *p* = 0.38; *I*^*2*^ = 0%; CoE: very low]. Meta-analysis of effect of probiotics on overall neurodevelopmental impairment, CP, hearing impairment and visual impairment showed no difference between groups (Fig. 3). None of the included studies reported on ASD.

#### Subgroup analysis: (i) Gestation < 28 weeks or birthweight < 1000g

Three RCTs were eligible [37, 48, 52]. Meta-analysis of three RCTs (*n* = 335) showed no difference in short term weight gain [SMD 0.05 (95%CI −0.29, 0.38); *p* = 0.79; *I*^*2*^ = 60%] (Supplementary Fig. 8). Meta-analysis of two RCTs [37, 48] (*n* = 234) showed no difference in short term length gain [SMD −0.10 (95%CI −0.36, 0.15); *p* = 0.43; *I*^2^ = 0%] or head circumference gain [SMD 0.04 (95%CI −0.24, 0.32); *p* = 0.77; *I*^2^ = 17% (Supplementary Figs. 9–10).

#### (ii) Single-strain and multi-strain probiotics

Meta-analysis of 15 RCTs [35, 37, 38, 42, 44–47, 49, 51, 54, 55, 57, 59, 61] using single strain probiotic (*n* = 1916) showed better short-term weight gain [SMD 0.34 (95%CI 0.02, 0.65); *p* = 0.04; *I*^*2*^ = 91%]. Meta-analysis of 7 RCTs [39, 41, 43, 48, 50, 52, 58] using multi-strain probiotics (*n* = 1805) showed no difference in short-term weight gain [SMD 0.08 (95%CI −0.12, 0.27); *p* = 0.46; *I*^*2*^ = 64%] (Fig. 2).

### Summary of outcomes from studies not included in meta-analysis

Three RCTs [36, 56, 60] (*n* = 195) reported significantly better short-term weight gain, Romeo et al. [64] (*n* = 249) reported improved neurodevelopment in the probiotic group at 12 months CGA (Table 1).

Risk of bias assessment for studies reporting on growth showed that 15.6% had high risk, 18.8% had some concerns, whereas 65.6% had low risk. Five RCTs seemed to have overall high ROB for assessing growth as outcome, whereas six studies had some concerns. A total of 21 studies were assessed as having low ROB (Supplementary Fig. 11). Risk assessment for studies reporting long-term neurodevelopmental outcomes showed 57.1% had high ROB, 14.3% had some concerns and 28.6% had low ROB (Supplementary Fig. 11). Missing data on long term neurodevelopmental outcomes of participants in the RCTs was a major reason for ‘high ROB.’

Funnel plot for comparison of probiotics for short term weight gain appeared to have no asymmetry (Supplementary Fig. 12) but Egger’s test (*p* = 0.0217) and Begg’s test (*p* = 0.0112) result showed that publication bias was likely. Publication bias could not be assessed for other outcomes as there were < 10 studies in the meta-analyses.

### Summary of findings (GRADE evidence)

The CoE for short-term growth was deemed ‘low’. The CoE for long-term growth and neurodevelopmental outcomes was deemed ‘low to very low’ in view of significant statistical heterogeneity, differences in timing of assessments of outcomes and definitions, wide CIs and overall moderate to high ROB in the included studies (Table 3).

**Table 3:** Summary of findings for pooled data as per GRADE guidelines.

Certainty assessment							No of patients		Effect		Certainty
No of studies	Study design	Risk of bias	Inconsistency	Indirectness	Imprecision	Other considerations	probiotic	placebo	Relative (95% CI)	Absolute (95% CI)	
22	RCTs	Serious^a^	Serious^b^	Serious^c^	Serious^c^	None	1917	1804	-	SMD 0.24 higher (0.04 higher to 0.44 higher)	⊕⊕○○ Low
7	RCTs	Not serious	Serious^b^	Serious^c^	Serious^d,e^	None	499	400	-	SMD 0.12 higher (0.13 lower to 0.36 higher)	⊕⊕○○ Low
8	RCTs	Not serious	Serious^b^	Serious^c^	Serious^d,e^	None	618	514	-	SMD 0.09 higher (0.15 lower to 0.34 higher)	⊕⊕○○ Low
4	RCTs	Very serious^f^	Serious^c^	Serious^c^	Serious^d,e^	All plausible residual confounding would reduce the demonstrated effect	666	660	-	SMD 0.08 lower (0.29 lower to 0.12 higher)	⊕○○○ Very Low
4	RCTs	Very serious^f^	Serious^c^	Serious^c^	Serious^d,e^	All plausible residual confounding would suggest spurious effect, while no effect was observed	665	660	-	SMD 0.03 lower (0.14 lower to 0.07 higher)	⊕○○○ Very Low
4	RCTs	Very serious^f^	Serious^c^	Serious^c^	Serious^d,e^	All plausible residual confounding would reduce the demonstrated effect	648	650	-	SMD 0.04 lower (0.14 lower to 0.07 higher)	⊕○○○ Very Low
5	RCTs	Very serious^f^	Not serious	Serious^c^	Serious^e^	All plausible residual confounding would reduce the demonstrated effect	178/789 (22.6%)	189/767 (24.6%)	RR 0.91 (0.76 to 1.08)	22 fewer per 1,000 (from 59 fewer to 20 more)	⊕⊕○○ Low
5	RCTs	Very serious^f^	Not serious	Serious^c^	Serious^d^	All plausible residual confounding would reduce the demonstrated effect	45/800 (5.6%)	40/788 (5.1%)	RR 1.11 (0.64 to 1.91)	6 more per 1,000 (from 18 fewer to 46 more)	⊕○○○ Very Low
3	RCTs	Serious^f^	Not serious	Serious^c,d^	Very serious^d^	All plausible residual confounding would reduce the demonstrated effect	6/700 (0.9%)	13/688 (1.9%)	RR 0.70 (0.17 to 2.95)	6 fewer per 1,000 (from 16 fewer to 37 more)	⊕○○○ Very Low
3	RCTs	Serious^f^	Not serious	Serious^c^	Very serious^d,e^	All plausible residual confounding would reduce the demonstrated effect	3/700 (0.4%)	6/688 (0.9%)	RR 0.52 (0.12 to 2.21)	4 fewer per 1,000 (from 8 fewer to 11 more)	⊕○○○ Very Low
4	RCTs	Serious^f^	Serious^c^	Serious^c^	Serious^d^	All plausible residual confounding would reduce the demonstrated effect	99/700 (14.1%)	100/688 (14.5%)	RR 0.98 (0.75 to 1.26)	3 fewer per 1,000 (from 36 fewer to 38 more)	⊕○○○ Very Low
4	RCTs	Serious^f^	Serious^c^	Serious^c^	Serious^d^	All plausible residual confounding would reduce the demonstrated effect	83/700 (11.9%)	77/688 (11.2%)	RR 1.06 (0.79 to 1.41)	7 more per 1,000 (from 24 fewer to 46 more)	⊕○○○ Very Low

*CI* confidence interval, *RR* risk ratio, *SMD* standardized mean difference.

^a^Egger’s test showed publication bias.

^b^Significant statistical heterogeneity.

^c^Difference in outcome time frames and definition of outcomes.

^d^Wide confidence intervals.

^e^Inadequate sample size.

^f^Overall moderate to high risk of bias.

## Discussion

Our systematic review found that preterm infants supplemented with probiotics had better short-term weight gain, but the size of benefit (SMD 0.24) was small. SMD values of 0.2–0.5 are considered small, 0.5–0.8 medium and > 0.8 are considered large [69]. However, probiotics had no significant effect on linear and head growth, as well as long-term growth, and neurodevelopment, CP, hearing, or visual impairment. Subgroup analyses showed improved weight gain with single strain, but not with multi-strain probiotics. It was reassuring that none of the included studies noted adverse effects related to probiotics.

In contrast to our findings, the systematic review by Sun et al. [70] showed lower short-term weight gain (primary outcome) in probiotic supplemented VLBW infants, however, this was statistically non-significant. They suggested that variable timing and methods of weight measurement may explain the lack of benefit in the pooled results. Furthermore, their subgroup analysis showed that the type of feeding may influence the effect of probiotics on weight gain. Probiotic supplemented infants fed either breast milk or formula had better weight gain [(MD: 2.2 g (95% CI: 0.08 to 4.48 g; *p* < 0.05)] compared to those fed only formula [(MD: −0.89 g (95% CI: −3.97 to 2.18; *p* = 0.57)].

Totsu et al. reported significantly better short-term weight gain in the probiotic group in their pilot trial [47]. However, their cluster RCT showed no difference in body weight, length, or head circumference at 18 months between probiotic and placebo groups [71]. The improved short-term weight gain following probiotic supplementation could relate to increased absorption of key nutrients in the early postnatal period and reduced gut dysbiosis and nutrient malabsorption by strengthening the gut barrier [72–75].

The lack of effect on short-term linear or head growth or overall long-term growth could be explained by the interplay between several variables including the degree and complications of prematurity (e.g. LOS, NEC, chronic lung disease), type of feeding, postnatal exposure to steroids, and differences in strategies for neonatal enteral and parenteral nutrition, and post discharge nutrition [70, 76–79].

Similar to our findings, the recent Cochrane systematic review showed that probiotics may have little or no effect on severe neurodevelopmental impairment (RR: 1.03; 95% CI: 0.84 to 1.26; five RCTs, *n* = 1518 infants; CoE: low) [20]. There were no differences in CP, visual or hearing impairment and cognitive performance. In their systematic review, Upadhyay et al. reported improvement in mean motor scores (*p* = 0.07) in probiotic group, but suggested caution given the overall high risk of bias [25]. Romeo et al. reported normal range of optimal scores on Hammersmith Infant Neurological Examination at 12 months in the probiotic group [64]. The authors attributed these findings to reduced Candida infections and gut colonisation with probiotic bacteria [64]. Totsu et al. reported improved developmental quotient scores following B. *bifidum OLB 6378* supplementation in neonatal period in female infants at 18 months with reduced rates of CP [65].

In addition to their anti-neuroinflammatory effects, probiotics may influence neurodevelopment through short-chain fatty acids (SCFA) production. SCFAs play an important role in ‘gut-brain’ axis by regulating central nervous system processes (e.g., cell-cell interaction, neurotransmitter synthesis and release, microglia activation, mitochondrial function, and gene expression) [80–83]. SCFAs promote neurosphere growth from human neural stem cells and differentiation of embryonic stem cells into neural cells [84].

Upadhyay et al. [25] reported significantly lower risk of hearing impairment (RR 0.25; 95% CI: 0.07, 0.88) (*n* = 838; *I*^*2*^ = 15.2%; *p* = 0.03) in a subgroup where multi-strain probiotics were commenced within first week of life. The ProPrems RCT also reported reduced hearing impairment in the probiotic group. Reduced cochlear and outer hair cell injury was pointed out as a possible mechanism for this benefit of probiotics [66].

The lack of effect of probiotics on neurodevelopment in our review could relate to variations in probiotic strain, dose and duration in different studies and ages and methods of neuro-developmental assessment. Considering only seven RCTs reported neurodevelopmental data, inadequate sample size is a concern. Furthermore, it is difficult to comment on the role played parental education and socio-economic status, factors known to influence neurodevelopmental outcomes [85, 86]. Our subgroup analyses (gestation < 28 week and single *vs*. multistrain probiotics) were limited due to lack of suitable data from included studies. Furthermore, individual participant details would be needed for a detailed gestational age wise analysis, which is beyond the scope of our review. One of the major limitations of our study includes small sample size and number of included studies for comparisons of long-term growth and neurodevelopmental outcomes. For example, hearing, and visual impairment had only four studies (*n* = 1388) to compare.

Despite its limitations, we believe that our comprehensive systematic review with robust methodology focussing specifically on growth and neurodevelopment adds useful data to guide research and clinical practice in this field. Emerging evidence supports the importance of early postnatal enteral nutrient intake in brain development and maturation, and role of gut-microbiota in long-term neurodevelopment and neuropsychiatric disorders [18, 22, 87–90]. Given these data, and our results, adequately powered well designed RCTs are justified to assess the long-term effects of probiotics in preterm infants. Such RCTs should stratify infants based on gestational age (<28w, 29–32w and > 32w). Adequate information needs to be provided to parents regarding the controversies about the short-term benefits such as reduction in NEC, mortality and LOS to enable them to consent for participating in a placebo-controlled trial. In units where there is no equipoise, routine probiotic supplementation could be considered if a suitable product is available. Well-designed and adequately powered prospective observational studies will be helpful to assess the effects of probiotic supplementation on long-term growth and neurodevelopment in preterm infants in such real-life circumstances.

## Conclusions

This systematic review and meta-analysis showed that preterm infants supplemented with probiotics had better weight gain during initial hospitalisation. However, probiotics had no significant effect on linear and head growth, as well as long-term growth, and neurodevelopment.

## Supplementary information

Supplemental tables and figures

## Data Availability

Data described in the manuscript, code book, and analytic code will be made available upon reasonable request sent via email to gayatri.jape@health.wa.gov.au.
