# Supplementary material for: Growth and neuro-developmental outcomes of probiotic supplemented preterm infants—a systematic review and meta-analysis
Source: Eur J Clin Nutr. 2023 Feb 14;77(9):855–71. doi: 10.1038/s41430-023-01270-2 (PMC10473962; doi:10.1038/s41430-023-01270-2)

SUPPLEMENTAL DATA

Supplemental tables

Supplemental Table 1: Characteristics of included studies on effects of probiotics on growth and neurodevelopment in preterm infants

Supplemental table 2: Reported outcomes of included studies on effects of probiotics on growth and neurodevelopment in preterm infants

Supplemental Table 3: Overview of other clinical outcomes from included studies

Supplemental Table 4: Summary of results of meta-analysis

Supplemental figures

Supplemental Figure 1: Forest plot illustrating effect of probiotics on short-term length gain in preterm infants

Supplemental Figure 2: Forest plot illustrating effect of probiotics on increase in short-term head circumference in preterm infants

Supplemental Figure 3: Forest plot illustrating effect of probiotics on long-term weight gain in preterm infants

Supplemental Figure 4: Forest plot illustrating effect of probiotics on long-term linear growth in preterm infants.

Supplemental Figure 5: Forest plot illustrating effect of probiotics on long-term head growth in preterm infants.

Supplemental Figure 6: Forest plot illustrating effect of probiotics on cognitive and motor impairment outcomes in preterm infants.

Supplemental Figure 7: Forest plot illustrating effect of probiotics on mean cognitive and motor scores in preterm infants.

Supplemental Figure 8: Forest plot illustrating effect of probiotics on increase in short-term weight gain in <28 weeks preterm infants.

Supplemental Figure 9: Forest plot illustrating effect of probiotics on increase in short-term length gain in <28 weeks preterm infants.

Supplemental Figure 10: Forest plot illustrating effect of probiotics on increase in short-term head circumference gain in <28 weeks preterm infants.

Supplemental Figure 11: Quality assessment of included studies using Cochrane risk of bias (ROB-2) tool.

Supplemental Figure 12: Funnel plot illustrating publication bias.

Supplemental Table 1: Characteristics of included studies on effects of probiotics on growth and neurodevelopment in preterm infants

**Study ID**	**Study characteristics**
**Studies reporting on neurodevelopment only**	
**Agrawal et al**<br>2020<br>Single centre<br>Australia<br>Follow up of original study by Patole et al[33]	**Participants: GA$^{\$}$**: 28.6 (25.7–30.7; 23.4–32.1) *vs.* 27.7 (26.1–29.1; 23.6–31.7) wk. **BW$^{\$}$**: 1055 (775–1315; 466–1535) *vs.* 960 (810–1180; 540–1735) g<br>**Intervention and Comparison: Probiotic:** n=36/79; *Bifidobacterium (B.) breve M-16 V vs.* **Placebo:** n=31/80 dextrin<br>**Dose and duration:** 3 billion CFU per day till 37 weeks CGA<br>**Type of milk**: EBM/PDHM/PTF. **Type of delivery: CS:** 75% *vs.* 65%<br>**Outcomes: Primary neurodevelopmental:** Continuous Early Learning Composite Measure from the Mullen's Scale of Early Learning (MSEL), **Secondary:** Any neurodevelopmental disability as identified by the Developmental, Dimensional and Diagnostic Interview (3Di), **Tertiary:** Other measures using a Developmental Neuropsychological assessment (NEPSY-II), parental questionnaires (Children's Communication checklist, Social Responsiveness Scale (SRS) and Vineland Adaptive Behavioral Scale or VABS-II)<br>**Neurodevelopmental assessment (NDA) tools**: as specified above; **Age at assessment:** 3-5 years age
**Akar et al**<br>2016<br>Single centre<br>Turkey	**Participants: GA$^{\#}$**: 28.9±2.1 *vs.* 28.6±2.5; p=0.28. **BW$^{\#}$**: 1138±257 *vs.* 1142±267; p=0.89<br>**Intervention and Comparison: Probiotic:** n=124/200; *Lactobacillus (L.) reuteri* vs **Control:** n=125/200<br>**Dose and duration:** 100 million organisms once daily from starting enteral feeds till discharge<br>**Type of milk**: EBM. **Type of delivery: CS:** 84% *vs.* 78%<br>**Outcomes: Primary neurodevelopmental:** Cerebral palsy, Mental Development Index on BSID-II (MDI)< 70, Psychomotor Development Index on BSID-II (PDI< 70), bilateral deafness or blindness<br>**NDA tool**: Bayley-II; **Age at assessment:** 18-24 months corrected gestational age (CGA)<br>**Secondary:** BPD, LOS, NEC stage 2, IVH grade 3-4, severe ROP
**Romeo et al**<br>2011<br>Single centre<br>Italy	**Participants: GA$^{\#}$**: 33.8±1.8 *vs.* 33.3±1.6 *vs.* 33.3±2.1; p=NS. **BW$^{\#}$**: 1998.7±439 *vs.* 1940.7±590 *vs.* 1945.7±465; p=NS<br>**Intervention and Comparison: Probiotic:** n=83 (*L. reuteri ATCC 55730*) +83 (*L. rhamnosus ATCC 53103*) vs **Control**: n=83<br>**Dose and duration:** from 72 hrs. of age till 6 weeks/discharge<br>**Type of milk**: EBM/ formula. **Type of delivery: CS:** 94% *vs.* 86% *vs.* 93%<br>**Outcomes: Primary:** enteric fungal colonization<br>**Other outcomes:** Neurological outcome at 12 months corrected age, days of hospital stay, fungal sepsis<br>**NDA tool:** Hammersmith Infant Neurological Examination (HINE**); Age at assessment:** 12 months CGA
**Studies reporting on both *neurodevelopment and long-term growth***	
**Totsu et al**<br>2018<br>Multi-centre<br>Japan<br>Follow up of original study by Totsu et al	**Participants: GA$^{\#}$**: 28.7±3.1 *vs.* 28.4±3.0 p=0.568. **BW$^{\#}$**: 1036±289 *vs.* 994±283 p=0.297<br>**Intervention and Comparison: Probiotic:** n=102/ 153; *Bifidobacterium (B.) bifidum OLB 6378 vs.* **Placebo** (Dextrin): n=105/130<br>**Dose and duration:** 5 x 10^9 CFU commenced within 48 hours after birth and administered twice daily until the infant's weight reached 2000g.<br>**Type of Milk:** EBM/ formula. **Type of delivery: CS:** 47 (46%) *vs.* 85 (81%); p<0.001<br>**Outcomes: Primary:** TFEF (100 ml/kg/day), body weight and head circumference at discharge **Neurodevelopmental outcomes:** Developmental Quotient (DQ) at 18 months corrected age<br>**NDA tool:** Kyoto Scale of Psychological Development 2001 (correlates with Bayley-II); **Age at assessment:** 18 months<br>**Other outcomes:** NEC and LOS; physical development at 18 months of age and intestinal microbiota colonisation
**Jacobs et al**<br>2017<br>**PRO-PREMS** Study<br>Multi-centre<br>Australia, New Zealand<br>Follow up of original study by Jacobs et al	**Participants: GA$^{\#}$**: 27.6±2.0 vs. 27.6±1.9 wk. **BW$^{\#}$**: 1042±267 vs. 1027±261 g<br>**Intervention and Comparison: Probiotic:** n=373/548; *B. infantis, Streptococcus thermophilus and B. lactis vs.* **Placebo** (maltodextrin): n=362/551<br>**Dose and duration:** 1 × 10^9 organisms administered from birth until discharge home or term corrected age, whichever was sooner.<br>**Type of Milk**: Ebm/ formula. **Type of delivery**: CS: 242(64.9%) vs. 253(69.9%)<br>**Primary neurodevelopmental outcome**: **Survival without neurosensory impairment at 2 years corrected age**, **Moderate/severe cerebral palsy** (Gross Motor Function Classification System score 2–5), **motor impairment** (Bayley-III Motor Composite Scale <–2SD or Movement Assessment

	Battery for Children <15th centile if >42 months' CGA), **cognitive impairment** (Bayley-III Composite Cognitive or Language Scales <–2SD or Wechsler Preschool and Primary Scale of Intelligence Full Scale Intelligence Quotient <–2SD if >42 months' CGA), **blindness** or **deafness**<br>**NDA tool:** Bayley-III; **age at assessment:** 2-5 years CGA<br>**Secondary outcomes:** Growth at mean age of 30 months
**Sari et al**<br>2012<br>Single centre<br>Turkey<br>Follow up of original study by Sari et al	**Participants: GA$^{\#}$**: 29.7±2.5 vs. 29.8±2.3 wk; p=0.648 **BW$^{\#}$**: 1241±264 vs. 1278±273 g; p=0.380<br>**Intervention and Comparison: Probiotic:** n= 86/110; *L. sporogenes* 0.35 x 10^9 CFU/ day *vs.* **Controls:** n=88/111, **Dose and duration:** Starting with the first feed continued until discharge<br>**Type of milk**: EBM, formula. **Type of delivery: CS:** 67.3% *vs.* 75.7%<br>**Primary outcome**: Growth and neurodevelopment outcomes at 18 to 22 months' corrected age<br>**NDA tool:** BSID-II; **Age at assessment:** 18-22 months CGA<br>**Other outcomes:** NEC ≥ stage 2, IVH grade 3-4, cystic PVL, ROP, CLD, LOS, duration of hospital stay
**Chou et al**<br>2010<br>Single centre<br>Taiwan	**Participants: GA$^{\#}$**: 28.5±2.3 vs. 28.5±2.3 wk; p=.90 **BW$^{\#}$**: 1103.6±232.4 vs. 1097.2±231.4 g; p=0.80<br>**Intervention and Comparison: Probiotic:** n=153/180; *Infloran* 125 mg/kg/dose with *L. acidophilus* 1 billion CFU + *B. infantis* 1 billion CFU *vs.* **Control:** n=148/187, **Dose and duration:** administered with starting enteral feeds and continued till discharge<br>**Type of milk**: EBM. **Type of delivery: CS:** 57.5% *vs.* 53.3%<br>**Outcomes of interest: Primary: Death/ neurodevelopmental impairment (NDI), NDI:** BSID-II MDI< 70, BSID-II PDI< 70, bilateral blindness, deafness needing amplification (> 55db), moderate-severe CP<br>**NDA tool:** Bayley-II; **Age at assessment:** 3 yrs corrected age<br>**Secondary: Growth** (at 3 yrs of age), NEC stage II, IVH grade 3-4, PVL, BPD, severe ROP, LOS
**Studies reporting on *growth only***	
**Spreckels et al**<br>**PROPEL** trial<br>2021<br>Multi centre<br>Sweden	**Participants: GA$^{\#}$**: 25.5±1.2 *vs.* 25.6±1.2 wk; p=0.75. **BW$^{\#}$**: 724±131 *vs.* 754±143 g; p=0.25<br>**Intervention and Comparison: Probiotic**: n=68/72: *L. reuteri DSM 17938 vs.* **Placebo**: n=66/69 Maltodextrin<br>**Dose and duration:** 1.25 x 10^8 CFU/day starting from day1-3 upto 36 weeks PMA<br>**Type of milk:** EBM/ PDM. **Type of delivery: CS:** 75% *vs.* 58%<br>**Outcomes of interest: Primary:** colonization by supplemented probiotic<br>**Other:** TFEF (150mls/kg/day), NEC stage ≥2, LOS, BPD, ROP, IVH, PVL, growth parameters: weight, length and HC at birth, 2 and 4 weeks of age
**Cui et al**<br>2019<br>Single centre<br>China	**Participants: GA$^{\#}$**: 32.85±1.39 *vs.* 32.56±1.41 wk; p=0.3206. **BW$^{\#}$**: 1682±109.03 *vs.* 1714±127.11 g; p=0.1984<br>**Intervention and Comparison: Probiotic**: n=45/57: *L. reuteri DSM 17938 vs.* **Control**: n=48/57<br>**Dose and duration:** 1×10^8 CFU (5 drops) once daily, start with first feed until hospital discharge. Minimum duration: 7 days<br>**Type of milk:** preterm formula (PTF). **Type of delivery:** NS<br>**Outcomes of interest: Primary:** feeding tolerance (TFEF), number of reflux episodes and growth (body weight/Wt, body length/ BL, and head circumference/ HC)<br>**Other:** infection prevention (incidences of LOS and NEC)
**Oshiro et al**<br>2019<br>Single centre<br>Japan	**Participants: GA$^{\#}$**: 28.1±3.1 *vs.* 28.2±3.3 wk, **BW$^{\#}$**: 1049±302 *vs.* 1002±289 g.<br>**Intervention and Comparison: Probiotic:** *B. breve*, n=17; *vs.* **Placebo:** NS**,** n=18<br>**Dose and duration:** 2.5 × 10^8 CFU once a day till discharge.<br>**Type of milk:** EBM/ formula. **Type of delivery:** CS: 14 (82.3%) *vs.* 15 (83.3%)<br>**Outcomes of interest: Primary:** body weight gain and the composite of the measured faecal and plasma outcomes during 8 weeks postpartum<br>**Secondary:** NEC, sepsis.
**Wejryd et al**	**Participants: GA$^{\#}$**: 25.5±1.2 *vs.* 25.5±1.3 wk; p=0.95. **BW$^{\#}$**: 731±129 *vs.* 740±148 g; p=0.71

**PROPEL** trial<br>2019<br>Multi Centre<br>Sweden	**Intervention and Comparison: Probiotic:** n=68; *L. reuteri DSM 17938* ***vs.*** **Placebo**: n=66; maltodextrin<br>**Dose and duration:** Daily *L. reuteri*; 1.25 x 10^8 bacteria (0.2 mL drops) started within three days after birth until 36 weeks<br>**Type of milk**: EBM/PDM. **Type of delivery: CS:** 50 (74%) *vs.* 37 (56%)<br>**Outcomes of interest: Primary:** feeding tolerance: TFEF (≥150 mL/kg/day), days of interrupted feeding due to vomiting, distended abdomen or clinically suspected NEC<br>**Secondary:** growth rate (Wt, BL and HC) and severe morbidity
**Indrio et al**<br>2017<br>Multi centre<br>Italy	**Participants: GA**[#]: 30.2±1.2 *vs.* 30.1±1.2 wk, **BW**[#]: 1471.5±455.1 *vs.* 1406.6±536.4 g<br>**Intervention and Comparison: Probiotic:** n= 30, *L. reuteri DSM 17938 vs.* **Placebo:** n= 30, mixture of sunflower oil and MCT oil<br>**Dose and duration:** once a day at a dose of 1×10^8 CFU until 30 days of life.<br>**Type of milk:** Formula. **Type of delivery:** CS: 26 (86.6%) *vs.* 25 (83.3%)<br>**Outcomes of interest: Primary:** feeding tolerance based on cytokine fecal profile, clinical parameters (TFEF, Duration of hospitalization, Duration of antibiotic treatment, Time to regain birth weight, weight at end of study, stool frequency) and ultrasound measurement.<br>**Secondary:** Cost of supplementation calculated based on duration of hospitalization.
**Shashidhar et al**<br>2017<br>Single centre<br>India	**Participants: GA**[#]: 31.2±2.1 *vs.* 31±2.1 wk, **BW**[#]: 1256±185 *vs.* 1190±208 g<br>**Intervention and Comparison: Probiotic:** n= 48/52, *L. acidophilus, L. rhamnosus, B. longum and Saccharomyces (S.) boulardii*; *vs.* **Control:** n= 48/52.<br>**Dose and duration:** once a day at a dose of 1.25×10^9 CFU until discharge.<br>**Type of milk:** EBM/ PDHM. **Type of delivery:** CS: 27 (51.9%) *vs.* 38 (73%)<br>**Outcomes of interest: Primary:** time taken to reach full enteral feeds.<br>**Secondary:** episodes of feed intolerance, incidence of NEC ≥stage 2, duration of hospital stay, days on TPN, weight gain and mortality.
**Sukanyaa S et al**<br>2017<br>Single centre<br>India	**Participants:** VLBW infants with **BW** ≤ 1,500 g, **GA** <34 wk<br>**Intervention and Comparison: Probiotic:** *L. acidophilus, B. infantis, S. boulardi vs.* **Control**: EBM only;<br>**N:** 49 randomized, of which 4 excluded. Analysed 23 *vs.* 22<br>**Dose and duration:** Half sachet (>1 million CFU) twice daily diluted with EBM, duration: NS<br>**Type of milk:** EBM/ PTF. **Type of delivery:** CS: NS<br>**Primary outcomes:** Weight gain pattern during hospital stay and up to one month after discharge<br>**Other outcomes:** Duration of hospital stay
**Hays et al**<br>2016<br>Multi-centre<br>France	**Participants: GA**[@]: 29.0 (28.1; 30.1) *vs.* 29.4 (27.9; 30.6) weeks, **BW**[@]: 1170 (1000; 1320) *vs.* 1170 (1055; 1370) g<br>**Intervention and Comparison: Probiotic:** total N=145/147; Group 1, n=50 (*B. lactis*); Group 2, n=49 (*B. longum*); Group 3, n=48 (*B. lactis* + *B. longum*) *vs.* **Placebo:** n=52 (maltodextrin). Analysed 145 *vs.* 52<br>**Dosage:** 10^9 CFU/day, 1 capsule daily containing either probiotics + maltodextrin or maltodextrin alone<br>**Duration:** Started before end of first week of life, and continued for four (if birth GA <29 weeks) or six (if birth GA <28 weeks) weeks<br>**Type of milk**: fortified EBM/ PDHM/ PTF. **Type of delivery: CS:** 79.3% vs 75%<br>**Primary outcome**: short-term postnatal growth and body composition<br>**Secondary outcomes:** safety
**Xu et al**<br>2016<br>Single centre<br>China	**Participants: GA**[#]: 33±0.72 *vs.* 33±1.04 wk, **BW**[#]: 1947±54 *vs.* 1957±51 g<br>**Intervention and Comparison: Probiotic:** n=51/63; S. *boulardii CNCM I-745 vs.* **Control**: n=49/62<br>**Dose and duration:** 10^9 CFU/kg of *S. boulardii CNCM I-745*, administered twice daily *vs.* Control. Supplement ceased at day 28 or at hospital discharge Minimal duration: 7 days

	**Type of milk:** PTF exclusively. **Type of delivery:** NS<br>**Primary Outcome:** weight gain (g/kg/day) and linear growth (cm/week)<br>**Secondary outcome:** days of PN needed to reach full enteral feeding, maximal enteral feeding volume tolerated (mL/kg/day), and hospital stay
**Choudhury et al**<br>2015<br>Single centre<br>Bangladesh	**Participants: GA#**: 31.9± 1.32 *vs.* 32.04±1.26 wk; p=0.76, **BW**: 1000 - <1800 g. BW#: NS<br>**Intervention and Comparison: Probiotic:** n=28/30; TS6 Probiotic (Eight viable strains mixture of *Lactobacillus* and *Bifidobacterium* (20 billion/2 gram) *vs.* **Control:** n=29/35<br>**Dose and duration:** Starting dose 1.65 billion CFU and increased to 3.3 billion CFU when feed volume reached 2ml/feed, continued till attainment of full enteral feed<br>**Type of milk**: EBM. **Type of delivery: CS:** 78.6% *vs.* 82.7%<br>**Primary outcome**: feed tolerance, postnatal weight gain and duration of hospital stay<br>**Secondary outcomes:** None
**Dilli et al**<br>2015<br>Multi centre<br>Turkey	**Participants:** Probiotic# (GA:28.8±1.9 wk; BW: 1236±212 g) *vs.* Prebiotic# (GA: 29.0±1.7 wk; BW: 1229±246 g) *vs.* Synbiotic# (GA: 28.9±1.9 wk; BW: 1205±240 g) *vs.* Placebo# (GA: 28.2±2.2 wk; BW: 1147±271 g)<br>**Intervention, Comparison and Dosage: Probiotic:** n=100 (Daily *B. lactis*, 5 x 10^9 CFU) *vs.* **Prebiotic**: n=100 (inulin, 900 mg) *vs.* **Synbiotic** n=100 (*B. lactis*, 5 x 10^9 CFU + 30 mg inulin) vs **Placebo:** n=100 (maltodextrin)<br>For Meta-analysis only probiotics (n=100) vs. Placebo (n=100) included<br>**Duration:** until discharge or death (maximum of 8 weeks, whichever came first)<br>**Type of milk**: EBM/ formula. **Type of delivery: CS:** 35 (35%) *vs.* 37 (37%) *vs.* 29 (29%) *vs.* 37 (37%)<br>**Outcomes of interest: Primary:** NEC<br>**Secondary:** Growth, TFEF, LOS, length of NICU stay, and mortality
**Shadkam et al**<br>2015<br>Single centre<br>Iran	**Participants: GA#**: 30.87±1.90 *vs.* 30.97±1.94 wk; p=0.841. **BW#**: 1396.33±234.55 *vs.* 1418.67±328.47g; p=0.712<br>**Intervention and Comparison: Probiotic:** n=29/30; *L. reuteri DSM 17938*; *vs.* **Placebo:** n=28/30; distilled water.<br>**Dose and duration:** a dose of 20 million live bacilli/kg administered starting on 4th day of feeding when, volume of feeds reached 40 ml/kg/day twice a day and continued until the volume of milk intake by the infant reached 120 ml/kg per day.<br>**Type of milk:** EBM. **Type of delivery:** NS<br>**Outcomes of interest: Primary:** TFEF, NEC<br>**Secondary:** Weight at discharge, supplementary feeding time, jaundice, sepsis, mortality.
**Patole et al**<br>2014<br>Single centre<br>Australia	**Participants: GA@**: 29 (26–30; 23–32) *vs.* 28 (26–29; 23–33) wk. **BW@**:1090 (755–1280; 466–1830) *vs.* 1025 (810–1260; 480–1770) g<br>**Intervention and Comparison: Probiotic:** n=79 (*B. breve M-16V*) vs **Placebo:** n=80 (maltodextrin). Analysed 77 vs. 76 for growth outcomes.<br>**Dose and duration:** 3 x 10^9 CFU/day given in two divided doses, started with first enteral feed and continued till 37 weeks CGA<br>**Type of milk**: EBM/ PDHM/ PTF. **Type of delivery: CS:** 58 (75%) *vs.* 49 (65%)<br>**Outcomes of interest: Primary:** colonization with *B. breve M-16V*<br>**Secondary:** Bifidogenic effect (elevation of total bifidobacteria in stools); incidence of NEC (≥Stage II), and all-cause mortality; TFEF (150 ml/kg/day) and blood culture positive LOS
**Totsu et al**<br>2014<br>Multi centre<br>Japan	**Participants: GA#**: 28.6±2.9 *vs.* 28.5±3.3 wk; p=NS. **BW#**: 1016±289 *vs.* 998±281 g; p=NS<br>**Intervention and Comparison: Probiotic:** n=119/153; *B. bifidum*; *vs.* **Placebo:** n=114/130; dextrin.<br>**Dose and duration:** 2.5 × 10^9 viable cells of *B. bifidum* per day, in 2 divided doses.<br>**Type of milk:** EBM/ formula. **Type of delivery:** 91 (59.5%) *vs.* 103 (79.2%) p<0.05<br>**Outcomes of interest: Primary:** Time to reach enteral feeds >100ml/kg/day.

	**Secondary:** length of hospital stay, bodyweight at discharge, weight gain/day, HC at discharge, and increase in HC/hospital days.
**Van Niekerk et al**<br>2014<br>Single centre<br>South Africa	**Participants: HIV exposed** (GA: 24-28 weeks: 53% *vs.* 46%, 29-32 weeks: 44% *vs.* 51%, 33-36 weeks: 3% *vs.* 3%), **HIV non exposed** (24-28 weeks: 43% *vs.* 56%, 29-32 weeks: 53% *vs.* 40%, 33-36 weeks: 4% *vs.* 4%), **BW#: HIV exposed** (1009 ±153 g) *vs.* **HIV non exposed** (972±164 g); p=0.12<br>**Intervention and Comparison: Probiotic:** *L. rhamnosus GG* and *B. infantis* vs **Placebo**: Medium Chain Triglyceride (MCT) oil<br>N: 184; HIV exposed: 74 randomized: 37 *vs.* 37, HIV non- exposed: 110 randomized: 54 *vs.* 56<br>**Dose and duration:** Daily *L. rhamnosus* GG (0.35x 10^9 CFU) and *B. infantis* (0.35 x10^9 CFU) *vs.* MCT oil (5 drops), continued till 28 days postconceptional age<br>**Type of milk**: EBM *vs.* PDHM. **Type of delivery:** CS: HIV infected *vs.* uninfected: 58 (78%) *vs.* 81 (74%)<br>**Outcomes of interest: Primary:** feeding tolerance and growth<br>**Secondary:** NS
**Demirel et al**<br>2013<br>Single centre<br>Turkey	**Participants: GA#**: 29.4 ± 2.3 *vs.* 29.2 ± 2.5 wk. **BW#**: 1164 ± 261 *vs.* 1131 ± 284 g<br>**Intervention and Comparison: Probiotic:** n=135/138; S. *boulardii vs.* **Control:** n=136/140<br>**Dose and duration:** 5 billion CFU once daily till discharge<br>**Type of milk**: EBM/ formula. **Type of delivery:** CS: 105 (77.7%) *vs.* 113 (83.0%)<br>**Outcomes of interest: Primary:** NEC stage ≥2 and mortality<br>**Secondary:** clinical or culture-proven LOS, feeding difficulties and TFEF (days), weight gain
**Jacobs et al**<br>**PRO-PREMS** Study<br>2013<br>Multi centre<br>Australia	**Participants: GA#**: 27.9±2.0 *vs.* 27.8±2.0 wk, **BW#**: 1063±259 *vs.* 1048±260 g<br>**Intervention and Comparison: Probiotic:** n=548; *B. infantis*, (BB02300), *S. thermophilus* (TH4350) and *B. lactis* (BB12350) *vs.* **Placebo:** n=551; maltodextrin<br>**Dose and duration:** 1 x 10^9 total organisms twice daily until discharge from hospital or term corrected age<br>**Type of milk:** EBM/ formula. **Type of delivery: CS:** 359 (65.5%) *vs.* 377 (68.4%)<br>**Outcomes of interest: Primary:** incidence of at least 1 episode of definite LOS before 40 weeks postmenstrual age (PMA) or discharge home, whichever occurred first.<br>**Secondary:** Culture positive LOS, clinical LOS, Courses of antibiotics, Days of antibiotic treatment, NEC, Mortality, Length of primary hospital admission, TFEF, Days to regain birth weight, Weight at 28 d, Weight at discharge, PDA treated, IVH grade 3 or 4 or cystic PVL, ROP ≥grade 3, CLD at 28 days, BPD at 36 wk
**Serce et al**<br>2013<br>Single centre<br>Turkey	**Participants:** GA#: 28.8±2.2 *vs.* 28.7±2.1 weeks, BW#: 1126±232 *vs.* 1162±216 g<br>**Intervention and Comparison: Probiotic:** n=104 (*S. boulardii) vs.* **Placebo:** n=104 (distilled water)<br>**Dose and duration:** *S. boulardii (*10^9 organisms) twice daily *vs.* distilled water (1 ml twice daily). Commenced with first feed and continued till discharge. Median duration and follow up period: 44 days<br>**Type of milk**: EBM / formula. **Type of delivery:** CS: 84 (80.8%) *vs.* 92 (88.5%)<br>**Outcomes of interest: Primary:** NEC or LOS, NEC or mortality.<br>**Secondary:** TFEF (100 mL/kg/day), weight gain per week, oxygen dependency at 36 weeks, mortality until hospital discharge, and duration of hospitalization
**Al-Hosni et al**<br>2012<br>Multi centre<br>USA	**Participants: GA#**: 25.7±1.4 *vs.* 25.7±1.4 wk; p=0.97. **BW#**:778 ±138 *vs.* 779±126 g; p= 0.96<br>**Intervention and Comparison: Probiotic:** n=50 (*L. rhamnosus GG* and *B. infantis*) *vs.* **Control:** n=51 (no probiotic)<br>**Dose and duration:** 500 million CFU each of *L. rhamnosus* and *B. infantis* once daily, started from first feed and continued until discharge or until 34 weeks PMA

	**Type of milk**: NS. **Type of delivery:** CS: 22 (44%) *vs.* 30 (59%)<br>**Primary outcome**: improvement in growth (reduction in infants with BW<10th percentile at 34 weeks PMA)<br>**Secondary outcomes:** feeding tolerance, duration of antimicrobial treatment, probiotic safety and efficacy
**Chrzanowska-Liszewska et al**<br>2012<br>Single centre<br>Poland	**Participants:** GA(mean): 29.62 *vs.* 29.46 weeks; BW(mean): 1227.3 *vs.* 1282.5 g<br>**Intervention and Comparison: Probiotic:** n=21, *L. rhamnosus* GG ATCC 53103) *vs.* **Placebo:** n=26, maltodextrin<br>**Dose and duration:** 6×10^9 CFU once daily for 42 days<br>**Type of milk**: Formula. **Type of delivery:** CS: 16(77%) *vs.* 17(66%)<br>**Primary outcome**: difference in the amount of Bifidogenic microflora and *E. coli* in stool measured on day 7, 21, 42.<br>**Secondary outcomes:** effect on weight gain at discharge, hospital stay, use of antibiotics, NEC
**Sari et al**<br>2011<br>Single centre<br>Turkey	**Participants:** GA#: 29.5±2.4 *vs.* 29.7±2.4 wk, BW#: 1231±262 *vs.* 1278±282 g<br>**Intervention and Comparison: Probiotic:** n=110; *L. sporogenes vs.* **Control:** n=111<br>**Dose and duration:** 3.5 x 10^8 CFU once a day until discharged.<br>**Type of milk:** EBM/ formula**. Type of delivery:** CS: 74 (67.3%) 84 (75.7%)<br>**Outcomes of interest: Primary:** Death or stage ≥2 NEC<br>**Secondary:** culture-proven sepsis without NEC, IVH ≥grade 3, feeding intolerance, feeding amount per week, TFEF and weight gain per week.
**Indrio et al**<br>2008<br>Single centre<br>Italy	**Participants:** GA#: Formula+probiotic group: 34 wk. ± 1.1 *vs.* Formula+placebo group: 34 ±1.1 wk *vs.* BF group: 34 ±1.3wk. BW#: Formula+probiotic group:1890 ±432 g *vs.* Formula+placebo group:1850±342 g *vs.* BF group:1920±491g<br>**Intervention and Comparison:** Randomized 20 infants to **Probiotic:** n= 10 (formula with *L. reuteri ATCC 55730*) *vs.* **Placebo**: n=10 (formula with placebo). Also compared 10 breastfed infants(not randomized)<br>**Dose and duration:** 1x10^8 CFU per day commenced in between day3-5 of life, continued for 30 days<br>**Type of milk**: PTF in 20 randomized infants. **Type of delivery:** NS<br>**Primary outcome**: effect on feeding intolerance, bowel habit, and gastrointestinal motility patterns<br>**Secondary outcomes:** effect on weight gain
**Mohan et al**<br>2008<br>Single centre<br>India	**Participants: GA#**: 31.05 ±2.31 31.27±2.56 wk. **BW#**: 1449±343 *vs.* 1398±331 g<br>**Intervention and Comparison: Probiotic:** n=37 (B. *lactis* Bb12) vs **Placebo:** n=32 (human milk fortifier)<br>**Dose and duration:** *B. lactis* (2 x10^9CFU) *vs.* placebo. Probiotic group: day 1-3 (1.6 x 10^9 CFU daily) and day 4 onwards (4.8 x 10^9 CFU daily), Commenced within 24 hrs and continued till day 21<br>**Type of milk**: EBM/ PDHM. **Type of delivery: CS:** 86.5% *vs.* 90.6%<br>**Outcomes of interest: Primary:** effect on body weight, alteration of gut fermentation patterns, and immunologic parameters such as faecal calprotectin and IgA<br>**Secondary:** NS
**Stratiki et al**<br>2007<br>Single centre<br>Greece	**Participants: GA***: 31 (27-37) *vs.* 30.5 (26-37) wk; p=0.086, **BW***: 1500 (900-1780) *vs.*1500 (700-1900) g; p=0.915<br>**Intervention and Comparison: Probiotic:** n=41; *B. lactis* fortified PTF (2 × 10^7 CFU/g formula) vs **Placebo**: PTF only: n=34<br>**Dose and duration: Dose:** NS**;** supplement started within first two days of life, continued until discharge<br>**Type of milk**: PTF. **Type of delivery:** CS: 36.5% *vs.* 35%<br>**Primary outcome**: intestinal permeability<br>**Secondary outcomes:** Probiotic tolerance, somatic growth, LOS and NEC
**Bin-Nun et al**	**Participants: GA#**: 30±3 *vs.* 29±4 wk, **BW#**: 1152±262 *vs.* 1111±278 g

2005<br>Single centre<br>Israel	**Intervention, dose and Comparison: Probiotic:** n=72 (*B. infantis*, *Streptococcus (S.) thermophilus*, and *B. bifidus) vs.* **Control**: n=73 (no probiotics)<br>**Dose and duration:** daily 1.05 x 10^9 CFU (0.35 x 10^9 CFU B. *infantis*, 0.35 x 10^9 CFU *S. thermophilus*, and 0.35 x 10^9 CFU *B. bifidus* continued till 36 weeks postconceptional age<br>**Type of milk**: EBM/ formula. **Type of delivery:** CS: 56 (78%) *vs.* 57 (78%)<br>**Outcomes of interest: Primary:** NEC, **Secondary:** Weight gain, TFEF, LOS, duration of TPN
**Costalos et al**<br>2003<br>multicenter<br>Greece	**Participants: GA@**: 31.1 (2.5) *vs.* 31.8 (2.7) wk, **BW@**: 1651 (470) *vs.* 1644 (348.7) g<br>**Intervention and Comparison: Probiotic:** n=51; *S. boulardii* vs **Placebo**: n=36; maltodextrin.<br>**Dose and duration:** *S. boulardii:* 10^9 organisms twice daily; started with enteral feeds, median duration: 30 days<br>**Type of milk**: Formula. **Type of delivery:** CS: 49% *vs.* 38%<br>**Outcomes of interest: Primary:** Safety and tolerance of probiotic supplementation, effect on gastrointestinal function.<br>**Secondary:** NS
**Kitajima et al**<br>1997<br>Single centre<br>Japan	**Participants: GA#**: 28.3±2.3 *vs.* 28.2±2.1 wk, **BW#:** 1026±241 *vs.* 1026±205 g;<br>**Intervention and Comparison: Probiotic:** n= 45; *B. breve YIT4010 vs.* **Placebo:** n=46, distilled water.<br>**Dose and duration:** *B. breve YIT4010* (0.5 x 10^9CFU) given within the first 24 hours of life till 28 days<br>**Type of milk:** EBM / formula. **Type of delivery:** NS<br>**Outcomes: Primary:** Colonisation with *B. breve*<br>**Secondary:** Gas accumulation in stomach, vomiting, TFEF, feeding volume, weight gain, growth pattern, usage of indomethacin, use of antibiotics. (70 infants followed up till 3 years for growth)
**Reuman et al**<br>1986<br>Single centre<br>USA	**Participants: GA#**: 30.6 ±2.7 *vs.* 30.5±2.8 wk, **BW#**: 1366±302 *vs.* 1377±344 g<br>**Intervention and Comparison: Probiotic:** n=15; *L. acidophilus* fortified formula *vs.* **Placebo:** n=15; formula. (n=7 *vs.* 7 for growth outcomes). There was additional untreated group (infants whose mothers did not consent to study protocol matched by weight)<br>**Dose and duration:** 1ml of formula (6.8 x 10^8 to 11 x 10^8 organisms/ml) twice daily containing lactobacilli or placebo; duration: NS<br>**Type of milk:** EBM/ formula. **Type of delivery:** NS<br>**Outcomes of interest: Primary:** effect of lactobacilli on gut colonization by aminoglycoside resistant gram negative enteric organisms<br>**Secondary:** antibiotic utilization, daily formula volume, daily weight gain, hospital acquired infections

*(For all data: results presented as probiotics vs control/ placebo groups); @: median, interquartile range; #: mean (SD); $: median, interquartile range, range; *: median, range

Abbreviations: 3Di: developmental, dimensional and diagnostic interview; BF: breastfed; BL: body length; BPD: bronchopulmonary dysplasia; BSID-II: bayley's scale of infant development; BW: birth weight; CFU: colony forming units; CGA: corrected gestational age; CLD: chronic lung disease; CP: cerebral palsy; CS: caesarian section; db: decibels; DQ: developmental quotient; EBM: expressed breast milk; g: grams; GA: gestational age; HC: head circumference; HINE: hammersmith infant neurological examination; HIV: human immunodeficiency virus; IgA: immunoglobin A; IVH: intraventricular haemorrhage; LOS: late onset sepsis; MCT: medium chain triglycerides; MDI: mental development index; MSEL: mullen's scale of early learning; NDA: neurodevelopmental assessment; NEC: necrotizing enterocolitis; NEPSY-II: Developmental Neuropsychological assessment; NICU: neonatal intensive care unit; NS: not specified; PDHM: pasteurized donor human milk; PDI: psychomotor development index; PMA: postmenstrual age; PTF: preterm formula; PVL: periventricular leukomalacia; RCT: randomized controlled trial; ROP: retinopathy of prematurity; SCFA: short chain fatty acid; SD: standard deviation; SRS: social responsiveness scale; TFEF: time to full enteral feeds; TPN: total parenteral nutrition; VABS-II: vineland adaptive behavioral scale; VLBW: very low birth weight; wk: weeks; Wt: body weight

Supplemental Table 2: Reported outcomes of included studies on effects of probiotics on growth and neurodevelopment in preterm infants

**Studies reporting on *neurodevelopment only***	
**Study ID**	**Study characteristics**
**Agrawal et al**<br>2020<br>Single centre<br>Australia<br>Follow up of original study by Patole et al	**Outcomes: Primary: MSEL composite score** showed no difference between groups univariately or after adjustment for GA, IUGR, Apgar <7 at 5 min and age at assessment: (adjusted mean effect in probiotic group: -2.7, 95% CI: -8.5 to -3.0, p=0.349). Probiotic group had lower T scores in expressive language domain (adjusted mean effect: -4.5, 95% CI: -9.6 to -0.4, p=0.032), **Secondary:** No significant differences in the **3Di scores** between groups, **Tertiary:** No significant difference in outcome measures on **NEPSY-II**, **SRS** and **VABS-II** between groups.<br>**Author's conclusions:** No significant effect of probiotic on neurodevelopment of children assessed at age of 3 to 5 years. Validity of results limited by high rate of loss to follow up resulting in a small sample size.
**Akar et al**<br>2016<br>Single centre<br>Turkey	**Primary neurodevelopmental outcome:** N=124 *vs.* 125 (mean age of 21.7±2.4 months CGA), **Moderate to severe CP**: 8% *vs.* 8.8%; p=0.83, **MDI$:** 81(49-124) *vs.* 82(53-128); p=0.48, **PDI$:** 80(49-112) *vs.* 79(49-107); p=0.67, **NDI:** 29% *vs.* 29%; p=0.96, **MDI <70:** 20.9 % *vs.* 18.4%; p=0.61, **PDI<70:** 19.3% *vs.* 20.8%; p=0.77, **Bilateral Blindness:** 0% *vs.* 0%, **Bilateral Deafness:** 0.8% *vs.* 0%; p=0.31<br>**Other outcomes: N=**124 vs. 125**, BPD:** 23.3% *vs.* 38.7%; p=0.19**, LOS:** 6.4% *vs.* 15.2%; p=0.02**, NEC stage 2:** 0.8% *vs.* 4.8%: p=0.05, **IVH grade 3-4:** 1.6% *vs.* 5.6%: p=0.09**, severe ROP:** 5.6% *vs.* 12.8%; p=0.05<br>**Author's conclusions:** Oral probiotics did not affect neuromotor, neurosensory and cognitive outcomes at 18–24 months' CGA.
**Romeo et al**<br>2011<br>Single centre<br>Italy	**Primary outcome:** Enteric fungal colonization was significantly lower in the probiotic group vs. controls; p<0.05<br>**Other outcomes: Neurological outcome at 12 months CGA:** using the HINE: 202/ 249 had normal optimality scores (>73), 47 had suboptimal scores (<73) 10/ 83 vs. 13/83 vs. 24/83; p< 0.05 for probiotic vs. controls**, duration of hospital stay#:** 17.8±7.9 *vs.* 26.9±15.7 *vs.* 31.3±16.3 days; p<0.05 for *L. reuteri* vs. *L. rhamnosus* and *L. reuteri* vs. controls, fungal sepsis<br>**Author's conclusions:** Probiotics were effective in reducing abnormal neurological outcomes in preterms
**Studies reporting on *both neurodevelopment and long-term growth***	
**Totsu et al**<br>2018<br>Multi-centre<br>Japan<br>Follow up of original study by Totsu et al	**Primary outcome: TFEF@:** 11.0(9, 17.0) *vs.* 12.0(9.5, 16.0) days; p=0.654, **Wt at discharge#**: 2381.8±581.0 *vs.* 2876.8±499.2 g; p=NS, **HC at discharge@**: 34.5(33.8-35.5) vs. 34.8(33.7-36.0) cm; p=NS<br>**Neurodevelopmental outcomes: CP(in those who followed up):** 4/100 (4%) *vs.* 10/100 (10%); OR 0.375(95% CI: 0.114:1.238); p=0.108; **Developmental DQ18 score#:** 90.6±12.5 (n=54) *vs.* 91.1±14.4 (n=65), partial correlation coefficient (PCC): -0.443(95% CI: -5.384 to 4.499); p=0.859; **DQ 18< 85:** 24/89 (27%) vs. 32/79 (41%), OR 0.542(95% CI: 0.283-1.038); p=-0.065, **Subgroup analysis:** more favourable development was noted in probiotic vs placebo group, among the infants with a birth weight ≥1000 g, gestational age ≥28 weeks, caesarean delivery, antenatal steroid use, female sex or ≥13 days until full enteral feeding<br>**Other outcomes: NEC:** 0 vs 0; **LOS:** 6/102 (6%) *vs.* 12/105 (11%); p=0.218, **physical development at 18 months of age: Wt#**: 9.3±1.7 (n=98) vs. 9.2±1.2 kg (n=103); PCC: 0.177 (-0.277 to 0.581); p=0.39, **HC#:** 46.3±2.2 (n=80) vs. 46.5±1.8 cm (n=93): PCC: -0.259(95% CI: -0.864 to 0.347); p=0.401, **BL#:** 77.1±4.3 (n=97) vs. 77.2±4.2 cm (n=103), PCC: -0.148 (95%ci: -1.333 TO 1.038); p=0.806<br>**Author's conclusions:** *B. bifidum* OLB6378 may have a beneficial effect on the psychological development in VLBW infants
**Jacobs et al**<br>2017<br>**PRO-PREMS** Study<br>Multi-centre<br>Australia, New Zealand<br>Follow up of original study by Jacobs et al	**Primary neurodevelopmental outcome**: **Survival without neurosensory impairment at 2 years corrected age:** 281/373 (75.3%) *vs.* 271/362 (74.9%); relative risk (RR) 1.01 (95% CI 0.93 to 1.09); p=0.88, **Major neurosensory impairment:**56/337 (16.6%) *vs.* 56/327 (17.1%), RR: 0.97 (95% CI: 0.69-1.36); p=0.86, **Moderate/severe cerebral palsy** (Gross Motor Function Classification System score 2–5): **moderate CP:** 8% *vs.* 9.2%, **severe CP:** 0.3% *vs.* 1.5%, **motor impairment** (Bayley-III Motor Composite Scale <–2SD or Movement Assessment Battery for Children <15th centile if >42 months' CA): 9.3% *vs.* 7.4%, RR 1.25 (95% CI: 0.75-2.07); p=0.4, **cognitive impairment** (Bayley-III Composite Cognitive or Language Scales <–2SD or WPPSI FSIQ <–2SD if >42 months' CA): **Cognitive impairment**: 11.6% *vs.* 12.4%, RR 0.93 (95% CI: 0.62 to 1.41); p=0.74, **WPPSI scores: FSIQ#:** 106.0±21.6, n=37 (probiotic group); MD: 1.3 (-8.3 to 14.1; p=0.79), **FSIQ< 70:** 5.4% (n=37) *vs.* 4% (n=25); MD: 1.35 (0.1 to 14.1; p=0.8), **BSID-III cognitive scores#:** 100.4±17.1, n=299 *vs.* 99.2±15.1, n=298; Mean difference (MD): 1.2 (95% CI -1.4 to 3.8; p=0.36), **BSID-III**

	**motor scores#:** 102.3±11.6, n=299 *vs.* 100.7±16.8, n=296; MD: 1.6 (-1.1 to 4.3; p=0.24), **BSID-III language scores#**: 98.3±16.8, n=289 *vs.* 98.5±18.1, n=281; MD: -0.3 (-3.1 to 2.6; p=0.86), **blindness:** 0.3% *vs.* 0% or **deafness:** 0.6% *vs.* 3.4% RR: 0.18 (95%CI: 0.04-0.8; p=0.01)<br>**Secondary outcomes: Growth at mean age of 30 months#: Z scores** (n= 329 vs. 321): **Weight:** -0.6±1.3 *vs.* -0.6±1.3, **Height**: -0.2±1.3 *vs.* -0.2±1.2, **HC**: -1.2±1.3 *vs.* -1.1±1.4<br>**Other outcomes: NEC ≥ stage 2:** 2.4% vs. 5.5%, **IVH grade 3-4/ cystic PVL:** 4.6% *vs.* 2.8%, **LOS:** 15.3% *vs.* 15.2%, **BPD:** 33.7% *vs.* 32.7%, **ROP> stage 3:** 4.8% *vs.* 4.7%<br>**Author's conclusions:** Probiotic supplementation in very preterm infants did not adversely affect neurodevelopment or behavior in early childhood.
**Sari et al**<br>2012<br>Single centre<br>Turkey<br>Follow up of original study by Sari et al	**Primary outcome: Growth#**: **Wt**: 10.5±1.7 *vs.* 10.5±1.7 kg; p=0.92, **BL**: 79.4±7.8 *vs.* 81.0±5.3 cm; p=0.326, **HC**: 47.5±6.5 *vs.* 46.7±1.8 cm; p=0.53<br>**Neurodevelopmental outcomes:** CP (4.7% vs. 2.3%; P=0.441), Visual impairment (1.2% vs. 2.3%; p=1), Hearing impairment (1.2% vs. 1.1%; p=1), Mental development index (MDI): 90.7 ±15.5 vs. 90.4 ±14.5; p=0.887), MDI < 70 (14% vs. 11.4%; p=0.607), Psychomotor development index (PDI)#: 95.4±17.2 *vs.* 93.2±16.4; p=0.394, PDI<70 (10.5% vs. 10.2%; p=0.959), Overall NDI (18.6% vs. 17%; p=0.788)<br>**Other outcomes: NEC ≥ stage 2** (3.5% *vs.* 8%; p=0.33), **IVH grade 3-4** (9.3% *vs.* 10.2%; p=0.837), **cystic PVL** (3.5% *vs.* 2.3%; p=0.68**)**, **ROP** (5.8% *vs.* 4.5%; p=0.745), **CLD** (9.3% *vs.* 8%; p=0.793**), LOS** (27.9% *vs.* 21.6%; p=0.334), **duration of hospital stay** (38.3±22.6 *vs.* 36.1±25.4 days; p=0.541)<br>**Author's conclusions:** Oral probiotic did not affect long-term outcomes including neurodevelopment and growth at 18 to 22 months CGA
**Chou et al**<br>2010<br>Single centre<br>Taiwan	**Primary outcome:** n= 153 *vs.* 148: **Death/ NDI:** 29.4% *vs.* 33.1%; p=0.1, **Death:** 5.2% *vs.* 16.2 %; p=0.0002, **CP:** 5.2% *vs.* 2%; p=0.5; **Visual impairment:** 0.6% *vs.* 2.7%; p=0.2; **Deafness:** 1.3% *vs.* 0.6%; p=1; **BSID-II MDI** (mean ± 2SD): 87.9±18.1 *vs.* 88.±18.4; p=0.8, **MDI< 70:** 14.3% *vs.* 18.2%; p=0.3, **BSID-II PDI#**: 86.4±18.6 *vs.* 87.9±17.1; p=0.3, **PDI < 70:** 12.4% *vs.* 12.25 ; p=0.1<br>**Other outcomes: Growth at 3 years age#: Wt:** 11.2±1.9 *vs.* 11.9±1.7 kg; p=0.9, **height:** 84.4±5.2 *vs.* 84.4 ± 5.2 cm; p=1, **HC:** 46.2±1.7 *vs.* 46.3±3.7; p=1, **NEC stage 2:** 0% *vs.* 1.2%; p=0.2, **IVH grade 3-4:** 5.2% *vs.* 7.4%; P=0.4, **PVL:** 5.8% *vs.* 4.1%; p=0.6, **BPD:** 22.8% *vs.* 15.5%; p=0.2, **severe ROP:** 5.8% *vs.* 10.1%; p=0.1, **LOS:** 13.7% *vs.* 20.2%; p=0.1<br>**Author's conclusions:** Oral probiotics did not affect growth and neurodevelopmental and sensory outcomes at 3 years CGA.
**Studies reporting on *growth only***	
**Spreckels et al**<br>**PROPEL** trial<br>2021<br>Multi centre<br>Sweden	**Primary outcome:** Faecal colonisation rates in infants: 86% *vs.* 0% (week 1), 98% *vs.* 10% (in week 4), 88% *vs.* 4% at 36 weeks PMA. At 2-year follow-up it was 0% *vs.* 4%.<br>**Other Outcomes**: **TFEF$**: 14(10-18) *vs.* 15(11-19) days (graphical data), **NEC stage ≥2**: 4% *vs.* 9%, **LOS**: 35% *vs.* 30%, **BPD**: 60% *vs.* 60%, **ROP**: 22% *vs.* 11%, **Mortality**: 6% *vs.* 4%, **Growth**: **Head growth**: median (95% CI): -1.11(-0.86 to -1.35) *vs.* -1.78(-1.5 to -2.06); females had improved length growth until 4 weeks (p=0.007) and improved head growth until 2 (p=0.045) and 4 weeks of age (p=0.013).<br>**Safety**: No adverse effects<br>**Author's conclusion**: At least 86% of ELBW infants in the *L. reuteri* group were colonized independent of feeding. Higher concentrations of specific HMOs weekly co-related with lower abundance of *L. reuteri*. Within *L. reuteri* group higher abundance weekly corelated with shorter TFEF. Female sex and *L. reuteri* colonization improved head growth from birth to 4 weeks of age.
**Cui et al**<br>2019<br>Single centre<br>China	**Primary outcome: Growth#: Wt** gain: 14.55±3.07 *vs.* 10.12±2.80 g/day; p=0.000, **HC** gain: 0.0760±0.0157 *vs.* 0.0681±0.0108 cm/day; p=0.007, **BL** gain: 0.1878±0.0151 *vs.* 0.1756±0.0166 cm/day; p=0.000<br>**Feeding tolerance#:** Reflux: 2.18±0.83 *vs.* 3.77±0.66 times/day; p=0.000, TFEF: 9.95±2.46 *vs.* 13.80±3.47 days; p=0.015<br>**Other outcomes: Infection: LOS**: 4.44% *vs.* 8.33%; p=0.446, Localized infection: 6.67% *vs.* 8.33%; p=0.761, **NEC**: 2.22% *vs.* 10.42%; p=0.108, **Hospital stay (d):** 20.60±5.36 *vs.* 23.75±8.57; p=0.036, **Defecation (times/d):** 3.08±0.33 *vs.* 2.29±0.20; p=0.006<br>**Safety:** No adverse events

	**Author's conclusions:** *L. reuteri* improved early feeding tolerance, promoted growth and increased defaecation frequency whilst shortening hospital stay in preterm infants.
**Oshiro et al**<br>2019<br>Single centre<br>Japan	**Primary outcome: Wt gain:** The probiotic group showed significantly larger cumulative body weight gain by 8 weeks (p < 0.05) (graphical data only). **Faecal Bile Acid Concentration**: values were significantly lower in probiotic group at 4 and 8 weeks of life, (p<0.05). Probiotic group had significantly higher total **faecal bacterial counts**, including bifidobacteria; higher levels of total **faecal SCFAs** and nominally (but not significantly) higher concentrations of **plasma n–3 fatty acids**.<br>**Other outcomes: Sepsis:** n (%): 0(0%) vs. 3(16.7%), p>0.05, **NEC:** none<br>**Author's conclusions:** Bifidobacterial supplementation, concomitant with early feeding with maternal colostrum and breast milk, yielded the establishment of a beneficial microbiota profile. The associated changes in faecal organic acid levels, faecal pH, and bile acid levels appeared to provide improved growth in preterm infants.
**Wejryd et al**<br>**PROPEL** trial<br>2019<br>Multi Centre<br>Sweden	**Primary outcome: feed tolerance: TFEF (>150 mL/kg/day)** $^{@}$**:** 15(11–23) *vs.* 15(10–20) days; p=0.74, **TPN duration** (mean and 95%CI): 24.1(20.3-27.9) vs. 23.1(19.9-26.3) days; p=0.69, **gastric residuals week 1-4** (mean and 95% CI): 3(2.4-3.6); vs. 3.8(3.1-4.5); p=0.06<br>**Other outcomes: Growth at 28 days**: **Wt** gain: 340.5±216 vs. 323.8±167g; **BL** gain: 3.26±1.5 vs. 3.23±1.5cm; **HC** gain: 2.22±1.0 vs. 1.75±1.2cm. **Growth at 36 weeks**: **Wt** gain: 1565±361 vs. 1603±369g; **BL** gain: 10.5±2.4 vs. 10.7±2.3cm; **HC** gain: 8.2±1.4 vs. 7.9±1.9cm<br>**Growth at 2 weeks: Wt:** 868±165 vs. 896±168g; **BL**: 34.1±2.2 vs. 34.5±2.2cm; **HC**: 23.6±1.2 vs. 23.6±1.3cm. **At 4 weeks**: **Wt**: 1075±244 vs. 1055±243g; **BL**: 35.8±1.8 vs. 35.9±2.3cm; **HC**: 25.1±1.5 vs. 24.9±1.4cm.<br>**HC:** Z-score decreased in both groups from birth to day 28 of life, lesser rate of decrease in the *L. reuteri* vs. placebo group: 1.2 SD (95%CI: 1.4: 1.0) *vs.* 1.7 SD (95%CI: 2.0:1.5); p=0.001. From birth-day 28: HC increased by 2.3 cm (95% CI: 2.0–2.5) vs. increase by 1.8 cm (95% CI: 1.5–2.1) in the L. *reuteri vs.* control group (p=0.01). Girls showed better increase in HC: [1.2 SD (95% CI 1.4: 1.0] *vs.* boys [1.7 SD (95%CI: 1.9: 1.5); p<0.001].<br>**Stage 2-3 NEC:** 7/68 10% *vs.* 8/66 12%; p=0.74, **NEC≥ stage 3:** 4/68 (6%) *vs.* 7/66 (11%); p=0.32, **Culture proven LOS:** 25/68 (37%) *vs.* 23/66 (35%); p=0.82, **BPD:** 40/63 (63%) *vs.* 39/61 (64%); p=0.96, **ROP, grade 3–5:** 10/62(16%) *vs.* 6/63(10%), p=0.27, **IVH, grade 3–4:** 6/67 (9%) *vs.* 6/66(9%), p=0.98, **PVL:** 1/67 (1%) *vs.* 4/66(6%); p=0.21, **Death:** 5/68 (7%) *vs.* 5/66(8%), p=1.0<br>**Safety:** No adverse events<br>**Author's conclusions:** *L. reuteri* supplementation had no effect on feeding tolerance but improved head growth rate (p=0.009) during the first month of life thereby reducing risk of neurological impairment later.
**Indrio et al**<br>2017<br>Multi centre<br>Italy	**Primary outcome: Clinical Parameters** $^{\#}$: **Time to regain birth weight**: 6.4±1.6 vs. 7.3±1.3 days; p<0.05; **Weight at end of the study period**: 1955.3±653.4 vs. 1737.6±512 g; p<0.05. **TFEF**: 4.2±1.1 vs. 7.5±3.2 days; p<0.01. **Duration of hospitalization**: 13.4±2.2 vs. 22.4±3.2 days; p<0.01. **Duration of antibiotic treatment**: 4.2±4.3 vs. 12.5±7.2 days; p<0.01. **Stool frequency**: 2.5±0.7 vs. 2.8±0.9 episodes/day on last week; p<0.05. **Fecal cytokines** $^{\#}$: **IL-1beta**: 57.4±73.3 vs. 17.1±16.7 pg/ml; p=0.04; **IL-8**: 56.7±72.4 vs. 197.3±222.1 pg/ml; p=0.04. **IL-10**: 6.3±3.2 vs. 4.2±1.7 pg/ml; p=0.02. **IL-17**: 6.5±1.9 vs. 8.8±3.5 pg/ml; p=0.02. **Calprotectin**: 246.6±78.4 vs. 323.9±111.7 µg/g; p=0.01. **TNF-alpha**: 8.0±3.1 vs. 12.7±7.7 pg/ml; p=0.01. **IL-6**: 3.2±2.8 vs. 2.9±1.7 pg/ml; p=not significant.<br>**Gastric emptying parameters at end of study** $^{\#}$: **Half-emptying time**: 73.8±7.5 vs. 80.4±6.1 minutes; p=0.0004. **Fasting antral area**: 0.6±0.2 vs. 0.8±0.3 cm^2; p=0.009<br>**Other outcomes:** cost of treatment saved by reduction of hospitalization amounted to 2043 euros per infant<br>**Author's conclusions:** L. reuteri supplementation has an effective role in preventing feeding intolerance and improving gut motor and immune function development in bottle-fed stable preterm newborns. Another benefit from the use of probiotics is the reducing cost for the Health Care service.
**Shashidhar et al**<br>2017<br>Single centre<br>India	**Primary outcome: TFEF** $^{\#}$**:** 11.2±8.3 *vs.* 12.7±8.9; p=0.4.

	**Other outcomes: Wt gain/week#:** 31.1±27 vs. 39.5±32.3g; p=0.2. **Duration of hospital stay#**: 27.6±18.5 *vs.* 31.2±22.9 days; p=0.4. Duration of TPN#**:** 9.5±8.3 vs. 10.5±9 days; p=0.5. **Number of episodes of feed intolerance**@: 1 (0,2) *vs.* 1(0,2); p=1.0. **Number of withheld feeds**@: 21 (1,40.5) *vs.* 12 (0,48); p=0.8. **NEC ≥stage2**: n (%): 2 (4.1%) *vs.* 6 (12.5%); p=0.3. **Mortality**, n (%): 1(1.9%) *vs.* 3 (5.7%); p=0.6.<br>**Author's conclusions:** Probiotic supplementation does not seem to result in significant improvement of feed tolerance in VLBW newborns.
**Sukanyaa S et al**<br>2017<br>Single centre<br>India	**Primary outcomes:** Average weight gain was significantly better in probiotic group, monitored over period of 1 month of age (details NS): (Mean Difference/MD: 0.230±0.11g; 95% CI: -0.796 to -0.251; p<0.000)<br>**Other outcomes:** Significant reduction in duration of hospital stay: (MD -5.576± 2.233 days; p<0.016).<br>**Safety:** No adverse effects<br>**Author's conclusions:** Probiotics had beneficial effect on the growth of preterm infants.
**Hays et al**<br>2016<br>Multi-centre<br>France	**Primary outcome: Postnatal growth**: no significant differences in mean body weight at end of supplementation#: 1875±14 *vs.*1906±23g, p= 0.25. **Average daily weight gain:** 15.9±4.1 *vs.* 16.6±3.1 g/kg/day; p=0.17. No statistically significant differences in anthropometric measures (weight for age, length for age and HC for age at 41 weeks corrected z-score; p=NS) or body composition analysis at 41 weeks between the intervention groups.<br>**Other outcomes: Culture proven LOS:** 17/145 (11.7%) *vs.* 19/52 (37%); p=0.912, **Diversity index** (measure of dysbiosis): mean diversity scores were very similar (3.4±1.3) *vs.* (3.4±1.8); p=0.75. No statistically significant effect of the diversity index on daily weight gain, **Fecal calprotectin@**: Similar concentrations among the different treatment groups (Control: 183[94; 268] µg/g, *B. lactis* group: 200[126; 264] µg/g, *B. longum* group: 226[91; 300] µg/g, *B. lactis* + *B. longum* group: 232 [99; 275] µg/g, all three probiotics groups combined: 221[104; 275] µg/g; p=NS).<br>**Safety:** No adverse effects<br>**Author's conclusions:** Bifidobacterium supplementation did not improve postnatal growth in preterm infants.
**Xu et al**<br>2016<br>Single centre<br>China	**Primary outcome: Wt gain #:** 16.14±1.96 *vs.* 10.73±1.77 g/day; p=0.02, **HC gain#:** 0.74±0.03 *vs.* 0.72±0.04 cm/week; p=0.67, **Linear growth#:** 0.89±0.04 *vs.* 0.87±0.04 cm/week; p=0.17<br>**Other outcomes: Maximum enteral feeding volumes #:** 128.44±6.67 *vs.* 112.29±7.24 mL/kg/day; p=0.03, **TFEF #:** 0.37±0.13 *vs.* 1.70±0.45 days; p<0.01, **sepsis** (n [%]): 4 (7.8%) *vs.* 6 (12.2%); p=0.06, **GI symptoms** (regurgitation, vomiting, gastric residuals) (n [%]): 7/51 (13.7%) *vs.* 10/49 (20.4%); p=0.05, Hospital stay (days)#: 23.3±1.6 *vs.* 28.0±1.8; p=0.035<br>**Safety:** No adverse effects<br>**Author's conclusions:** Prophylactic *S. boulardii* improved weight gain, feeding tolerance, and had no adverse effects in preterm infants
**Choudhury et al**<br>2015<br>Single centre<br>Bangladesh	**Primary outcomes: TFEF#**: 13.71±3.4 *vs.* 16.53±6.13 days; p=0.04, **Wt at discharge#**: 1458.83±209.70 *vs.* 1363.86±216.23g; p=0.07. **Duration of hospital stay#**: 19.3±5.6 *vs.* 23.5±8.3 days; p=0.015<br>**Other outcomes**: NS<br>**Safety:** No adverse effects<br>**Author's conclusions**: Probiotics improved feed tolerance and decreased hospital stay but did not affect weight gain in preterm LBW babies
**Dilli et al**<br>2015<br>Multi centre<br>Turkey	**Primary outcome: NEC:** n (%) **Probiotic** *vs.* Prebiotic *vs.* Synbiotic *vs.* **Placebo** group: 2 (2%) *vs.* 12 (12%) *vs.* 4 (4%) *vs.* 18 (18%); overall p<0.001,<br>**Other outcomes: Growth velocity#: Wt gain**: 230±74 *vs.* 241±98.2 *vs.* 229±96 *vs.* 227±100 g/kg/week; p= 0.09**, BL gain**: 1.3±0.7 *vs.* 1.4±0.6 *vs.* 1.5±0.7 *vs.* 1.2±0.6 cm/week; p= 0.04, **HC gain**: 1.1±0.5 *vs.* 1.1±0.5 *vs.* 1.2±0.5 *vs.* 1.3±0.7 cm/week; p=0 .06. **Wt at discharge#**: 1979±309 *vs.* 2028±373 *vs.* 2037±297 *vs.* 2081±400 g, p=0.07 **RDS**: n (%): 64 (64%) *vs.* 56 (56%) *vs.* 64 (64%) *vs.* 73 (73%); p=0.09, **PDA**: 24 (24%) *vs.* 21 (21%) *vs.* 23 (23%) *vs.* 41 (41%), p=0.005. **IVH ≥grade 3**: 13 (13%) *vs.* 5 (5%) *vs.* 9 (9%) *vs.* 18 (18%); p=0.02, **Overall antibiotic treatment (days)@**: 7 (7-27) *vs.* 7 (7-27) *vs.* 7 (7-27) *vs.* 27 (7-42), p=0.0001, **Feeding intolerance**: 1 (1%) *vs.* 3 (3%) *vs.* 4 (4%) *vs.* 9 (9%), p=0.02, **BPD**: 25 (25%) *vs.* 16 (16%) *vs.* 21 (21%) *vs.* 32 (32%), p= 0.05 **ROP**: 0 (0%) *vs.* 2 (2%) *vs.* 2 (2%) *vs.* 3 (3%), p=0.48 **TFEF (100 mL/kg per day)@**: 13 (10-

	17) *vs.* 12 (9-18) *vs.* 15 (10-22) *vs.* 18 (12-25) days; p=<0.001; **150 mL/kg per day**@: 18 (14-23) *vs.* 17 (12-24) *vs.* 20 (14-30) *vs.* 25 (15-37) days; p<0.001, **LOS (clinical):** 29 (29%) *vs.* 23 (23%) *vs.* 26 (26%) *vs.* 45 (45%); p=0.004, **LOS (culture proven):** 8 (8%) *vs.* 10 (10%) *vs.* 8 (8%) *vs.* 13 (13%); p=0.60, **NICU stay@:** 37 (27-50) *vs.* 38 (27-53) *vs.* 42 (33-60) *vs.* 50 (31-70) days; p=0.002, **Mortality:** 3 (3%) *vs.* 2 (2%) *vs.* 3 (3%) *vs.* 12 (12%); p= 0.003<br>**Safety:** No adverse events<br>**Author's conclusions:** *B. lactis* and synbiotic (*B. lactis* plus inulin) but not prebiotic alone (inulin) decrease NEC in VLBW infants
**Shadkam et al**<br>2015<br>Single centre<br>Iran	**Primary outcome: TFEF#:** (n=29 *vs.* 28): 12.83±4.268 *vs.* 16.75±6.592 days; p=0.01. **NEC** n(%): 2(6.7%) *vs.* 11(36.7%); p=0.005<br>**Other outcomes**: **Wt at discharge#:** (n=29 *vs.* 28): 1756.55±146.39 *vs.* 1747.32±159.51g; p=0.821. **Supplementary Feeding Time#:** (n=30 *vs.* 30): 3.2±0.997 *vs.* 3.13±1.224 days; p=0.81. **Sepsis** n (%): 4(13.3%) *vs.* 10(33.4%); p=0.01. **Mortality** n (%): 1(3.3%) *vs.* 2(6.7%); p=0.5.<br>Jaundice n (%): 29(96.6%) *vs.* 26(86.7%); p=0.35.<br>**Safety:** No adverse effects reported.<br>**Author's conclusions:** *L. reuteri* could reduce the time to reach full enteral feeding while diminishing the incidence of NEC in very low birth weight premature infants.
**Patole et al**<br>2014<br>Single centre<br>Australia	**Primary outcome: Stool colonisation with B. *breve* M-16V:** timepoint 1: 29 (39%) vs. 2 (3%); p=0.001, timepoint 2: 67 (91%) vs. 25 (38%); p=0.001<br>**Other outcomes: Discharge Wt**$: (n=77 *vs.* 76): 2590 (2184–2990; 1565–4290) *vs.* 2565 (2303–3080; 1605–5074) g; p=0.539. **NEC≥ Stage II**: 0 *vs.* 1; p=0.497, **LOS** (culture proven): 17 (22%) *vs.* 12 (16%); p=0.410, **TFEF** (150 ml/kg/d)@: 12 (9–21;5–71) *vs.* 12 (8–16; 3–81) days; p= 0.306, **Length of hospital stay (weeks)$**: 10 (6–14; 2–61) *vs.* 10 (7–14; 3–60); p= 0.812, , **Early onset sepsis:** Suspected: 77 (100%) *vs.* 74 (98%), p=0.245, Proven: 4 (5%) *vs.* 2 (3%), p=0.681, **Duration of antibiotics**$: 3 (3–5; 2–14) *vs.* 3 (3–5; 3–18) days; p= 0.685, **LOS**: *Suspected* episodes: *None*: 48 (62%) *vs.* 43 (57%); p=0.744, *One*: 15 (20%) *vs.* 16 (21%), *Two or more*: 14 (18%) *vs.* 17 (22%). *Proven* episodes: *None*: 60 (78%) *vs.* 64 (84%); p=0.465, *One*: 12 (16%) *vs.* 10 (13%), *Two or more*: 5 (7%) *vs.* 2 (3%), **Duration of antibiotics**$: 7 (5–10; 3–21) *vs.* 6 (3–11; 2–33) days, p=0.296<br>**Safety:** No adverse effects.<br>**Author's conclusions:** *B. breve* M-16V is a suitable probiotic strain for routine use in preterm neonates.
**Totsu et al**<br>2014<br>Multi centre<br>Japan	**Primary outcome:** Postnatal day at which enteral feeding exceeded 100 mL/kg/day: 11.0±3.6 *vs.* 12.1±3.8 days; p<0.05.<br>**Other outcomes** (n= 119 *vs.* 114): **Length of hospital days#**: 92.3±44.5 *vs.* 92.9±40.2 days; p=NS. **Wt at discharge#**: 2831.8±581.0 *vs.* 2876.8±499.2 g; p=NS. **Wt gain/hospital days#**: 20.1±3.7 *vs.* 20.8±4.0 g/day; p=NS. **HC at discharge**@: 34.5 (33.8–35.5) *vs.* 34.8 (33.7–36.0) cm; p=NS. **Increased HC/hospital days**@: 0.10 (0.09–0.11) *vs.* 0.10 (0.09–0.12) cm; p=NS.<br>**Author's conclusions:** *B. bifidum* in VLBW infants accelerated the establishment of enteral feeding after birth without increasing the incidence of adverse effects
**Van Niekerk et al**<br>2014<br>Single centre<br>South Africa	**Primary outcomes: TFEF#**: HIV-exposed infants vs. controls: 10.19±4.055 *vs.* 9.68±3.46 days; p=0.56. HIV-unexposed group *vs.* controls: 9.63±2.42 *vs.* 11.14±4.15 days; p=0.022. **Feeding volumes on day 7#**: were significantly lower in HIV exposed infants receiving probiotics vs. control: 62.04±35.42 *vs.* 79.47±28.09 mL/kg; p=0.036; No difference in feeding volumes on day 14(p=0.84), 21(p=0.23) and 28(p=0.76). Feeding intolerance and abdominal distension: no difference.<br>**Growth outcomes:** The **HIV-exposed** group showed significantly higher z scores for length and head circumference at day 28 than the unexposed group (P=0.003 and P=0.03, respectively).<br>**Average Daily weight gain**^: There was no difference for treatment groups or HIV exposure. **HIV-exposed**^: 13.39; ±6.20 (10.22- 17.65) *vs.* 14.57; ±6.16 (9.98-17.00) g/kg; P=0.93. **HIV-unexposed**^: 13.37; ±5.99 (8.27-17.39) *vs.* 14.06; ±6.79 (9.32-18.05) g/kg, P=0.61.

	**Growth in HIV-unexposed group: Weight:** At **D7:** 994.934±164.7681 vs. 937.481±154.028g; At **D14**: 1021.240±180.678 vs. 1004.63±180.678g; At **D21**: 1144.962±184.580 vs. 1153.635±204.550g; At **D28**: 1284.67±212.16 vs. 1318.958±252.662g. **Length:** At **D7**: 36.673±2.468 vs. 37.023±2.396cm; At **D14**: 37.667±2.196 vs. 37.660±2,124cm; At **D21**: 38.36±2.163 vs. 38.390±2.347cm; At **D28**: 39.308±2.237 vs. 39.596±2.351cm. **Head circumference**: At **D7**: 26.147±1.393 vs. 26.365±1.409cm; At **D14**: 26.842±1.429 vs. 27.023±1.339cm; At **D21**: 27.66±1.503 vs. 27.853±1.579cm; At **D28**: 28.620±1.429 vs. 28.789±1.642cm<br>**Other outcomes:** NS<br>**Safety:** No adverse effects<br>**Author's conclusions:** Probiotic supplementation did not affect growth outcomes or feeding tolerance in HIV-exposed and non-exposed VLBW infants.
**Demirel et al**<br>2013<br>Single centre<br>Turkey	**Primary outcome: NEC stage ≥2**[&]: 6 (4.4%; 0.97–7.91) vs. 7 (5.1%; 1.44–8.86), 95% CI of differences: -0.65 to 5.12; p=1.000. **Death**[&]: 5 (3.7%; 0.52–6.88) vs. 5 (3.6%; 0.52–6.81), 95% CI of differences: -5.20 to 5.25; p=1.000<br>**Other outcomes: Wt gain** did not differ between the probiotic and control groups. **Mean Weight** At 14 days: mean [95%CI]: 1202 [1154.5–1249.5] *vs.* 1186 [1137.1–1234.9] g. At 28 days: 1369 [1314.6–1423.7] *vs.* 1378 [1323.5–1433.9] g. At 42 days: 1571 [1503.4–1639.8] *vs.* 1555 [1493.0–1617.6] g. At 56 days: 1685 [1608.9–1761.7] *vs.* 1654 [1599.3–1709.7] g<br>**Sepsis (clinical)**[&]: 47 (34.8%; 26.77–42.85) *vs.* 65 (47.8%; 39.39–56.19), 95% CI of differences: -25.34 to -0.62; p=0.030, **Sepsis (culture proven)**[&]: 20 (14.9%; 8.90–20.90) *vs.* 21 (15.4%; 9.34–21.46), 95% CI of differences: -3.00 to 9.00; p=0.906, **Feeding intolerance**[&]: 30 (22.9%; 15.21–29.23) *vs.* 62 (48.1%; 37.22–53.96), 95% CI: -35.02 to 1.17; p<0.001, **RDS:** 81 (60%) *vs.* 68 (50%); **BPD:** 19 (14%) *vs.* 22 (16.1%); **IVH (Grade ≥3):** 8 (5.9%) *vs.* 6 (4.4%); **PDA:** 39 (28.8%) *vs.* 38 (27.9%); **ROP:** 12 (8.8%) *vs.* 14 (10.2%), **Mechanical ventilation (days)**[@]**:** 3 (1–38) *vs.* 4 (1–40); **NCPAP duration**[@]: 4 (1–30) *vs.* 3 (1–35) days; **Oxygen therapy duration**[@]: 3 (1–72) *vs.* 3 (1–54) days; **Duration of antibiotic treatment**[@]:10 (0–50) *vs.* 10 (0–40) days; **Duration of hospitalization**[@]: 47 (6–120) *vs.* 43 (4–134) days<br>**Safety:** No adverse effects<br>**Author's conclusions:** *S. boulardii* supplementation did not reduce death or NEC but improved feeding intolerance and reduced the risk of clinical sepsis in VLBW infants.
**Jacobs et al**<br>**PRO-PREMS** Study<br>2013<br>Multi centre<br>Australia	**Primary outcome:** Infants with at least 1 episode of definite **LOS**, n (%):72 (13.1%) *vs.* 89 (16.2%); p=0.16. Subgroup analysis in ≥28 wk infants: 18 (5.5%) *vs.* 34 (10.8%); p=0.01.<br>**Other outcomes: Weight at 28 days**[#]**:** 1495.0±401.2 *vs.* 1446.0±379.2 g; p=0.04. **Wt at discharge**[#]: 2870.5±748.8 *vs.* 2864.0±738.9 g; p=0.89. **Infants with at least 1 episode of definite LOS with pathogens**, n (%): 38 (6.9%) *vs.* 48 (8.7%); p=0.27. **Infants with at least 1 episode of definite LOS with CoNS**, n (%): 40 (7.3%) *vs.* 43 (7.8%); p=0.75. **Infants with clinical late-onset sepsis**, n (%): 75 (13.7%) *vs.* 83 (15.1%); p=0.52. **Courses of antibiotics**[@]:1 (0–1) *vs.* 1 (0–1); p=0.78. **Days of antibiotic treatment**[@]: 2 (0–7) *vs.* 2 (0–8); p=0.64. **NEC** (Bell stage 2 or more), n (%): 11 (2.0%) *vs.* 24 (4.4%); p=0.03. **Mortality**, n (%): 27 (4.9%) *vs.* 28 (5.1%); p=0.91. **Length of hospital admission**[@]: 71 (54–92) *vs.* 74 (58–93) days; p=0.09. **Duration on parenteral nutrition**[@]:12 (8–17) *vs.* 12 (8–18); p=0.29. **Time to regain birth weight**[#]: 11.1±4.5 *vs.* 11.7±4.8 days; p=0.06. **PDA** treated, n (%): 159 (29%) *vs.* 171 (31%); p=0.47. **IVH grade 3 or 4 or cystic PVL**, n (%): 22 (4.0%) *vs.* 16 (2.9%); p=0.31. **ROP** ≥grade 3, n (%): 28 (5.1%) *vs.* 30 (5.4%); p=0.80. **CLD** at 28 days: n (%): 281 (53.1%) *vs.* 284 (53.3%); p=0.96. BPD at 36 wk, n (%): 165 (31.6%) *vs.* 161 (30.7%); p=0.74<br>**Author's conclusions:** *B. infantis*, *S. thermophilus*, and *B. lactis* significantly reduced NEC of Bell stage 2 or more in very preterm infants, but not definite late-onset sepsis or mortality. Treatment with this combination of probiotics appears to be safe.
**Serce et al**<br>2013<br>Single centre<br>Turkey	**Primary outcomes: NEC ≥stage 2:** 7 (6.7%) *vs.* 7 (6.7%); p=1, **Stage ≥ 2 NEC or culture positive LOS:** 24 (23%) *vs.* 30 (28.8%); p=0.34, **Stage ≥ 2 NEC or death:**8 (7.7%) *vs.* 10 (9.6%); p=0.62, **death due to stage ≥ 2 NEC:** 3 (2.8%) *vs.* 3 (2.8%); p=1, **Culture positive LOS:** 19 (18.3%) *vs.* 25 (24.3%); p=0.29<br>**Other outcomes: Wt gain** (g/week)[#]: 113±61 *vs.* 129±65; p=0.31, **TFEF (100 mL/kg/day)**[#]**:** 11±7 vs. 12±7 days; p=0.37, **Oxygen dependency at**

	**36wks PMA:** 12 (11.5%) *vs.* 11 (10.6%); p=0.82; **Duration of hospitalization**[@]: 39 (28–60) *vs.* 43 (29–60) days; p=0.62, **Deaths:** 5 (4.8%) *vs.* 4 (3.8%); p=0.74<br>**Safety:** No adverse effects<br>**Author's conclusions:** Probiotics did not decrease the incidence of NEC or LOS.
**Al-Hosni et al**<br>2012<br>Multi centre<br>USA	**Primary outcome:** No difference in the percentage of infants with weight <10th percentile at 34 weeks PMA [27/47 (58%) *vs.* 28/47 (60%); p=0.83]. Average daily volume of feeding (ml/kg) was lower compared to controls in first four weeks. **Average daily Wt gain**[#]: 14.3±7.4 *vs.*11.8±4.8 g; p= 0.06. **Overall growth velocity for cases with 28 days of data**[#]: 14.9±6.5 *vs.* 12.6±4.5 g/day; p=0.05.<br>In infants (BW 501–750 g): **average daily weight gain**[#]**:** 13.9±4.7 *vs.* 10.4±4.0 g; p=0.02, **Growth velocity**[#]**:** 16.8±4.7 *vs.* 13.1±4.1 g/day; p=0.01.<br>**Other outcomes:** no difference in other outcomes<br>**Safety:** no adverse effects<br>**Author's conclusions:** Probiotic supplementation improved growth velocity but not the percentage of infants with growth delay at 34 weeks PMA in ELBW infants
**Chrzanowska-Liszewska et al**<br>2012<br>Single centre<br>Poland	**Primary outcome: Number of stool samples positive** for *Lactobacillus* in the were significantly higher in probiotic group on study day7(p=0.014) and day21(p=0.03), but not significant on day42(p=0.587). **Number of samples isolated from stools**: *Enterobacteriaceae*: Day7=15 vs. 5; (p=0.004); Day21= 21 vs. 5; (p=0.000); D42=17 vs. 2; (p=0.000). *Enterococcus sp*: Day21=19 *vs.* 4; (p=0.000). *Staphylococcus sp*: 8 vs. 0; (p=0.001); Day42= 5 vs. 0; (p=0.011). No difference in *E.coli*, *Kl. Pneumoniae*, *Kl. Oxytoca*, *E. cloacae* and *E.faecalis* (p=Not Significant)<br>**Other outcomes: Wt gain on discharge**: No difference (p=0.567, 95% CI (−168,305)). **Mean hospital stay**: no statistical difference (49.9 *vs.* 46 days, p=0.421 95% CI (−13.43;5.71). **NEC**: No case identified in either group. **Use of prophylactic antibiotics**[#]: (ampicillin and netromycin) 4.95±1.4 *vs.* 5.27±1.51 days (p=0.46). No difference in 2nd line antibiotics (vancomycin and netromycin) p=0.829.<br>**Safety:** no adverse effects reported<br>**Author's conclusions:** Probiotic supplementation does not decrease the amount of pathogenic organisms, nor increase weight gain during enteral feeding, or decrease length of hospital stay.
**Sari et al**<br>2011<br>Single centre<br>Turkey	**Primary outcome: Death or NEC**, n (%): 9 (8.2%) *vs.* 13 (11.7%); p=0.515. **NEC stage ≥2**, n (%): 6 (5.5%) *vs.* 10 (9%); p=0.447. **Death attributable to NEC**, n (%): 0 *vs.* 1 (0.9%); p=1.000. **Death not attributable to NEC**, n (%): 3 (2.7%) *vs.* 3 (2.7%); p=1.000.<br>**Other outcomes: Weight gain at 14 days**[#]: 3.7±7.1 *vs.* 3.7±6.0 g/kg/day; p=0.977. **Weight gain at 28 days**[#]: 10.0±5.1 *vs.* 10.5±5.2 g/kg/day; p=0.555. **Weight gain at 42 days**[#]: 12.6±4.3 *vs.* 12.3±5.0 g/kg/day; p=0.769. **TFEF**[#]: 17.3±8.7 *vs.* 18.3±9.8; p=0.438. **Feeding intolerance**, n (%): 49 (44.5) *vs.* 70 (63.1); p=0.006. **Duration of total antibiotic treatment** (median): 11.5 *vs.* 10 days; p=0.268. **IVH ≥grade3**, n (%): 11 (10%) *vs.* 10 (9%); p=0.983. **NICU stay**(median): 34.5 *vs.* 30 days; p=0.919.<br>**Author's conclusions:** *L. sporogenes* supplementation at the dose of 3.5 x 10^8 CFU/day is not effective in reducing the incidence of death or NEC in VLBW infants, however, it could improve the feeding tolerance.
**Indrio et al**<br>2008<br>Single centre<br>Italy	**Primary outcome:** Probiotic *vs.* placebo had significant decrease in regurgitation[#] (2.1±0.9 *vs.* 4.2±1.1 episodes/day, p<0.01), mean daily crying time[#] (32±6 *vs.* 88±16 minutes/day, p<0.01), increased stool frequency[#] (3.7±0.5 *vs.* 2.1±0.4 episodes/day, p<0.05), significantly increased gastric emptying rate (graphical data; p<0.001), and reduced fasting antral area (graphical data only; p<0.001)<br>**Other outcomes: Wt gain per day**[#]: (formula+probiotic) 28±7.0 *vs.* (formula+placebo) 25±8.1 *vs.* (breast-fed) 30 ± 9.1 g/day<br>**Safety:** no adverse effects<br>**Author's conclusions:** *L. reuteri* ATCC 55730 supplementation improved feeding tolerance and gut function in formula-fed preterm infants
**Mohan et al**<br>2008	**Primary outcomes: Wt gain in infants receiving antibiotics**[#]**:** (1574±65 *vs.* 1375±74; p=0.001 on day 21), No effect on weight gain in **all infants**[#] :(1882±53 *vs.* 1836±71; p=0.062; on day 21), **weight gain in infants not on antibiotics:** (1900±78 *vs.* 1941±79; p=NS), **Total faecal SCFA**

Single centre<br>India	**concentration:** higher in probiotic group (p< 0.001) and differences were most pronounced in weeks 2 (p<0.013) and 3 (p<0.001), faecal a**cetate:** Significantly higher in the probiotic group (p<0.001) with more pronounced differences in the second (p<0.001) and third weeks (p<0.001). Significant differences even in those infants on antibiotics (p<0.001), **Faecal propionate** (p<0.04) **and butyrate** (p<0.026): higher in the probiotic group, **Faecal lactate:** 38% higher concentration (p<0.011) in probiotic group. Differences more pronounced in infants without antibiotic therapy (p<0.009), **Faecal acidity:** Probiotic group had a significantly lower pH#:5.68±0.09 *vs.* 6.38±0.10; p<0.001, **Faecal calprotectin levels:** significantly lower in probiotic group (p< 0.041), Significant difference for infants without antibiotic treatment (p< 0.007), **Total faecal IgA levels:** 44% higher in the probiotic (n=19) *vs.* placebo (n=16); p<0.021<br>**Other outcomes:** NS<br>**Safety:** no adverse effects<br>**Author's conclusions:** Oral supplementation with *B. lactis* Bb12 increased levels of fecal acetate, lactate, and total IgA and decreased fecal calprotectin. Only antibiotic treated infants showed significantly higher body weight in response to receiving probiotics.
**Stratiki et al**<br>2007<br>Single centre<br>Greece	**Primary outcome:** At day 30 **intestinal permeability** (IP) was significantly lower in the *B. lactis* supplemented PTF group (p=0.003).<br>**Faecal bifidobacterial concentration**: on **Day 7***: 9.6 (6.6–10.2) *vs.* 8.1 (6.3–10.1) log 10 cfu/g wet faeces; p<0.035, **Day 30***: 9.7 (7.5–10.3) *vs.* 8.9 (7.2–10.2) log 10 cfu/g wet faeces; p<0.075.<br>**Other outcomes:** No significant difference in somatic growth between the two groups with the exception of head growth<br>**Weight gain***: 28.3 (12–38) *vs.* 30(10–40) g/day; p=0.144. **Length gain***: 1.4 (0–3) *vs.* 1.5(0–3.5) cm/week; p=0.271. **Head growth***: 1.1(0.45–1.9) *vs.* 0.9(0–2) cm/week; p= 0.001; **Culture proven LOS**: 0 *vs.* 3; p=NS, **NEC:** 0 *vs.* 3; p= NS<br>**Safety:** no adverse effects<br>**Author's conclusions:** Bifidobacter supplemented infant formula decreased IP and increased head growth in preterm infants
**Bin-Nun et al**<br>2005<br>Single centre<br>Israel	**Primary outcome: NEC Stage 2 or 3**: 1/72 (1%) *vs.* 10/73 (14%) infants; p=0.013.<br>**Overall NEC:** 3 (4%) *vs.* 12 (16.4%); p=0.03, Bell staging#: 1.33±0.46 *vs.* 2.33±0.46; p=0.005, **NEC-associated mortality:** 0/3 *vs.* 3/12; p=0.87, **NEC and/or death:** 6/73 *vs.* 17/72; p =0.025<br>**Other outcomes: Wt gain:** Trend toward improved total weight gain in probiotic group. **Cumulative weight gain** (by 6 weeks) #: 691±208 *vs.* 594±239 g; p=NS, **Age reached full feeds**#: 14.6±8.7 *vs.* 17.5±13.6 days; p=0.13, **Culture proven LOS:** 31 *vs.* 24; p=0.28, **Duration of TPN**#: 16.6±9.3 *vs.* 18.6±13.2 days; p=0.29<br>**Safety:** nil adverse effects<br>**Author's conclusions:** PS reduced both the incidence and severity of NEC in VLBW preterm infants
**Costalos et al**<br>2003<br>multicenter<br>Greece	**Primary outcome: Wt gain**@: 163.5(17.7) *vs.* 155.8 (16.5) g/week; p>0.05; **LOS:** 3/51 (5.8%) *vs.* 3/36 (8.3%), OR: 0.7 (95% CI 0.13– 3.6); p= NS; **NEC**: 5/51 (9.8%) *vs.* 6/36 (16), OR: 0.5 (95% CI 0.15– 1.98); p=NS<br>No significant difference in **age at which feeds were first offered** (3.2 (2) *vs.* 2.4 (2.1); p>0.1), in **TFEF@** (9.3 (2.7) *vs.* 9.9 (4.5); p>0.1), in **milk intake per day**@: 155 (15) *vs.* 148 (13) ml/kg/day; p>0.1, **stool steatocrit value@:** 64% (3.05) *vs.* 65% (2.72); p>0.5 and in **blood D-xylose levels@:** 1.5 (0.4) *vs.* 1.35 (0.3) mmol/l; p>0.1)<br>**Other outcomes:** NS<br>**Safety:** no adverse effects<br>**Author's conclusions:** Probiotic supplemented formula had a beneficial effect on stool flora but did not improve D-xylose or lipid absorption.
**Kitajima et al**<br>1997<br>Single centre	**Primary outcome: BBG Colonisation** (n=58/91) timepoints: 73% *vs.* 12% (at 2 weeks), 91% *vs.* 44% (at 6 weeks); **GA 23-25 wks.:** 80% *vs.* 0%; **26-28 wks.**: 87% *vs.* 50%, **29-33 wks.;** 100% *vs.* 45%, Colonisation at 2 weeks better in BBG group (24/33 *vs.* 3/25; p=NS), Colonisation rate was slower in <26 wks. infants and poorer in BBG group receiving antibiotics ≥10 days (n=3) at six weeks

Japan	**Other clinical outcomes:** i) **Preterm infants:** (GA 26 - 28 wks.): Mean aspirated air volumes were significantly less in BBG group in the first four wks. (ml/infant/week: 385 *vs.* 495, p<0.05). No difference in time interval between starting feeds and body weight gain.<br>Vomiting (times/group/week: 77 *vs.* 163; p=NS) and apnoea (times/group/week: 1334 *vs.* 1811, p=NS) lesser in BBG group, ii). **Fully colonised vs. non-colonised infants:** Indomethacin doses: 8 *vs.* 25; p=0.06. Reduced stomach gas accumulation, less vomiting, early feeding establishment (graphical data only) and greater feeding volume in fully colonised infants (graphical data only). **Wt gain** significantly greater in colonised infants between 4 and 8 weeks of life (week 4 and 8: p<0.05, week 5: p<0.02, week 6 and 7: p<0.001), Better growth pattern till 18 months in BBG group; p=NS<br>**Author's conclusions:** *B. breve* effectively colonized the immature bowel and was associated with fewer abnormal abdominal signs and better weight gain in VLBW infants
**Reuman et al**<br>1986<br>Single centre<br>USA	**Primary outcome:** Isolation of Lactobacilli from rectal swab cultures: 13/15 *vs.* 3/15, Isolation of gram-negative enteric organisms: during 40 of 86 weeks (47%) *vs.* 28 of 57 weeks (49%) of hospitalization<br>**Other outcomes: Average weight gain**#: n=7 *vs.* 7: 16±5 *vs.* 15±7 g/day, **duration of hospitalization**#: 59.4±56.4 *vs.* 38.7±30.6 days; p=NS, **Morbidity score**#: 7.3±4.1 *vs.* 6.9±6.6; p=NS, **Avg. formula volume**#: 115±92 *vs.* 133±83 ml/day; p=NS, **Days not fed orally**#: 13.9±11.9 *vs.* 13.5±22.1; p=NS, **Mortality**: 1 *vs.* 3; p=NS, **Days receiving ampicillin**#: 7.2±5 *vs.* 7.6±7.8; p=NS, **Days receiving gentamicin**#: 10±10.4 *vs.* 6±6.9; p=NS<br>**Author's conclusions:** Oral probiotics did not reduce facultative gram negative enteric bacterial colonization

*(For all data: results presented as probiotics vs control/ placebo groups); @: median interquartile range; #: mean±SD, $: median, interquartile range, range; ^: median, ±SD, interquartile range; &: n (percentage; 95% confidence interval); *: median, range

None of the included studies reported any adverse events

Abbreviations: 3Di: developmental, dimensional and diagnostic interview; BBG: Bifidobacterium breve YIT4010; BL: body length; BPD: bronchopulmonary dysplasia; BSID: bayley's scale of infant development; BW: birth weight; CFU: colony forming units; CGA: corrected gestational age; CI: confidence interval; CLD: chronic lung disease; CP: cerebral palsy; DQ: developmental quotient; ELBW: extreme low birth weight; FSIQ: full scale intelligent quotient; GA: gestational age; GI: gastrointestinal; HC: head circumference; HINE: hammersmith infant neurological examination; HIV: human immunodeficiency virus; HMOs: human milk oligosaccharides; IgA: immunoglobin A; IL: interleukin; IVH: intraventricular haemorrhage; IUGR: intrauterine growth restriction; LBW: low birth weight; LOS: late onset sepsis; MD: mean difference; MDI: mental development index; MSEL: mullen's scale of early learning; NCPAP: nasal continuous positive airway pressure; NDI: neurodevelopmental impairment; NEC: necrotizing enterocolitis; NEPSY-II: Developmental Neuropsychological assessment; NICU: neonatal intensive care unit; NS: not specified; PCC: partial correlation coefficient; PCR: polymerase chain reaction; PDA: patent ductus arteriosus; PDI: psychomotor development index; PMA: postmenstrual age; PVL: periventricular leukomalacia; RCT: randomized controlled trial; rDNA: ribosomal deoxyribonucleic acid; RDS: respiratory distress syndrome; ROP: retinopathy of prematurity; SCFA: short chain fatty acid; SD: standard deviation; SRS: social responsiveness scale; TFEF: time to full enteral feeds; TNF: tumour necrosis factor; TPN: total parenteral nutrition; VABS-II: vineland adaptive behavioral scale; VLBW: very low birth weight; WPPSI: Wechsler preschool and primary scale of intelligence; Wt: body weight

Supplemental Table 3: Overview of other clinical outcomes from included studies

Study ID	LOS	BPD	NEC ≥stage II	IVH or cPVL	ROP	TFEF	Duration of hospital stay	Death
Agrawal 2020	.	.	.	.	.	.	.	
Akar 2016	↓	↔	↓	↔	↓	.	.	
Al-Hosni 2012	.	.	.	.	.	.	.	.
Bin Nun 2005	↔	.	↓	.	.	↔	.	↓
Chrzanowska-Liszewska 2012	.	.	↔	.	.	.	↔	.
Chou 2010	.	.	.	.	.	.	.	.
Choudhary 2015	.	.	.	.	.	↓	↓	.
Costalos 2003	↔	.	↔	.	.	↔	.	.
Cui 2019	↔	.	↔	.	.	↓	↓	.
Demirel 2013	↔	↔	↔	↔	↔	↓	↔	↔
^Dilli 2015	↔/↓	↓	↓	↓	↔	↓	↓	↓
Hays 2016	↔	.	.	.	.	.	.	.
Indrio 2008	.	.	.	.	.	.	.	.
Indrio 2017	.	.	.	.	.	↓	↓	.
[$]Jacobs 2013	↔/↓	↔	↓	↔	↔	.	↔	↔
Jacobs 2017	.	.	.	.	.	.	.	
Kitajima 1997	.	.	.	.	.	.	.	.
[%]Mohan 2008	.	.	.	.	.	.	.	.
Oshiro 2019	↔	.	↔	.	.	.	.	.
Patole 2014	↔	.	↔	.	.	↔	↔	.
Reuman 1986	↔	↔	↔	↔	↔	.	↔	↔
Romeo 2011	*↔	.	.	.	.		↓	
Sari 2011	.	.	↔	.	.	↔	↔	↔
Sari 2012	↔	↔	↔	↔	↔	↔	↔	
Serce 2013	↔	↔	↔	.	.	↔	↔	↔
Shadkam 2015	↓	.	↓	.	.	↓	.	.
Shashidhar 2017	.	.	↔	.	.	↔	↔	↔
#Spreckels 2021	↔	↔	↔	.	↓	↔	.	↔
Stratiki 2007	↔	.	↔	.	.	.	.	.
Sukanyaa 2017	.	.	.	.	.	.	↓	.
Totsu 2014	.	.	.	.	.	↓	↔	.
Totsu 2018	↔	.	.	.	.	↔	.	
~Van Niekerk 2014	.	.	.	.	.	↔/↓	.	.
#Wejryd 2019	↔	↔	↔	↔	↔	↔	.	↔
Xu 2016	↔	.	.	.	.	↓	.	.

↑: significant increase in PS, ↓: significant decrease in PS; ↔: no significant difference between groups, ·: not reported

*: including fungal sepsis, # Spreckels and Wejryd are two different publications from the same RCT (PROPEL)

^Dilli et al reported significant reduction of clinical LOS in PS but culture proven LOS was comparable between groups

~: Van Niekerk et al reported reduced TFEF in HIV-unexposed PS vs. controls and comparable TFEF in HIV-exposed PS vs. control groups

[$]: Jacobs et al (2013) reported significant reduction in LOS in subgroup of >28 weeks PS, and comparable incidence of LOS in <28 weeks gestation; Reported significant weight gain in PS at 28 days and comparable weight gain between groups at discharge
[%]: Mohan et al reported significant increase in weight gain in PS receiving antibiotics vs controls, no difference in weight gain in all infants
Abbreviations: cPVL: cystic periventricular leukomalacia; BPD: bronchopulmonary dysplasia; NEC: necrotizing enterocolitis; ROP: retinopathy of prematurity; IVH: intraventricular haemorrhage; TFEF: time to full enteral feeds; PS: probiotic supplemented infants

Supplemental Table 4: Summary of results of meta-analysis

Outcome or Subgroup	Studies	Participants	Statistical Method	Effect Estimate	P value	Heterogeneity
**1. Short term Growth**						
1.1 Short term weight gain	22	3721	SMD (IV, REM, 95% CI)	0.24 [0.04, 0.44]	0.02	88%
1.2 Short term length gain	7	899	SMD (IV, REM, 95% CI)	0.12 [-0.13, 0.36]	0.35	69%
1.3 Short term head circumference gain	8	1132	SMD (IV, REM, 95% CI)	0.09 [-0.15, 0.34]	0.46	76%
**2. Long Term Growth**						
2.1 Long term weight gain	4	1326	SMD (IV, REM, 95% CI)	-0.08 [-0.29, 0.12]	0.42	68%
2.2 Long term length gain	4	1325	SMD (IV, REM, 95% CI)	-0.03 [-0.14, 0.07]	0.53	0%
2.3 Long term head circumference gain	4	1298	SMD (IV, REM, 95% CI)	-0.04 [-0.14, 0.07]	0.52	0%
**3. Overall neurodevelopment**						
3.5.1 Neurodevelopmental impairment	5	1556	RR (M-H, REM, 95% CI)	0.91 [0.76, 1.08]	0.27	0%
3.5.2 Cerebral palsy	5	1588	RR (M-H, REM, 95% CI)	1.11 [0.64, 1.91]	0.70	30%
3.5.3 Hearing impairment	4	1388	RR (M-H, REM, 95% CI)	0.7 [0.17, 2.95]	0.62	35%
3.5.4 Visual impairment	4	1388	RR (M-H, REM, 95% CI)	0.52 [0.12, 2.21]	0.38	0%
**4. Cognitive and motor impairment**						
4.1.1 Cognitive impairment	4	1388	RR (M-H, REM, 95% CI)	0.98 [0.75, 1.26]	0.85	0%
4.1.2 Motor impairment	4	1388	RR (M-H, REM, 95% CI)	1.06 [0.79, 1.41]	0.71	0%
4.2.1 Mean cognitive scores	5	1507	MD (IV, REM, 95% CI)	0.13 [-1.41, 1.67]	0.16	0%
4.2.2 Mean motor scores	4	1388	MD (IV, REM, 95% CI)	1.04 [-0.43, 2.50]	0.26	0%
**Subgroup analysis**						
1.1.1 Short term weight gain in single strain probiotics	15	1916	SMD (IV, REM, 95% CI)	0.34 [0.02, 0.65]	0.04	91%
1.1.2 Short term weight gain in multi strain probiotics	7	1805	SMD (IV, REM, 95% CI)	0.08 [-0.12, 0.27]	0.46	64%
5.1 Short term weight gain in <28 week infants	3	335	SMD (IV, REM, 95% CI)	0.05 [-0.29, 0.38]	0.79	60%
5.2 Short term length gain in <28 week infants	2	234	SMD (IV, REM, 95% CI)	-0.10 [-0.36, 0.15]	0.43	0%
5.3 Short term head circumference gain in <28 week infants	2	234	SMD (IV, REM, 95% CI)	0.04 [-0.24, 0.32]	0.77	17%

SMD: standard mean difference; RR: risk ratio; MD: mean difference; M-H: Mantel Haenszel; REM: random effects model; IV: inverse variance; CI: confidence interval

Supplemental Figure 1: Forest plot illustrating effect of probiotics on short-term length gain in preterm infants

Study or Subgroup	Std. Mean Difference	SE	Probiotics Total	Control Total	Weight	Std. Mean Difference IV, Random, 95% CI
Cui 2019	0.7613	0.2152	45	48	13.0%	0.76 [0.34, 1.18]
Dilli 2015	0.1528	0.1416	100	100	16.6%	0.15 [-0.12, 0.43]
Hays 2016	-0.1057	0.1617	145	52	15.6%	-0.11 [-0.42, 0.21]
Stratiki 2007	-0.2276	0.2327	41	34	12.2%	-0.23 [-0.68, 0.23]
Van Niekerk 2014	-0.1246	0.1909	54	56	14.2%	-0.12 [-0.50, 0.25]
Wejryd 2019	-0.0845	0.1797	63	61	14.7%	-0.08 [-0.44, 0.27]
Xu 2016	0.4962	0.2032	51	49	13.6%	0.50 [0.10, 0.89]
**Total (95% CI)**			**499**	**400**	**100.0%**	**0.12 [-0.13, 0.36]**

Heterogeneity: Tau² = 0.08; Chi² = 19.42, df = 6 (P = 0.004); I² = 69%
Test for overall effect: Z = 0.93 (P = 0.35)

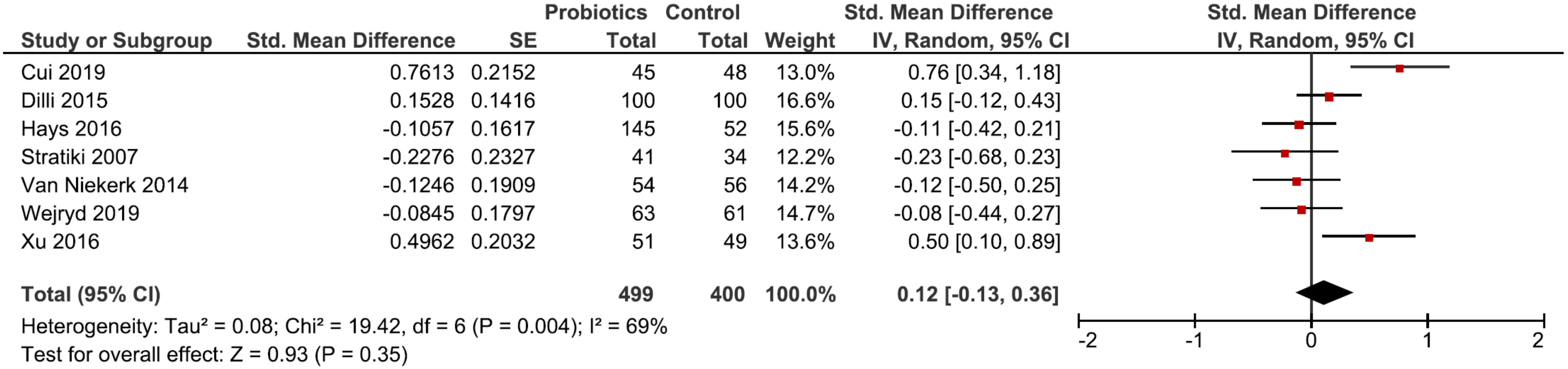

Supplemental Figure 2: Forest plot illustrating effect of probiotics on increase in short-term head circumference in preterm infants

Study or Subgroup	Std. Mean Difference	SE	Probiotics Total	Control Total	Weight	Std. Mean Difference IV, Random, 95% CI
Cui 2019	0.5849	0.2121	45	48	11.4%	0.58 [0.17, 1.00]
Dilli 2015	-0.3276	0.1424	100	100	13.9%	-0.33 [-0.61, -0.05]
Hays 2016	-0.2183	0.162	145	52	13.2%	-0.22 [-0.54, 0.10]
Stratiki 2007	0.4559	0.2351	41	34	10.7%	0.46 [-0.00, 0.92]
Totsu 2014	-0.1594	0.1313	119	114	14.3%	-0.16 [-0.42, 0.10]
Van Niekerk 2014	-0.1089	0.1909	54	56	12.2%	-0.11 [-0.48, 0.27]
Wejryd 2019	0.1791	0.18	63	61	12.6%	0.18 [-0.17, 0.53]
Xu 2016	0.563	0.2041	51	49	11.7%	0.56 [0.16, 0.96]
**Total (95% CI)**			**618**	**514**	**100.0%**	**0.09 [-0.15, 0.34]**

Heterogeneity: Tau² = 0.10; Chi² = 28.82, df = 7 (P = 0.0002); I² = 76%

Test for overall effect: Z = 0.74 (P = 0.46)

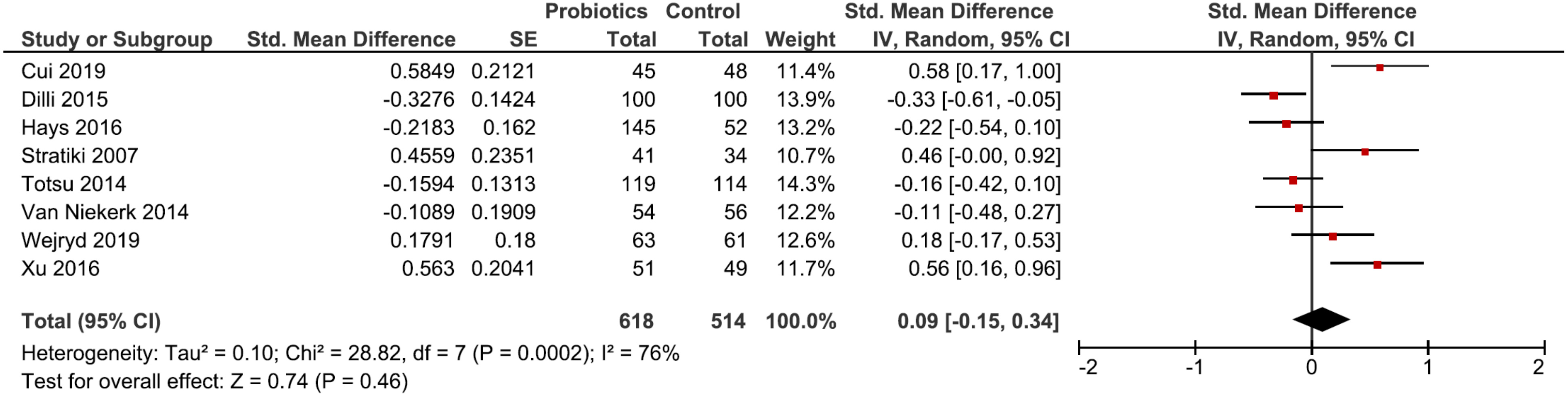

Supplemental Figure 3: Forest plot illustrating effect of probiotics on long-term weight gain in preterm infants

Study or Subgroup	Std. Mean Difference	SE	Probiotics Total	Control Total	Weight	Std. Mean Difference IV, Random, 95% CI
Chou 2010	-0.387	0.1164	153	148	25.7%	-0.39 [-0.62, -0.16]
Jacobs 2017	0	0.0785	329	321	31.1%	0.00 [-0.15, 0.15]
Sari 2012	0	0.1516	86	88	21.0%	0.00 [-0.30, 0.30]
Totsu 2018	0.068	0.1412	98	103	22.3%	0.07 [-0.21, 0.34]
**Total (95% CI)**			**666**	**660**	**100.0%**	**-0.08 [-0.29, 0.12]**

Heterogeneity: Tau² = 0.03; Chi² = 9.37, df = 3 (P = 0.02); I² = 68%

Test for overall effect: Z = 0.81 (P = 0.42)

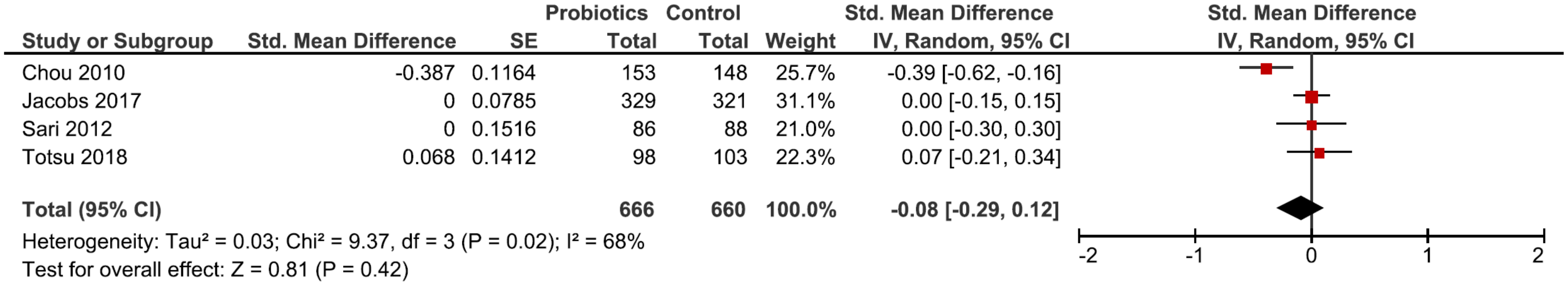

Supplemental Figure 4: Forest plot illustrating effect of probiotics on long-term linear growth in preterm infants.

Study or Subgroup	Std. Mean Difference	SE	Probiotics Total	Control Total	Weight	Std. Mean Difference IV, Random, 95% CI
Chou 2010	0	0.1153	153	148	22.8%	0.00 [-0.23, 0.23]
Jacobs 2017	0	0.0785	329	321	49.1%	0.00 [-0.15, 0.15]
Sari 2012	-0.2394	0.1522	86	88	13.1%	-0.24 [-0.54, 0.06]
Totsu 2018	-0.0234	0.1415	97	103	15.1%	-0.02 [-0.30, 0.25]
**Total (95% CI)**			**665**	**660**	**100.0%**	**-0.03 [-0.14, 0.07]**

Heterogeneity: Tau² = 0.00; Chi² = 2.10, df = 3 (P = 0.55); I² = 0%

Test for overall effect: Z = 0.63 (P = 0.53)

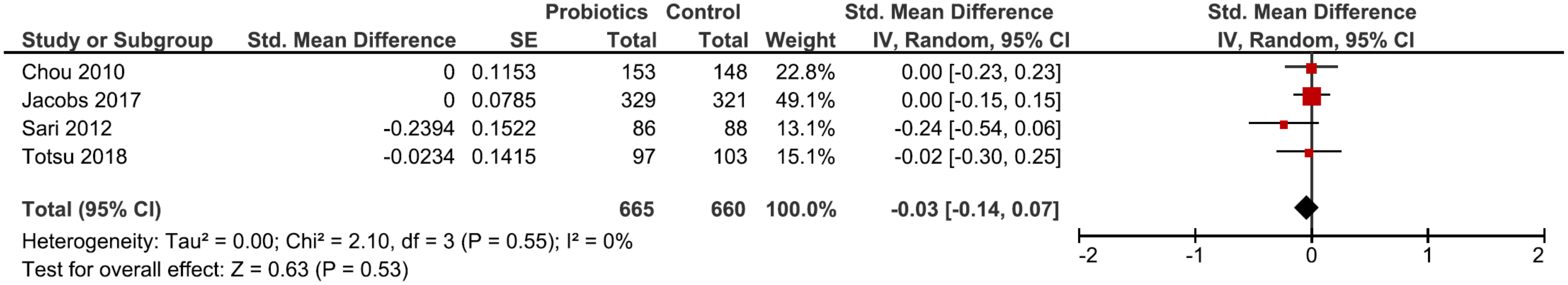

Supplemental Figure 5: Forest plot illustrating effect of probiotics on long-term head growth in preterm infants.

Study or Subgroup	Std. Mean Difference	SE	Probiotics Total	Control Total	Weight	Std. Mean Difference IV, Random, 95% CI
Chou 2010	-0.0348	0.1153	153	148	23.2%	-0.03 [-0.26, 0.19]
Jacobs 2017	-0.074	0.0785	329	321	50.1%	-0.07 [-0.23, 0.08]
Sari 2012	0.1679	0.1519	86	88	13.4%	0.17 [-0.13, 0.47]
Totsu 2018	-0.0998	0.1526	80	93	13.3%	-0.10 [-0.40, 0.20]
**Total (95% CI)**			**648**	**650**	**100.0%**	**-0.04 [-0.14, 0.07]**

Heterogeneity: Tau² = 0.00; Chi² = 2.21, df = 3 (P = 0.53); I² = 0%

Test for overall effect: Z = 0.65 (P = 0.52)

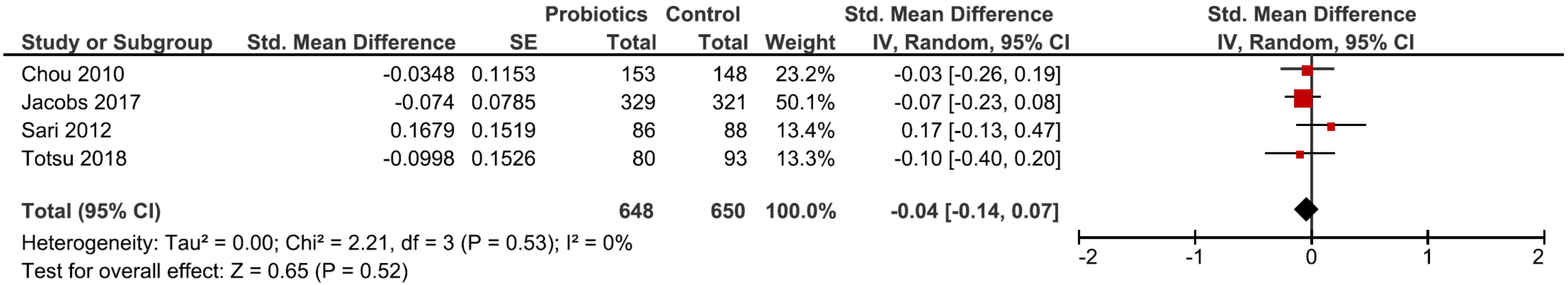

Supplemental Figure 6: Forest plot illustrating effect of probiotics on cognitive and motor impairment outcomes in preterm infants.

	Probiotics		Control			Risk Ratio
**Study or Subgroup**	**Events**	**Total**	**Events**	**Total**	**Weight**	**M-H, Random, 95% CI**
**4.1.1 Cognitive impairment**						
Akar 2017	26	124	23	125	14.6%	1.14 [0.69, 1.88]
Chou 2010	22	153	27	148	13.9%	0.79 [0.47, 1.32]
Jacobs 2017	39	337	40	327	21.5%	0.95 [0.63, 1.43]
Sari 2012	12	86	10	88	6.0%	1.23 [0.56, 2.69]
**Subtotal (95% CI)**		**700**		**688**	**55.9%**	**0.98 [0.75, 1.26]**
Total events	99		100			
Heterogeneity: Tau² = 0.00; Chi² = 1.38, df = 3 (P = 0.71); I² = 0%						
Test for overall effect: Z = 0.19 (P = 0.85)						
**4.1.2 Motor impairment**						
Akar 2017	24	124	26	125	15.0%	0.93 [0.57, 1.53]
Chou 2010	19	153	18	148	10.1%	1.02 [0.56, 1.87]
Jacobs 2017	31	337	24	327	14.1%	1.25 [0.75, 2.09]
Sari 2012	9	86	9	88	4.8%	1.02 [0.43, 2.45]
**Subtotal (95% CI)**		**700**		**688**	**44.1%**	**1.06 [0.79, 1.41]**
Total events	83		77			
Heterogeneity: Tau² = 0.00; Chi² = 0.70, df = 3 (P = 0.87); I² = 0%						
Test for overall effect: Z = 0.37 (P = 0.71)						
**Total (95% CI)**		**1400**		**1376**	**100.0%**	**1.01 [0.83, 1.22]**
Total events	182		177			
Heterogeneity: Tau² = 0.00; Chi² = 2.24, df = 7 (P = 0.95); I² = 0%						
Test for overall effect: Z = 0.11 (P = 0.91)						
Test for subgroup differences: Chi² = 0.16, df = 1 (P = 0.69), I² = 0%						

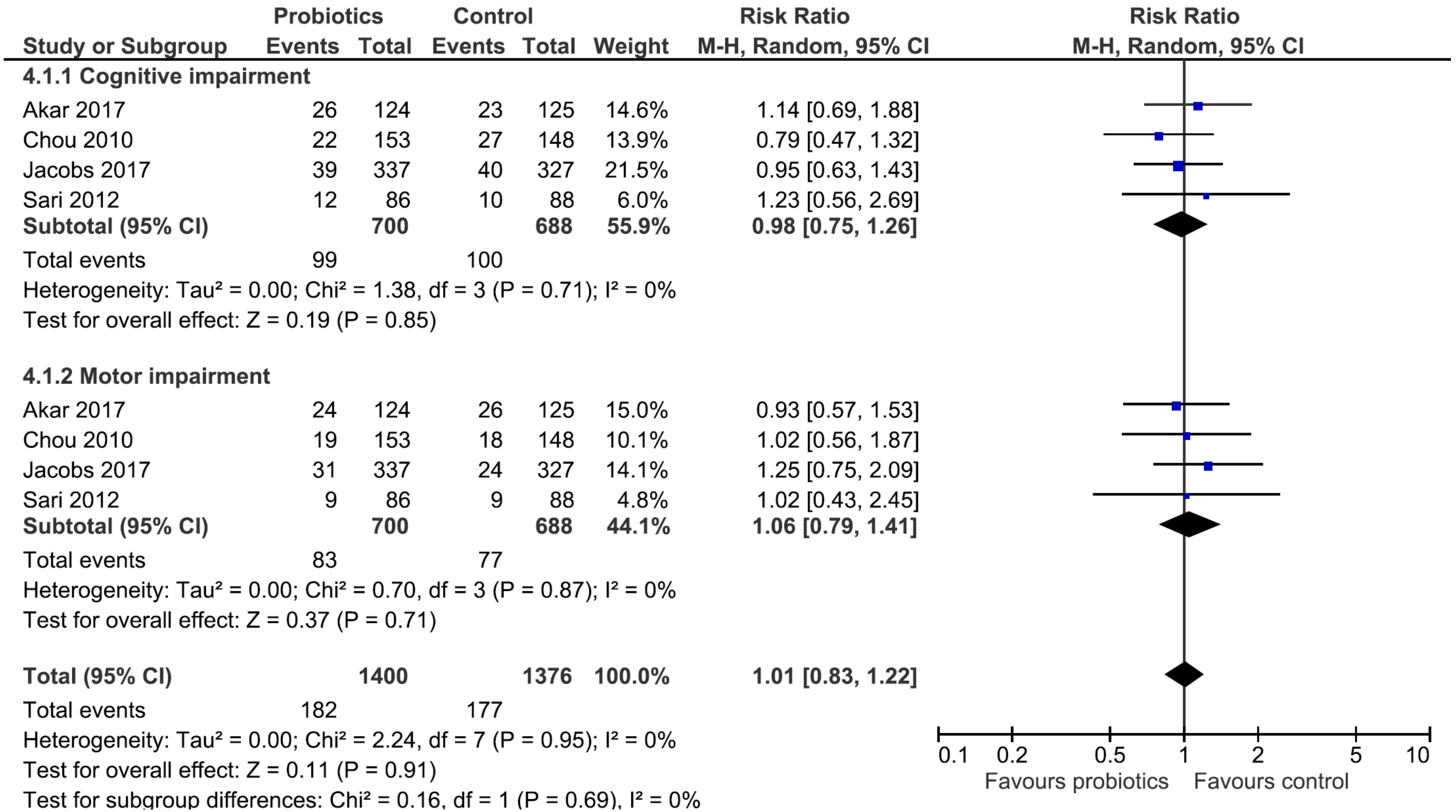

Supplemental Figure 7: Forest plot illustrating effect of probiotics on mean cognitive and motor scores in preterm infants.

	Probiotics			Control				Mean Difference
Study or Subgroup	Mean	SD	Total	Mean	SD	Total	Weight	IV, Random, 95% CI
**4.2.1 Mean cognitive scores**								
Akar 2017	81	12.5	124	82	12.5	125	11.7%	-1.00 [-4.11, 2.11]
Chou 2010	87.9	18.8	153	88.5	18.4	148	6.4%	-0.60 [-4.80, 3.60]
Jacobs 2017	100.4	17.1	337	99.2	15.1	327	18.8%	1.20 [-1.25, 3.65]
Sari 2012	90.7	15.5	86	90.4	14.5	88	5.7%	0.30 [-4.16, 4.76]
Totsu 2018	90.6	12.5	54	91.1	14.1	65	4.9%	-0.50 [-5.28, 4.28]
**Subtotal (95% CI)**			**754**			**753**	**47.4%**	**0.13 [-1.41, 1.67]**
Heterogeneity: Tau² = 0.00; Chi² = 1.43, df = 4 (P = 0.84); I² = 0%								
Test for overall effect: Z = 0.17 (P = 0.87)								
**4.2.2 Mean motor scores**								
Akar 2017	80	10.5	124	79	9.7	125	17.9%	1.00 [-1.51, 3.51]
Chou 2010	86.4	18.6	153	87.9	17.1	148	6.9%	-1.50 [-5.53, 2.53]
Jacobs 2017	102.3	11.6	337	100.7	16.8	327	23.3%	1.60 [-0.60, 3.80]
Sari 2012	95.4	17.2	86	93.2	16.4	88	4.5%	2.20 [-2.80, 7.20]
**Subtotal (95% CI)**			**700**			**688**	**52.6%**	**1.04 [-0.43, 2.50]**
Heterogeneity: Tau² = 0.00; Chi² = 1.98, df = 3 (P = 0.58); I² = 0%								
Test for overall effect: Z = 1.39 (P = 0.16)								
**Total (95% CI)**			**1454**			**1441**	**100.0%**	**0.61 [-0.45, 1.67]**
Heterogeneity: Tau² = 0.00; Chi² = 4.11, df = 8 (P = 0.85); I² = 0%								
Test for overall effect: Z = 1.12 (P = 0.26)								
Test for subgroup differences: Chi² = 0.70, df = 1 (P = 0.40), I² = 0%								

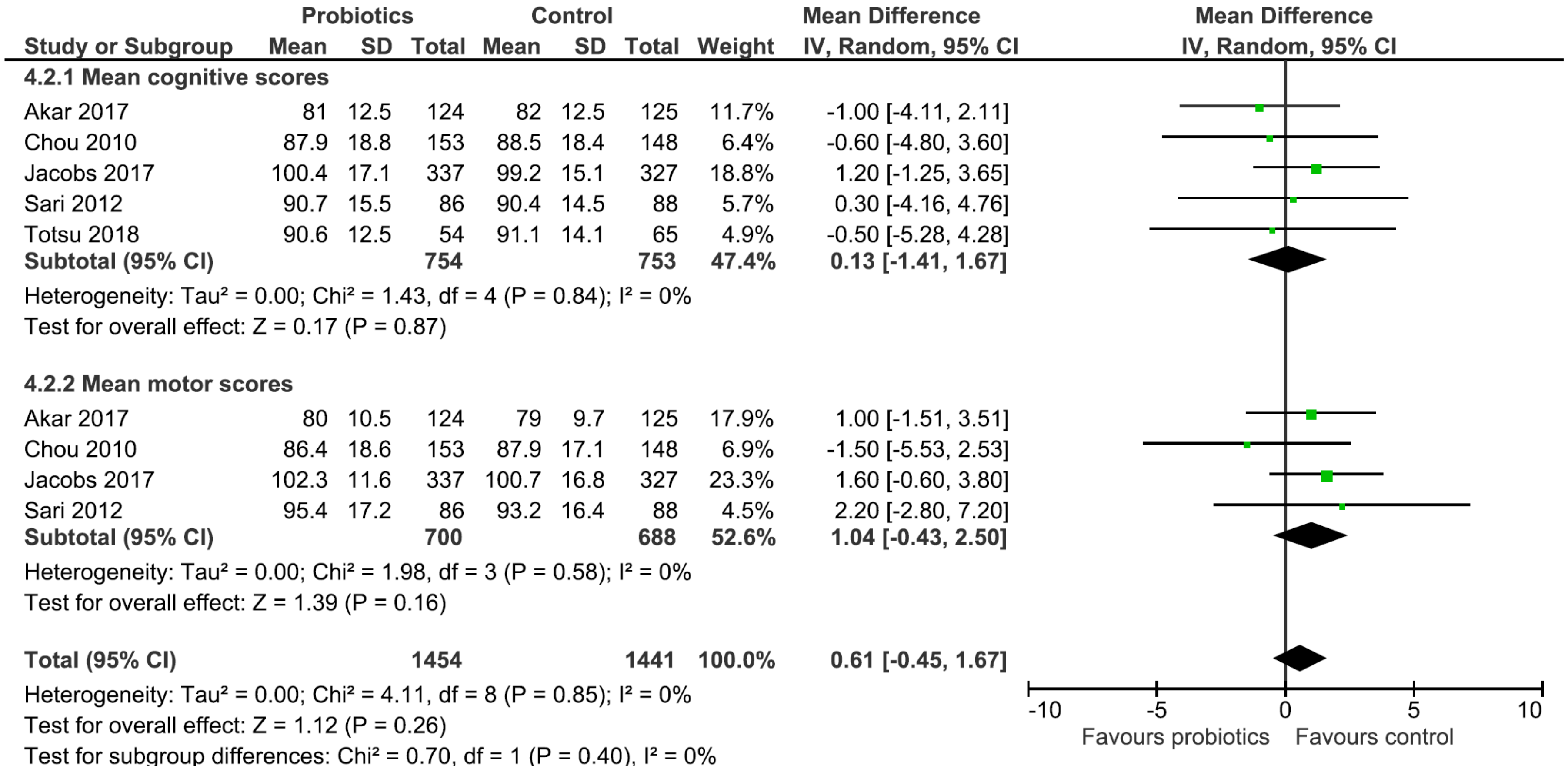

Supplemental Figure 8: Forest plot illustrating effect of probiotics on increase in short-term weight gain in <28 weeks preterm infants.

Study or Subgroup	Std. Mean Difference	SE	Probiotics Total	Control Total	Weight	Std. Mean Difference IV, Random, 95% CI
Al Hosni 2012	0.409	0.2012	50	51	31.9%	0.41 [0.01, 0.80]
Van Niekerk 2014	-0.1457	0.191	54	56	33.3%	-0.15 [-0.52, 0.23]
Wejryd 2019	-0.1035	0.1798	63	61	34.9%	-0.10 [-0.46, 0.25]
**Total (95% CI)**			**167**	**168**	**100.0%**	**0.05 [-0.29, 0.38]**

Heterogeneity: Tau² = 0.05; Chi² = 4.94, df = 2 (P = 0.08); I² = 60%

Test for overall effect: Z = 0.26 (P = 0.79)

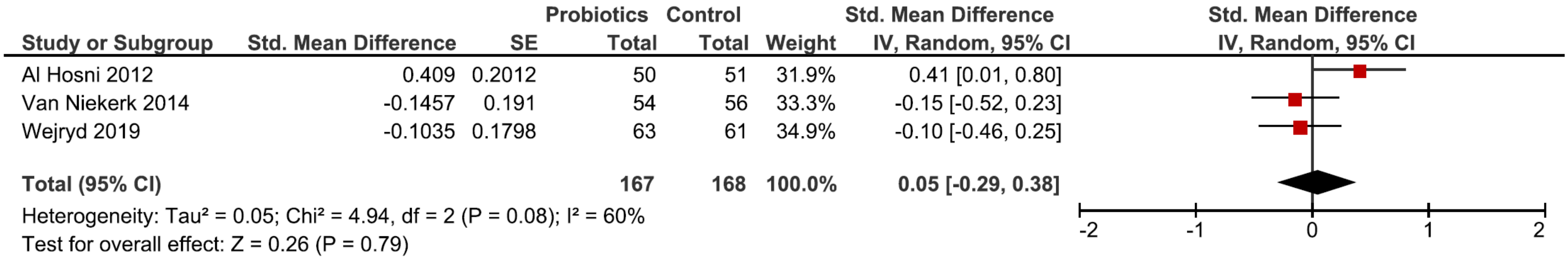

Supplemental Figure 9: Forest plot illustrating effect of probiotics on increase in short-term length gain in <28 weeks preterm infants.

Study or Subgroup	Std. Mean Difference	SE	Probiotics Total	Control Total	Weight	Std. Mean Difference IV, Random, 95% CI
Van Niekerk 2014	-0.1246	0.1909	54	56	47.0%	-0.12 [-0.50, 0.25]
Wejryd 2019	-0.0845	0.1797	63	61	53.0%	-0.08 [-0.44, 0.27]
**Total (95% CI)**			**117**	**117**	**100.0%**	**-0.10 [-0.36, 0.15]**

Heterogeneity: Tau² = 0.00; Chi² = 0.02, df = 1 (P = 0.88); I² = 0%
Test for overall effect: Z = 0.79 (P = 0.43)

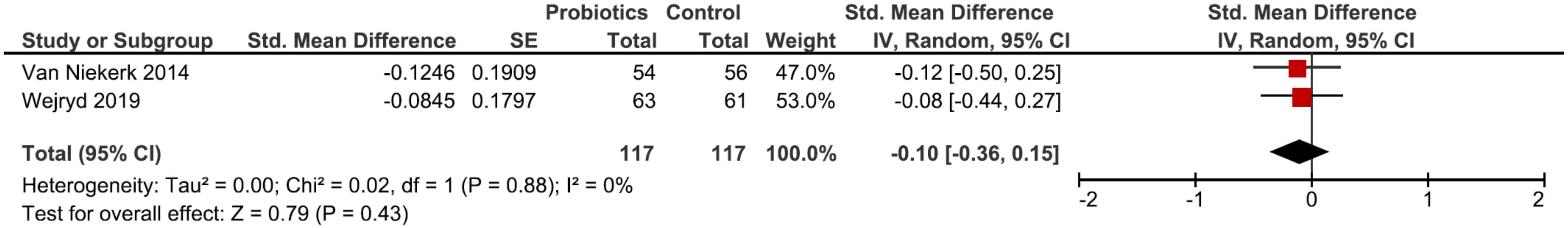

Supplemental Figure 10: Forest plot illustrating effect of probiotics on increase in short-term weight gain in <28 weeks preterm infants.

Study or Subgroup	Std. Mean Difference	SE	Probiotics Total	Control Total	Weight	Std. Mean Difference IV, Random, 95% CI
Van Niekerk 2014	-0.1089	0.1909	54	56	47.6%	-0.11 [-0.48, 0.27]
Wejryd 2019	0.1791	0.18	63	61	52.4%	0.18 [-0.17, 0.53]
**Total (95% CI)**			**117**	**117**	**100.0%**	**0.04 [-0.24, 0.32]**

Heterogeneity: Tau² = 0.01; Chi² = 1.20, df = 1 (P = 0.27); I² = 17%
Test for overall effect: Z = 0.29 (P = 0.77)

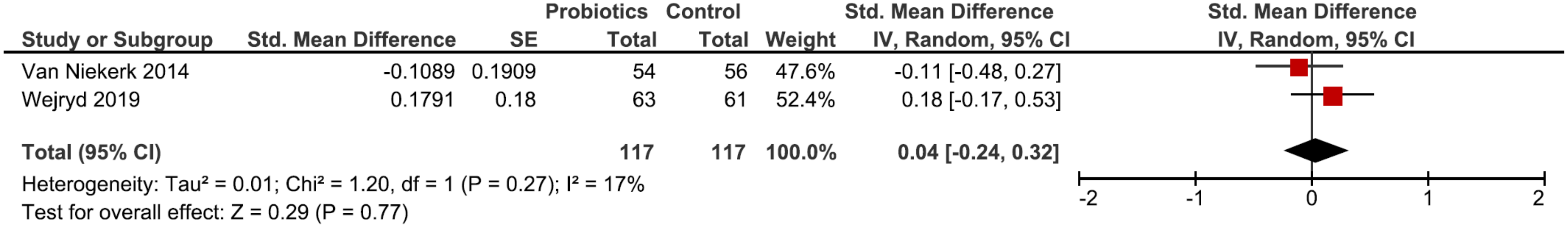

Supplemental Figure 11: Quality assessment of included studies using Cochrane risk of bias (ROB-2) tool.

Studies with growth as outcome	Randomization process	Deviations from intended interventions	Missing outcome data	Measurement of the outcome	Selection of the reported result	Overall
Spreckels PROPELS 2021	+	+	?	+	+	!
Cui et al 2019	+	+	+	+	+	+
Oshiro et al 2019	–	+	+	+	+	–
Wejryd et al2019	+	+	+	+	+	+
Indrio 2017	+	+	+	+	+	+
Shashidhar et al 2017	+	+	+	+	+	+
Sukanyaa et al 2017	?	?	+	+	?	!
Hays et al 2016	+	+	+	+	+	+
Xu et al 2016	+	+	+	+	+	+
Choudhury et al 2015	?	?	+	+	+	!
Dilli et al 2015	+	+	+	+	+	+
Shadkam et al 2015	+	+	+	+	+	+
Patole et al 2014	+	+	+	?	?	!
Totsu et al 2014	+	+	+	+	+	+
E.Van Niekerk et al 2014	+	+	+	+	+	+
Demirel et al 2013	+	+	+	?	?	!
Jacobs et al 2013	+	+	+	+	+	+
Serce et al 2013	+	+	+	+	+	+
Al-Hosni et al 2012	+	+	+	+	+	+
Chrzanowska-Liszewska et al 2012	+	+	+	+	+	+
Sari et al 2011	+	+	+	+	+	+
Indrio et al 2008	?	?	+	+	+	!
Mohan et al 2008	+	+	+	+	+	+
Stratiki et al 2007	+	+	+	+	+	+
Bin-Nun et al 2005	?	+	?	–	–	–
Costalos et al 2003	+	+	+	+	+	+
Kitajima et al 1997	?	?	+	?	–	–
Reuman et al 1986	+	+	+	+	+	+
Totsu et al 2018	+	+	–	+	+	–
Jacobs et al 2017	+	+	+	+	+	+
Sari et al 2012	+	+	–	+	+	–
Chou et al 2011	+	+	+	+	+	+

+ Low risk
? Some concerns
– High risk

Studies with neurodevelopment as outcome	Randomization process	Deviations from intended interventions	Missing outcome data	Measurement of the outcome	Selection of the reported result	Overall
Agrawal et al 2020	+	+	–	+	+	–
Akar et al 2016	+	+	–	+	+	–
Romeo et al 2011	?	+	+	+	+	!
Totsu et al 2018	+	+	–	+	+	–
Jacobs et al 2017	+	+	+	+	+	+
Sari et al 2012	+	+	–	+	+	–
Chou et al 2011	+	+	+	+	+	+

Supplemental Figure 12: Funnel plot illustrating publication bias.

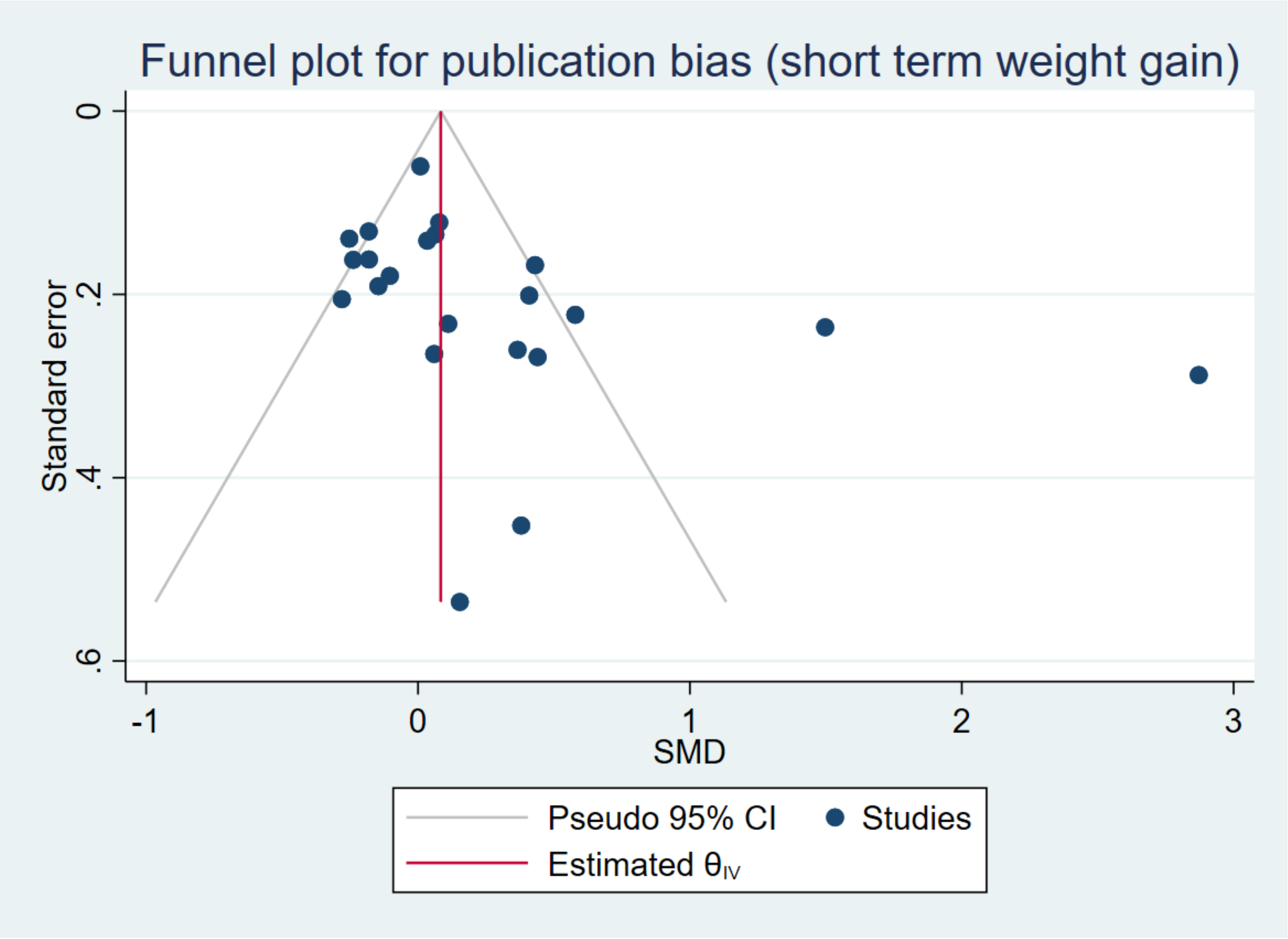

Supplement: Supplementary file 1 — Supplemental tables and figures [file 41430_2023_1270_MOESM1_ESM.pdf]
